# Self-managed, computerised speech and language therapy for patients with chronic aphasia post-stroke compared with usual care or attention control (Big CACTUS): a multicentre, single-blinded, randomised controlled trial

**DOI:** 10.1016/S1474-4422(19)30192-9

**Published:** 2019-09

**Authors:** Rebecca Palmer, Munyaradzi Dimairo, Cindy Cooper, Pam Enderby, Marian Brady, Audrey Bowen, Nicholas Latimer, Steven Julious, Elizabeth Cross, Abualbishr Alshreef, Madeleine Harrison, Ellen Bradley, Helen Witts, Tim Chater

**Affiliations:** a Deparment of Health Service Research, School of Health and Related Research (ScHARR), The University of Sheffield, Sheffield, UK; b Department of Health Economics and Decision Science, School of Health and Related Research (ScHARR), The University of Sheffield, Sheffield, UK; c Department of Design, Trials and Statistics, School of Health and Related Research (ScHARR), The University of Sheffield, Sheffield, UK; d Clinical Trials Research Unit (CTRU), ScHARR, The University of Sheffield, Sheffield, UK; e Nursing, Midwifery and Allied Health Professions Research Unit, Glasgow Caledonian University, Glasgow, UK; f Division of Neuroscience and Experimental Psychology, The University of Manchester MAHSC, Manchester, UK; g Speech and Language Therapy, Derbyshire Community Health Services NHS Foundation Trust, Chesterfield, UK

## Abstract

**Background:**

Post-stroke aphasia might improve over many years with speech and language therapy; however speech and language therapy is often less readily available beyond a few months after stroke. We assessed self-managed computerised speech and language therapy (CSLT) as a means of providing more therapy than patients can access through usual care alone.

**Methods:**

In this pragmatic, superiority, three-arm, individually randomised, single-blind, parallel group trial, patients were recruited from 21 speech and language therapy departments in the UK. Participants were aged 18 years or older and had been diagnosed with aphasia post-stroke at least 4 months before randomisation; they were excluded if they had another premorbid speech and language disorder caused by a neurological deficit other than stroke, required treatment in a language other than English, or if they were currently using computer-based word-finding speech therapy. Participants were randomly assigned (1:1:1) to either 6 months of usual care (usual care group), daily self-managed CSLT plus usual care (CSLT group), or attention control plus usual care (attention control group) with the use of computer-generated stratified blocked randomisation (randomly ordered blocks of sizes three and six, stratified by site and severity of word finding at baseline based on CAT Naming Objects test scores). Only the outcome assessors and trial statistician were masked to the treatment allocation. The speech and language therapists who were doing the outcome assessments were different from those informing participants about which group they were assigned to and from those delivering all interventions. The statistician responsible for generating the randomisation schedule was separate from those doing the analysis. Co-primary outcomes were the change in ability to retrieve personally relevant words in a picture naming test (with 10% mean difference in change considered a priori as clinically meaningful) and the change in functional communication ability measured by masked ratings of video-recorded conversations, with the use of Therapy Outcome Measures (TOMs), between baseline and 6 months after randomisation (with a standardised mean difference in change of 0·45 considered a priori as clinically meaningful). Primary analysis was based on the modified intention-to-treat (mITT) population, which included randomly assigned patients who gave informed consent and excluded those without 6-month outcome measures. Safety analysis included all participants. This trial has been completed and was registered with the ISRCTN, number ISRCTN68798818.

**Findings:**

From Oct 20, 2014, to Aug 18, 2016, 818 patients were assessed for eligibility, of which 278 (34%) participants were randomly assigned (101 [36%] to the usual care group; 97 [35%] to the CSLT group; 80 [29%] to the attention control group). 86 patients in the usual care group, 83 in the CSLT group, and 71 in the attention control group contributed to the mITT. Mean word finding improvements were 1·1% (SD 11·2) in the usual care group, 16·4% (15·3) in the CSLT group, and 2·4% (8·8) in the attention control group. Word finding improvement was 16·2% (95% CI 12·7 to 19·6; p<0·0001) higher in the CSLT group than in the usual care group and was 14·4% (10·8 to 18·1) higher than in the attention control group. Mean changes in TOMs were 0·05 (SD 0·59) in the usual care group (n=84), 0·04 (0·58) in the CSLT group (n=81), and 0·10 (0·61) in the attention control group (n=68); the mean difference in change between the CSLT and usual care groups was –0·03 (–0·21 to 0·14; p=0·709) and between the CSLT and attention control groups was –0·01 (–0·20 to 0·18). The incidence of serious adverse events per year were rare with 0·23 events in the usual care group, 0·11 in the CSLT group, and 0·16 in the attention control group. 40 (89%) of 45 serious adverse events were unrelated to trial activity and the remaining five (11%) of 45 serious adverse events were classified as unlikely to be related to trial activity.

**Interpretation:**

CSLT plus usual care resulted in a clinically significant improvement in personally relevant word finding but did not result in an improvement in conversation. Future studies should explore ways to generalise new vocabulary to conversation for patients with chronic aphasia post-stroke.

**Funding:**

National Institute for Health Research, Tavistock Trust for Aphasia.

## Introduction

Aphasia affects the comprehension and expression of speech, reading, and writing, which can affect a patient's mood, cause them to feel isolated, and can alter their relationships with family and their role within the community.^1^, ^2^ More than one third of stroke survivors acquire this disorder,^1^ of whom 30–43% remain affected long-term.^1^

A Cochrane review^2^ suggested that patients with chronic aphasia (>6 months) could improve their language skills with speech and language therapy. A subsequent randomised controlled trial^3^ added to this evidence, showing that at least 10 h a week of speech and language therapy for 3 weeks was effective in improving functional outcomes in chronic aphasia, with treatment effects remaining stable after 6 months. However, due to the health-care costs in providing therapeutic amounts of speech and language therapy in the longer term^4^, ^5^ and that patients with aphasia often want more therapy than they are able to access,^4^ an effective and low-cost approach to delivering speech and language therapy is required.

Systematic reviews^2^, ^6^ of small randomised studies (n=18–55) suggest computer therapy might be an effective way to provide speech and language therapy to patients with chronic aphasia. A pilot study of 34 patients^7^ suggested that a self-managed, computerised, word finding therapy approach was feasible, acceptable, and had potential clinical effectiveness and cost-effectiveness.^7^, ^8^

We assessed the clinical and cost-effectiveness of self-managed computer speech and language therapy (CSLT) for word finding in patients with chronic aphasia lasting at least 4 months after stroke.^9^ The key hypothesis was that CSLT plus usual care would improve word finding ability and as a result would improve functional communication in conversation compared with usual care alone or attention control plus usual care.

**Research in context**

**Evidence before this study**

People with chronic aphasia post-stroke often want and require more speech and language therapy than they can access. We searched the Cochrane Library and Medline for systematic reviews and randomised controlled trials of interventions or service delivery for chronic aphasia in post-stroke populations, using the search terms “aphasia OR dysphasia AND therapy OR treatment AND stroke”. We included papers published in English between Jan 1, 2010, and Feb 8, 2019. Studies of drug therapies, electrical stimulation, alternative medicine, and randomised controlled trials without between-group comparisons were excluded. The FCET2EC trial (From Controlled Experimental Trial 2 Everyday Communication) was a high-quality randomised controlled trial of 158 patients with post-stroke aphasia, with a low risk of bias due to computer generated randomisation and masked outcome measure assessors. This trial showed the effectiveness of speech and language therapy (≥10 h per week for 3 weeks) for patients with chronic aphasia compared with controls on a waiting list for therapy, with benefits still evident at 6 months after treatment, and it supports findings from smaller trials (n=20 or fewer per group) that chronic post-stroke aphasia can improve with speech and language therapy. Due to resource limitations, computer therapy has been explored as a potentially efficient method of delivering sufficient quantities of speech and language therapy. Systematic reviews of computer-based speech and language therapy for patients with aphasia suggest that it might be more effective than no therapy and just as effective as face-to-face therapy. However, the quality of the studies included in the systematic reviews was low, with small sample sizes (n=55 or fewer). Only two of the studies assessed maintenance of treatment effect and only one considered the effect on functional communication. One small scale pilot study (n=34) included health economic evaluation, which suggested potential cost-effectiveness of CSLT.

**Added value of this study**

The Big CACTUS trial is the first multicentre randomised controlled trial in patients with post-stroke chronic aphasia (>6 months) to assess both the clinical and cost-effectiveness of self-managed word finding therapy with specialist aphasia computer software. It is also the first randomised controlled trial powered to detect a meaningful difference in clinical outcomes. Our trial shows that it is feasible and acceptable to provide computerised therapy in routine clinical contexts increasing the availability of speech and language therapy provision. The findings provide robust evidence that patients with chronic aphasia can achieve improvement in their word finding, which is maintained after a 6-month follow-up period through self-managed practice with a computer. However, the improvement did not generalise to conversation. Health economic evaluation suggests that this low-cost intervention is more likely to be cost-effective for patients with mild and moderate word finding difficulties than for all patients with chronic aphasia.

**Implications of all the evidence**

Self-managed computer therapy can be integrated into speech and language therapy services to enable availability of effective amounts of repetitive practice to improve and maintain retrieval of treated words in people with chronic aphasia. However, new ways to support generalisation of new vocabulary into everyday conversation must be explored.

## Methods

### Study design and participants

Big CACTUS was a pragmatic, superiority, three-arm, individually randomised, single-blind, parallel group, randomised controlled trial. Patients were recruited from 21 community speech and language therapy departments within 20 UK National Health Service (NHS) Trusts (appendix p 21). We designed a pragmatic study, using the pragmatic-explanatory continuum indicator summary (PRECIS) domains (appendix p 105).^10^

Patients were eligible if they were aged 18 years or older and had aphasia confirmed by a speech and language therapist after one or more strokes at least 4 months before randomisation. Patients had word finding difficulties (defined by a score of 5–43 out of 48 on the Comprehensive Aphasia Test [CAT] Naming Objects test),^11^ could perform a simple matching task on the StepByStep computer program^12^ with at least 50% accuracy (score of 5 out of 10 or higher), and could repeat at least 50% of words in a repetition task on StepByStep (score of 5 out of 10 or higher). Patients were excluded if they had a premorbid speech and language disorder caused by a neurological deficit other than stroke, if they required treatment for a language other than English (because the StepByStep software used for therapy was in English), or if they were currently using a word finding computer program, including StepByStep.

Eligible patients were identified by practising speech and language therapists from past and current caseloads and voluntary sector support groups. Therapists used a Consent Support Tool^13^ to identify the level of support required for each patient with aphasia to provide written informed consent and to identify those who required a carer to provide written consent or a declaration of belief that they wished to take part.

Ethics approval was obtained from Leeds West NHS research ethics committee [reference 13/YH/0377] and Scotland A research ethics committee [reference 14/SS/0023]. The full protocol is available online. No major changes to methods or design were made once the trial had started.

### Randomisation and masking

We used a centralised web-based randomisation system to randomly assign patients to one of three interventions: usual care, CSLT plus usual care (CSLT group), or attention control plus usual care (attention control group), using a fixed 1:1:1 allocation ratio. This system used stratified block randomisation with randomly ordered blocks of sizes three and six, stratified by site and severity of word finding at baseline based on CAT Naming Objects test scores:^11^ mild (31–43), moderate (18–30), and severe (5–17). Only the independent randomisation statistician knew the block sizes, which were not disclosed until after the trial ended. The statistician used the restricted-access randomisation system to specify the randomisation details and to generate the randomisation schedule, which was retained within the system. Therapists randomly assigned patients using the online system and disclosed the allocation to the patient.

We could not mask patients and treating therapists to intervention allocation. However, the therapists performed baseline assessments before randomisation and therefore completed these masked to treatment allocation. The therapists who performed baseline assessments also provided CSLT and attention control interventions. Therefore, we trained separate therapists (outcome assessors), masked to intervention allocation, to do follow-up assessments at each site. Video recordings of conversations between patients and therapist assessors at baseline and follow-up were rated independently of the therapist team who delivered the intervention by speech and language therapists who were not involved in the trial, ensuring raters were masked to intervention allocation and timepoint of measurement. Further details regarding masking and assessment of its success are described in the appendix (p 11, 98, 100).

### Procedures

The interventions of this trial are described in detail using the TIDieR template (table 1).^14^ Patients in all three intervention groups were provided with usual care. To establish what constituted as usual care, speech and language therapy was recorded for 3 months before patients who had chronic aphasia longer than 4 months after stroke were randomised.^5^ Usual speech and language therapy continued to be provided to patients in all intervention groups to assess the effectiveness of CSLT in addition to usual therapy currently received rather than in place of it.

**Table 1:** TIDieR template of trial interventions

	**Usual care**	**CSLT**	**Attention control**
Why?	To improve the communication of people with aphasia and to reduce the impact of aphasia on their lives.	To provide increased amounts of SLT long-term for people with word finding difficulties post-stroke. The aim was to adhere to key principles of experience-dependent neuroplasticity (salience, repetition, feedback).^15^	To differentiate the SLT components of CSLT from additional activity and attention received.
What?	Assessment and review of language abilities and their impact, rehabilitation of different language domains, enabling communication using communication aids or compensatory strategies, or support for mood, confidence, work, family, form completion, and information provision.^5^	Word finding exercises were provided on a computer (PC, laptop, or tablet) owned by the participant or loaned by the NHS trust. The StepByStep aphasia software^12^ was used as it can be tailored to the individual's needs, allows presentation of personally relevant words (eg, grandchildren's names), encourages repetitive practice, and provides feedback about whether the words used are correct.	Puzzle books (Sudoku, spot the difference, mazes, word searches, cross words, colouring).
Who provided?	Speech and language therapists or therapy assistants.	Speech and language therapists provided the software. Volunteers or therapy assistants provided encouragement and support to practise computer exercises, practised using new words in functional contexts, and fed back on progress to the therapist.	Speech and language therapists provided the first puzzle book and a research assistant from the central Big CACTUS team sent out books thereafter. Monthly telephone calls were made by the research team to provide support and identify the type of puzzle book to be sent next.
How?	Face-to-face on a one-to-one basis or in a group.	Practice of the word finding exercises on the computer was self-managed by participants.	Puzzle books were completed independently by the participants.
Where?	Participants' own homes, or outpatient or community clinical facility.	Participants' own homes.	Participants' own homes.
When and how much?	60% of participants were not in receipt of SLT in the three months prior to randomisation. The remaining 40% received a median average of 5 h 20 minutes over 3 months in 1-hour sessions every two weeks. This decreased with time post-stroke.^5^	20–30 min practice daily was recommended over a 6-month period (based on feasibility shown in the pilot study).^7^ Volunteers or therapists assistants were asked to visit for at least 1 h once a month.	A recommendation of completing one puzzle daily over a 6-month period was made. The Aphasia Patient and Public Involvement group considered one puzzle could take a similar amount of time to complete as the daily time spent using the computer exercises. Telephone calls were made monthly (need for new puzzle books was established during this call and requests for new books could be made between calls).
Tailoring	Tailored to individual needs and preferences at the discretion of the treating therapist.	Therapists chose therapy exercises based on the results of baseline language assessments. They also worked with the participants and their families to identify 100 words of personal relevance for therapy practice.	Therapists matched the first book to the participant's abilities and interests. Subsequent books were provided according to feedback from the participant or carer.
Modifications	No modifications were requested by the trial team.	Therapists were advised that they could set the 100 words up in stages rather than all at once.	No modifications were made.
How well?	Fidelity to the provision of usual care for all three groups was measured by recording the amounts received by each group throughout the trial and checking they were similar to each other (there was an expectation that average amounts of usual care would decrease across the trial period as the strokes became longer ago and SLT commonly decreases over time).^5^	Therapists were provided with 1-day training on the intervention. An intervention manual was provided.* Fidelity to practice adherence and quality of therapy delivery by the therapists and volunteers or therapy assistants was assessed (see appendix p 106).	Adherence was measured using number of puzzle books sent and number of telephone contacts made (minimum of four books and four calls expected).

CSLT=computerised speech and language therapy. NHS=National Health Service. PC=personal computer. SLT=speech and language therapy.

* For the intervention manual, please see online.

Patients assigned to the CSLT group completed daily, self-managed, word-finding exercises on a computer at home, which were tailored to the needs of the individual patient by a qualified speech and language therapist experienced in working with patients with stroke in routine clinical practice. Each patient chose 100 words that were relevant to them, before they were randomly assigned to one of the intervention groups, which were then used for computerised word finding practice. Practice was supported by a therapy assistant or volunteer under the supervision of the therapist for 6 months.

Patients in the attention control group were asked to complete paper-based puzzle book activities (eg, sudoku, spot the difference, word searches, or colouring) on a daily basis and received supportive telephone calls from the research team once a month. The type and difficulty of the puzzle book was established by the therapist who did the baseline assessments and the research team sent new books each month on the basis of the phone call discussions. If they wished, patients were able to continue using CSLT after the 6-month supported period if they still had access to it (given that CSLT often required laptops that had been lent, and might have subsequently been required by a new patient). Participants in the attention control group were also able to continue completing the paper-based puzzle books after 6 months if they wished by purchasing them themselves from high-street shops.

Study visits were scheduled at baseline before randomisation, and then at 6 months, 9 months, and 12 months after randomisation. Qualified therapists from the local speech and language therapy team visited patients in their own homes to carry out assessments. Therapists aimed to complete follow-up visits within 1 month after the scheduled timepoint. Clinical assessment comprised a CAT comprehension test (baseline visit only);^11^ a picture naming test of 100 personally relevant words (co-primary outcome measurement); a 10 min conversation between the therapist and patient, structured around topics of personal importance to the patient, which was videoed and later assessed using the activity scale of the Therapy Outcome Measures (TOMs; a co-primary outcome measurement),^16^ once all of the conversations had been made and their presentation could be randomly assigned to masked raters. The number of treated words used in conversation were also counted (secondary outcome) by two members of the central research team (who were masked to the timepoint of the video conversation and the group allocation of the participant). Further secondary outcome measurements included the Communication Outcomes After Stroke (COAST)^17^ questionnaire (the key secondary outcome according to our Hochberg statistical testing procedure), self-rated by the patient; the CAT Naming Objects test;^11^ and the CarerCOAST^18^ questionnaire, self-rated by the carer about themselves and the patient (appendix p 14).

For the health economic outcome evaluation, an accessible (aphasia-friendly) variant of the EuroQoL instrument, a five-dimensional 5-level generic instrument (EQ-5D-5L) that measures quality of life (QoL), was developed to facilitate patient self-completion.^19^ When a carer was available, the standard EQ-5D-5L^20^ was also completed on behalf of patients. To assess the health-related QoL of carers, carers completed the standard EQ-5D-5L and CarerQoL questionnaires.

Assessors asked about adverse events and serious adverse events 3 months after randomisation by telephone and at all follow-up visits. Assessors asked whether any events had occurred (not necessarily side-effects of the intervention), using a predefined prompt sheet, but if a therapist or member of the research team became aware of any events between these assessments, these were also recorded. The events were then classified into adverse events and serious adverse events using International Conference on Harmonisation Good Clinical Practice classifications. Patients of the CSLT group were additionally sent postal questionnaires each month, between randomisation and the 6-month follow-up, to report whether they had experienced any negative effects of CSLT.

### Outcomes

Co-primary outcomes were the change in ability to retrieve vocabulary of personal relevance (impairment), measured by a picture naming test of 100 personally relevant words (appendix pp 13–15), and the change in functional communication ability (activity) measured by masked ratings of videoed conversations, with the use of the TOMs activity scale, between baseline and 6 months after randomisation.

The key secondary outcome was change in self-perception of communication, social participation, and QoL between baseline and 6 months after randomisation, measured by COAST. Maintenance of treatment effect was assessed by repeating the assessments used to measure co-primary and key secondary outcomes at 9-month and 12-month follow-ups. Additional secondary outcomes assessed at 6-month, 9-month, and 12-month follow-ups were change in the number of treated words (ie, words used in treatment, as chosen by the participants before randomisation) used in videoed conversations (use of specific words practised), generalisation to untreated words (ie, words that were not used in therapy) measured by the CAT Naming Objects test, change in carer's perception of patient's communication and social participation (assessed using the first 15 items of the CarerCOAST), and change in carer's QoL (assessed using the last five items of the CarerCOAST).

Safety outcomes included negative effects of CSLT assessed monthly for 6 months after randomisation through postal questionnaire and adverse events and serious adverse events reported at 3, 6, 9, and 12 months. See full protocol for negative effects, adverse events, and serious adverse events recorded.

Health-related QoL (HRQoL) was measured at baseline, 6, 9, and 12 months by the patients who completed the accessible EQ-5D-5L, and for those who had a carer, the EQ-5D-5L was also completed by their carer on their behalf. The EQ-5D-5Ls were used to calculate utility scores and quality-adjusted life-years (QALYs), and resource use was measured to allow estimation of cost-effectiveness. Carer HRQoL was measured using the EQ-5D-5L and the CarerQoL.

### Statistical analysis

We aimed to recruit 285 participants (95 per group), which had 90% power for a 5% two-sided test to address both co-primary objectives. Based on the consensus by therapists on the trial team and the Aphasia Patient and Public Involvement group, we considered a 10% mean difference in change in word finding (SD 17·38 from analysis of covariance model) to be a minimal, clinically important difference. We used a TOMs standardised effect size of 0·45, similar to the ACTNoW study^21^ of aphasia therapy. Sample size was adjusted for a 15% dropout rate observed in the pilot trial^7^ and inflated by 1·14^22^, ^23^ to account for the variance being estimated from a small pilot trial (n=34)^7^ and 0·5 correlation between baseline and TOMs outcome. This sample size of 285 participants had 83% power for a 5% two-sided test to address the key secondary objective, assumed a 7·2% mean difference (SD 18) as clinically worthwhile, and 0·5 correlation between baseline and COAST outcome. See the statistical analysis plan for more details. We terminated recruitment after 278 of the planned 285 participants had been enrolled, because the observed dropout rate (9%) was lower than anticipated (15%) and the study had the desired statistical power to address the co-primary objectives.

We based the analysis of the primary outcomes (6 months after randomisation) on a modified intention-to-treat (mITT) principle, which included randomly assigned patients who gave informed consent and excluded those without 6-month outcome measures. We based analysis on the treatment patients were randomly assigned to. We excluded deaths before assessment at 6 months in all clinical effectiveness analyses because the association between the interventions and increased risk of mortality was viewed as extremely unlikely.

We used a multiple linear regression model adjusted for baseline outcome measures and fixed stratification factors (site and severity of word finding at baseline). We estimated the mean difference in change between the CSLT and usual care groups, and CSLT and attention control groups, with associated 95% CIs and p values. We analysed all clinical outcomes at 9 and 12 months in a similar manner. Co-primary, key secondary, and key multiple treatment comparisons (CSLT *vs* usual care and CSLT *vs* attention control) are the sources of multiple hypothesis testing. We used a Hochberg hierarchical multiple testing procedure to control for the possibility of making false claims about clinical benefit at 5% significance level on the co-primary outcomes and key secondary outcome at 6 months.^24^ To help with the clinical interpretation of superiority of the CSLT intervention, we used the Hochberg decision tree (appendix p 16). We only report p values where appropriate in accordance with the decision tree.

The primary safety analysis included all randomly assigned patients with informed consent and according to the treatment they received. We summarised the proportion of patients who experienced any negative effects of CSLT by classification. We modelled the number of repeated negative effects per classification using a negative binomial regression model accounting for overdispersion and follow-up period to estimate the incidence with 95% CI. We summarised the proportion of patients who experienced any adverse event or serious adverse event by group and modelled repeated events using a negative binomial regression model to estimate the incidence in each group, and incidence rate ratio (IRR) between groups with associated 95% CI, accounting for overdispersion and follow-up period.

We performed per-protocol analysis on the co-primary outcomes and key secondary outcome at 6 months for patients who adhered to the key components of the interventions, including achieving a minimum amount of recommended practice and having access to at least 4 h of support from a volunteer or therapy assistant up to the 6-month visit (appendix pp 12, 93). We explored potential heterogeneity in intervention effects in prespecified subgroups defined by severity of word finding difficulty, comprehension ability, and length of time post-stroke. We also explored the effect of missing data using multiple imputation strategies. See the appendix (pp 12–17) for detailed methods, including computation of summary measures of all clinical outcomes for analysis.

The health economic analysis was a model-based cost-utility analysis adopting a lifetime time horizon and an NHS payer perspective. Cost-effectiveness is expressed in terms of the incremental cost-effectiveness ratio (ICER)—ie, incremental cost per QALY gained. We calculated the cost of the interventions by following a three-stage process: identification of resource use, measurement, and valuation using national reference unit costs.^25^ Secondary analyses included analysis of subgroups and analyses in which QALY gains were based upon EQ-5D-5L responses from carers by proxy. The health economic analysis will be fully detailed in a future publication. For all the statistical analysis, Stata version 15.1 was used.

The study was overseen by an independent data management and ethics committee. The trial was registered with the ISRCTN registry, number ISRCTN68798818.

### Role of the funding source

The National Institute for Health Research (NIHR), a funder of the study, commissioned the study and requested inclusion of an attention control group. The funders of the study had no further role in study design, data collection, data analysis, data interpretation, or writing of the paper. The corresponding author had full access to all the study data and had final responsibility for the decision to submit for publication.

## Results

**Figure 1:**
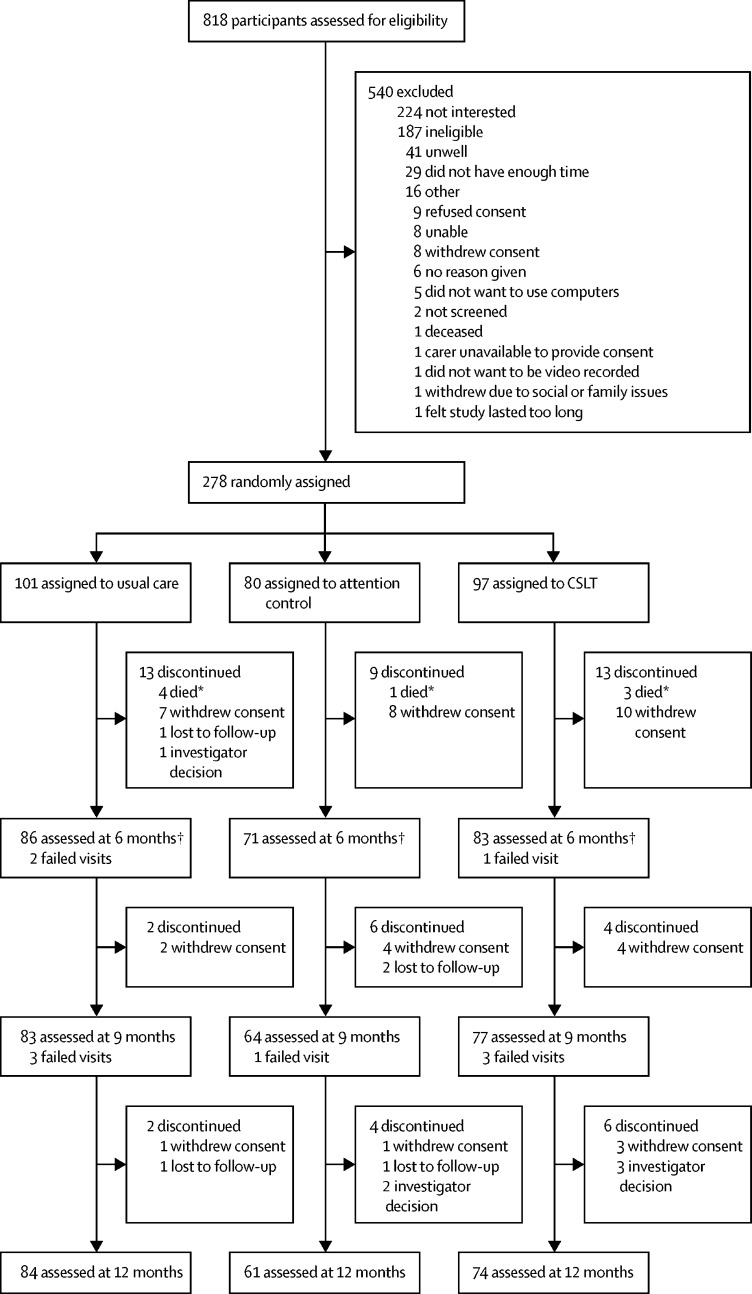
Trial profile CSLT=computerised speech language therapy. *Deaths before 6 months excluded in all clinical effectiveness analysis but included in safety analysis. † Participants included in the modified intention-to-treat primary analysis set.

Participants were recruited to the trial between Oct 20, 2014, and Aug 18, 2016, and were followed up between Oct 24, 2015, and Sept 12, 2017. A total of 818 participants were screened for eligibility, of whom 278 (34%) were randomly assigned to the usual care group (101 [36%]), the CSLT group (97 [35%]), and the attention control group (80 [29%]). Eight participants died before 6 months follow-up, 25 withdrew consent, one was lost to follow-up, and one was excluded based on investigators’ decisions. Reasons for dropout included personal and family issues, unhappiness with allocated study group, and unwillingness to complete outcome measures. In addition, outcome visits were not done with three participants. For further details see appendix (p 18). 240 (86%) of 278 participants were included as eligible in the mITT analysis: 86 (36%) of 240 in the usual care group, 83 (35%) of 240 in the CSLT group, 71 (30%) of 240 in the attention control group *(* figure 1).

The mean age of all participants was 65·4 years (SD 12·9) with a range 23 to 92 years and 164 (61%) of 270 were men. 119 (44%) of 270 participants had mild word finding difficulties (CAT Naming Objects test score 31–43 points out of 48), 80 (30%) were moderate (18–30 points), and 71 (26%) were severe (5–17 points). Participants were a median of 2 years (IQR 11 months to 4 years) post-stroke (range four months to 36 years). Intervention groups were broadly similar at baseline for both the mITT and multiple imputation analysis populations (table 2; appendix p 99). Notable exceptions include a greater proportion of participants with severe comprehension difficulties but a lower proportion of participants with severe word finding difficulties in the attention control group than the CSLT or usual care groups. The baseline TOMs scores were also slightly lower in the attention control group than the other two groups.

**Table 2:** Baseline characteristics of modified intention-to-treat analysis population

	**Usual care (n=86)**	**CSLT (n=83)**	**Attention control (n=71)**
**Sex**			
Male	54 (63%)	47 (57%)	44 (62%)
Female	32 (37%)	36 (43%)	27 (38%)
**Age at consent, years**			
Mean (SD)	64·9 (13·0)	64·9 (13·0)	63·8 (13·1)
Median (IQR)	66·5 (55·1–74·3)	64·7 (54·5–74·7)	65·1 (53·0–73·4)
Range	23·1–89·6	34·1–89·2	30·4–88·7
CAT comprehension severity*			
Within normal limits	17 (20%)	17 (21%)	13 (18%)
Mild	46 (54%)	35 (42%)	31 (44%)
Moderate	20 (23%)	26 (31%)	24 (34%)
Severe	3 (3%)	5 (6%)	3 (4%)
**Severity of word finding difficulty**†			
Mild	35 (41%)	36 (44%)	35 (49%)
Moderate	29 (34%)	26 (31%)	17 (24%)
Severe	22 (25%)	21 (25%)	19 (27%)
**Type of aphasia**			
Anomic	33 (38%)	33 (40%)	19 (27%)
Non-fluent (eg, Broca's Aphasia)	36 (42%)	34 (41%)	27 (38%)
Mixed non-fluent	13 (15%)	11 (13%)	20 (28%)
Fluent (eg, Wernicke's Aphasia)	4 (5%)	5 (6%)	5 (7%)
**Type of stroke**‡			
Infarct	69 (80%)	60 (72%)	58 (82%)
Haemorrhage	12 (14%)	13 (16%)	6 (8%)
Not known	9 (10%)	10 (12%)	7 (10%)
**Time post-stroke, years**			
Mean (SD)	2·8 (2·6)	2·9 (2·9)	3·6 (4·8)
Median (IQR)	1·9 (0·9–4·0)	1·9 (0·7–3·6)	2·1 (1·0–4·5)
Range	0·3–15·7	0·4–12·7	0·4–36·1
**Word finding ability, %**§			
Mean (SD)	42·6 (18·1)	43·7 (19·0)	41·7 (20·6)
Median (IQR)	42·3 (30·0–57·0)	43·0 (30·0–58·2)	37·5 (25·0–59·0)
Range	5·0–85·0	4·5–86·0	9·5–82·0
**Functional conversation, TOMs**¶			
Mean (SD)	3·1 (1·0)	2·9 (1·2)	2·7 (1·1)
Median (IQR)	3·0 (2·5–4·0)	3·0 (2·0–4·0)	2·5 (2·0–3·5)
Range	0·5–5·0	0·5–5·0	1·0–4·5
COAST, %**			
Mean (SD)	59·8 (13·2)	58·4 (13·6)	59·5 (14·0)
Median (IQR)	61·3 (51·9–68·8)	57·5 (47·5–68·8)	60·0 (48·8–67·5)
Range	26·3–86·3	26·3–87·5	26·3–96·3

Data are n (%), unless otherwise specified. CAT=Comprehensive Aphasia Test. COAST=Communication Outcomes After Stroke. CSLT=computerised speech language therapy. TOMs=Therapy Outcomes Measures.

* Derived from CAT comprehension of sentences test scores out of a total of 32 (within normal limits 27–32; mild 18–26, moderate 9–17, severe 0–8).

† Derived from CAT Naming objects test out of 48 (mild 31–43; moderate 18–30; severe 5–15).

‡ Some patients had several strokes, so summaries relate to patients with a particular type of stroke.

§ Word finding ability of personally chosen words based on the Personal Vocabulary Naming Test.

¶ TOMs rating score ranges from 0–5, with higher scores meaning improved functional communication.

** Higher score indicates positive self-perceived communication and positive impact on patient's quality of life.

**Figure 2:**
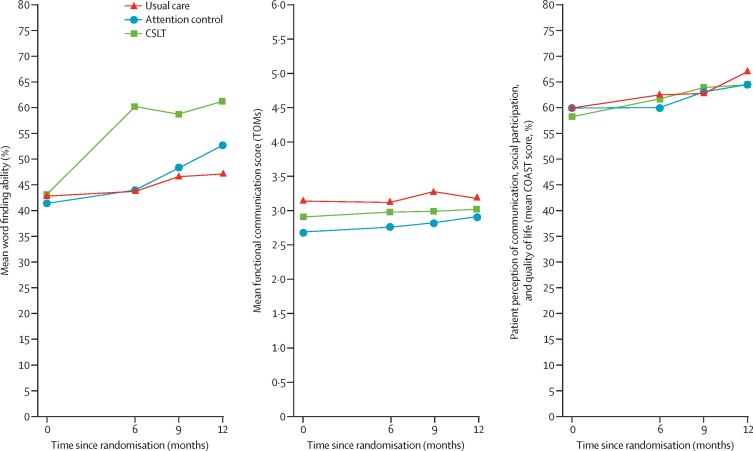
Mean response profile of participants for the co-primary and key secondary outcomes COAST=Communication Outcomes After Stroke. CSLT=computerised speech language therapy. TOMs=Therapy Outcome Measures.

For the first co-primary outcome, on average participants in the CSLT group had improved word finding of 16·2% more than those in the usual care group (95% CI 12·7–19·6; p<0·0001) and 14·4% more than those in the attention control group (10·8–18·1; table 3). The effect was in excess of the prespecified, minimal, clinically important difference of 10%. Improvement was maintained at 9 and 12 months (figure 2). Per-protocol results, which were consistent with the mITT results, are reported in the appendix (pp 44–47, 94–97).

**Table 3:** Co-primary and key secondary outcome results at 6 months in the modified intention-to-treat analysis population

	**Usual care**	**CSLT**	**Attention control**	CSLT *vs* usual care*		**CSLT *vs* attention control**†		**Attention control *vs* usual care**†	
				Adjusted mean difference in change (95% CI)	p value	Adjusted mean difference in change (95% CI)	p value	Adjusted mean difference in change (95% CI)	p value
**Co-primary outcomes**									
Change in word finding, %‡	86; 1·1 (11·2)	83; 16·4 (15·3)	71; 2·4 (8·8)	16·2 (12·7 to 19·6)^a^	<0·0001	14·4 (10·8 to 18·1)^c^	<0·0001	1·8 (−1·9 to 5·4)	0·338
Change in functional communication, TOMs§	84; 0·05 (0·59)	81; 0·04 (0·58)	68; 0·10 (0·61)	−0·03 (−0·21 to 0·14)^b^	0·709	−0·01 (−0·20 to 0·18)^d^	0·915	−0·02 (−0·21 to 0·17)	0·812
**Key secondary outcome**									
Change in participant's perception of communication, social participation, and quality of life, % (COAST)¶	83; 2·7 (12·6)	82; 3·3 (11·3)	68; −0·3 (12·7)	0·5 (−3·1 to 4·1)^e^	0·772	3·8 (−0·0 to 7·5)^f^	0·051	−3·2 (−7·0 to 0·5)	0·089

Data are n; mean (SD), unless otherwise specified.

a,b,c,d,e,f Referenced in the appendix (p 16) are the related p values to aid interpretation of the Hochberg procedure for decision-making to claim evidence. COAST=Communications Outcomes After Stroke. CSLT=computerised speech language therapy. TOMs=Therapy Outcome Measures.

* Usual care as the reference group.

† Attention control as the reference group.

‡ Higher scores indicate improved vocabulary of personal importance.

§ Higher scores indicate improved functional communication ability in conversation. Seven participants had missing TOMs data (video not recorded in error, poor sound quality of video, technical issues with the camera, participant declined to do a video, recording failed because of a technical issue, participant unwell and did not want to complete the assessment, and participant did not wish to complete assessment).

¶ Higher percentage indicates improved participant perception of communication effectiveness and a positive impact on their quality of life. Seven participants had invalid COAST records, with more than 10% of applicable items that were unclear or had no response (see statistical analysis plan p 16–17).

Mean changes in functional communication in conversation based on TOMs (the second co-primary outcome) were very similar across interventions (table 3; figure 2). The mean difference in change between the usual care group and the CSLT group was –0·03 (95% CI –0·21 to 0·14; p=0·709) and between the CSLT group and attention control group was –0·01 (–0·20 to 0·18). Therefore, the CSLT approach did not appear to improve functional communication in conversation.

The mean improvement in COAST in the CSLT group compared with the usual care group and the attention control group was not sufficient to suggest that CSLT improved the key secondary outcome (participant's own perception of their communication and social participation or its effect on their life; table 3). The results were similar after adjusting for additional covariates: length of time post-stroke and location of stroke (see appendix p 39). Location of stroke was reported as recorded in medical notes, hence a mixture of vascular territories and lobes were reported. For other secondary outcomes, on average, no improvement was seen in the number of treated words used in conversation across groups between baseline and 6 months (appendix pp 76–78). Additionally, evidence was not sufficient to suggest that improved word finding of treated words generalised to untreated words (appendix p 72). At 6 months, the mean change in word finding of untreated words was 3·9 (SD 7·9) in the usual care group, 3·3 (7·0) in the CSLT group, and 0·7 (8·5) in the attention control group, indicating an adjusted mean difference in change of –0·3 (95% CI –2·7 to 2·1) in favour of usual care compared with CSLT. Carer-related outcomes (ie, change in carer's perception of patient's communication and social participation, as assessed using the first 15 items of the CarerCOAST, and change in the carer QoL, as assessed using the last five items of the CarerCOAST) are reported in the appendix (pp 81, 100).

With regard to safety (appendix p 93), negative effects of CSLT were low. 23 (27%) of 85 participants in the CSLT group reported fatigue or anxiety at some point during the 6 months of computer use, which translates to one event per person per year. The number of participants who experienced any adverse events was 70 (61%) in the usual care group, 61 (72%) in the CSLT group, and 50 (63%) in the attention control group (table 4). These included tiredness, fits or seizures, visual difficulties, and headaches (summarised list in table 4 and table 5; appendix p 91). The number of participants who experienced any serious adverse events was 18 (16%) in the usual care group, nine (11%) in the CSLT group, and 11 (14%) in the attention control group (table 5). Examples of serious adverse events included admission to hospital due to urinary tract or chest infections. Differences in the incidences of adverse events and serious adverse events between groups were insufficient to suggest differences in risk levels.

**Table 4:** Incidences of adverse events in the safety analysis population

			**Usual care (n=114)**	**CSLT (n=85)**	**Attention control (n=79)**	CSLT *vs* usual care (IRR [95%CI])*	**CSLT *vs* attention control (IRR [95%CI])**†	Attention control *vs* usual care (IRR [95%CI])*
Experienced at least one adverse event			70 (61%)	61 (72%)	50 (63%)	..	..	..
Repeated adverse events								
	All adverse events							
		Total events per person years	200 per 105·4	185 per 84·7	136 per 74·7	..	..	..
		Incidence per person-year	1·87 (1·47–2·38)	2·18 (1·72–2·77)	1·79 (1·38–2·31)	1·16 (0·83–1·62)	1·22 (0·85–1·77)	0·95 (0·67–1·35)
	Felt more tired than usual							
		Total events per person years	125 per 105·4	114 per 84·7	77 per 74·7	..	..	..
		Incidence per person-year	1·18 (0·82–1·70)	1·32 (0·95–1·84)	1·01 (0·70–1·45)	1·12 (0·69–1·83)	1·32 (0·81–2·14)	0·85 (0·51–1·42)
	Had any fits or seizures							
		Total events per person years	18 per 105·4	47 per 84·7	13 per 74·7	..	..	..
		Incidence per person-year	0·16 (0·06–0·44)	0·57 (0·29–1·12)	0·17 (0·08–0·37)	3·48 (1·05–11·57)	3·41 (1·21–9·62)	1·02 (0·29–3·63)
	Had worsening vision or visual difficulties							
		Total events per person years	47 per 105·4	71 per 84·7	34 per 74·7	..	..	..
		Incidence per person-year	0·42 (0·22–0·80)	0·83 (0·51–1·36)	0·44 (0·25–0·79)	1·95 (0·87–4·37)	1·89 (0·89–4·05)	1·03 (0·43–2·44)
	Had increasing number or increasing severity of headaches							
		Total events per person years	46 per 105·4	52 per 84·7	25 per 74·7	..	..	..
		Incidence per person-year	0·43 (0·23–0·81)	0·58 (0·34– 1·01)	0·31 (0·13–0·78)	1·36 (0·59–3·11)	1·84 (0·64–5·30)	0·74 (0·24–2·21)
	Had any accidents (e.g. falls) or injuries							
		Total events per person years	90 per 105·4	48 per 84·7	51 per 74·7	..	..	..
		Incidence per person-year	0·87 (0·58–1·30)	0·56 (0·35– 0·89)	0·66 (0·42–1·04)	0·64 (0·35–1·19)	0·85 (0·45–1·61)	0·76 (0·42–1·39)
	Reported any other negative effects or events							
		Total events per person years	64 per 105·4	44 per 84·7	29 per 74·7	..	..	..
		Incidence per person-year	0·60 (0·40–0·92)	0·55 (0·35–0·86)	0·38 (0·21–0·68)	0·91 (0·49–1·68)	1·44 (0·69–3·00)	0·63 (0·31–1·28)

Data are n (%), total events per person years, or incidence per person-year (95% CI). Safety analysis using a negative binomial regression model was based on treatment as received; therefore, the numbers per group differ from the number randomised to each group (these numbers therefore differ from those in table 3). IRR=incidence rate ratio. CSLT=computerised speech language therapy.

* Usual care as the reference group.

† Attention control as the reference group.

**Table 5:** Incidences of serious adverse events in the safety analysis population

			**Usual care (n=114)**	**CSLT (n=85)**	**Attention control (n=79)**	CSLT *vs* usual care (IRR [95%CI])*	**CSLT *vs* attention control (IRR [95%CI])**†	Attention control *vs* usual care (IRR [95%CI])*
Experienced at least one serious adverse event			18 (16%)	9 (11%)	11 (14%)	..	..	..
	Repeated serious adverse events							
		Total events per person years	23 per 105·4	10 per 84·7	12 per 74·7	..	..	..
		Incidence per person-year	0·23 (0·11–0·34)	0·11 (0·04–0·19)	0·16 (0·06–0·26)	0·51 (0·22–1·19)	0·72 (0·28–1·87)	0·70 (0·31–1·59)
	Resulted in hospitalisation							
		Yes	19	10	11	..	..	..
		No	4	0	1	..	..	..
	Was life-threatening							
		Yes	9	3	4	..	..	..
		No	14	7	8	..	..	..
	Relationship to trial activity							
		Unlikely	1	2	2	..	..	..
		Unrelated	22	8	10	..	..	..
	Outcome of serious adverse events							
		Death‡	5	2	1	..	..	..

Data are n, n (%), total events per person years, or incidence per person-year (95% CI). Safety analysis using a negative binomial regression model was based on treatment as received; therefore, the numbers per group differ from the number randomised to each group (these numbers therefore differ from table 3). CSLT=computerised speech language therapy. IRR=incidence rate ratio.

* Usual care as the reference group.

† Attention control as the reference group.

‡ Not treatment-related deaths.

Predefined subgroup analyses indicated that the effect of CSLT on word finding was broadly consistent regardless of time post-stroke and slightly higher for participants with mild word finding difficulties and for those whose verbal comprehension was within normal limits (see appendix p 49–51). For functional conversation and perception of communication effectiveness or QoL, the subgroup and main results were consistent (see appendix pp 49, 50, 52, 53).

All participants randomly assigned to the CSLT group received the StepByStep aphasia therapy software on a computer. Some practice was recorded for all but 11 participants, with a mean of 28 h (SD 25·6) and a median of 21 h (IQR 4·9–49·7) in total between baseline and 6 months. In comparison, a mean amount of 3·8 h (SD 7·4) of usual speech and language therapy was received by all participants between baseline and 6 months. 57 (61%) of 94 participants in the CSLT group continued to use the computer exercises unsupported beyond the 6-month timepoint. Fidelity to CSLT, attention control and usual care is described in the appendix (p 106). In terms of quality of CSLT delivery, of particular note is that volunteers and therapy assistants only spent a median of 45 min per participant (IQR 22·0–77·5) over the 6 months practising newly learned words in conversation. More in depth analysis of fidelity of CSLT in Big CACTUS is in progress.

Regarding health economic evaluation, the per-patient incremental cost of CSLT was ·733 (95% credible interval 674 to 798) and the incremental QALY gain was 0·017 (–0·05 to 0·10), resulting in an ICER of ·42 686 per QALY gained compared with usual care. For CSLT compared with attention control, the ICER was ·40 164 per QALY gained. For CSLT compared with usual care, participants with mild word finding difficulty had an ICER of ·22 371 per QALY gained and for those with moderate word finding difficulty, the ICER was ·28 819 per QALY gained, whereas CSLT was more expensive and produced fewer QALYs than usual care for participants with severe word finding difficulties.

## Discussion

Big CACTUS shows that CSLT alongside usual care enables increased amounts of therapy practice and significantly improves the ability to retrieve personally relevant words chosen for practice. This outcome is likely to be due to the CSLT rather than the extra activity of the participant or attention provided by the therapy assistants. Improvement in word finding was maintained 6 months after the intervention period. However, CSLT did not have an effect on conversation, self-perceived improvements in everyday communication, social participation, or QoL.

It is notable that 57 (61%) of 94 participants chose to continue using the computer program unsupported beyond the end of the formal intervention period (if they still had access to it), suggesting many participants valued the opportunity to continue practising language skills independently. Subgroup analysis showed no effect of time post-stroke (ranged from 4 months to 36 years in this trial) on the ability to improve word finding, showing that people can continue to learn new words even a long time after the event. This finding supports the findings from small studies included in the Cochrane review of aphasia therapy,^2^ that SLT in general benefits patients with chronic aphasia.

Big CACTUS supports indications from smaller studies that improvements in the ability to find treated words in patients with chronic aphasia do not generalise to untreated words.^26^ Therefore, it is crucial that words of personal relevance form the focus of speech and language therapy in general to ensure usefulness. However, generalisation of newly learned words into conversation was not seen in the present trial, mirroring the findings in motor rehabilitation after stroke, where in the context of randomised comparisons with usual care or attention control interventions, improvements have been shown in motor function, balance, and gait velocity but not in functionally relevant activities of daily living.^27^ The findings of the present study therefore suggest that identifying ways to support functional use of new words is a priority to achieve the goal of improving conversation. The CSLT intervention intended that therapy assistants or volunteers would practise tasks to promote the use of new words in context. However, these tasks were only carried out for an average of 45 min in total over 6 months. Future studies will need to establish how to ensure support for practising words in context and whether increased practice in context helps with generalisation.

CSLT can be considered a safe intervention as only a few negative effects were reported by participants and the groups had no significant differences in incidences of adverse events or severe adverse events. Participants in the CSLT group spent, on average, 28 h on independent, repetitive word finding practice, at a mean cost of ·733 per participant. In contrast, 28 h of a therapist would cost approximately ·1400 per patient. Hence, relative to providing face-to-face speech and language therapy, CSLT is a low-cost option for delivering additional word finding therapy to patients with chronic aphasia post-stroke. Perhaps unsurprisingly, given that CSLT was not observed to affect conversation, the QALY gain associated with CSLT compared with usual care was low and the intervention is unlikely to be considered cost-effective for the whole patient population with aphasia because of the size of the incremental cost-effectiveness ratio. However, CSLT is more likely to be cost-effective than usual care for patients with mild and moderate word-finding difficulties. The key cost driver of delivering CSLT is time spent setting up the software and providing technical support, which in our study was done by therapists. These tasks could conceivably be done by therapy assistants or volunteers, substantially reducing costs. A full discussion of the health economic results will be the subject of a further publication.

Strengths of the study include recruitment and treatment of participants who are representative of the clinical population from a wide range of clinical settings that used existing models of IT services to implement CSLT. The CSLT intervention also has international applicability and is manualised and publicly available, enabling replication. Additional study strengths include the addition of a third group to enable differentiation of the effect of speech and language therapy components of CSLT from effects of additional activity and attention, and observation of fidelity to the trial interventions. Outcome measures addressed impairment, activity, and participation dimensions of the WHO International Classification of Functioning, Disability, and Health (ICF).^28^ Measuring across the ICF domains is important as this study suggests that word finding improvement (impairment) is not a good surrogate measure for functional conversation (activity). The trial also included health economic evaluation, and to our knowledge, our study is the first to do this for SLT. The use of an accessible version of the EQ-5D-5L permitted patients with aphasia to rate their own QoL for QALY estimation, rather than relying only on the view of a carer, which is not always the same as that of the patient themselves.^20^

A limitation is that only one computer program was used of the many commercial programs and apps available, and for only one language domain (ie, word finding) affected by aphasia. The results of the study also need to be considered in context of rapid technological advancement. Because the software used was only in English, patients who required treatment in a language other than English were excluded. Additionally, participant ethnicity was not recorded. While the intervention was given to all eligible participants, further research should identify patient characteristics that indicate motivation to participate in CSLT and likelihood of a good outcome for prudent targeting of the intervention, such as aphasia severity, age, and education. Participant characteristics influencing practice will be addressed in a further paper. Although data has been presented here on the amount of practice carried out, dose response and effect of continued practice are important. Preliminary data can be found in the appendix (p 59) and will be explored in more detail in further publications.

Despite blocking of the allocation sequence, slightly more participants were randomly assigned to CSLT and usual care groups compared with the attention control group. Sites recruiting small numbers of participants and the termination of recruitment after 278 patients might have contributed to this chance imbalance, which resulted in higher power than expected for the CSLT and usual care primary comparison and slightly lower power than expected for the CSLT and attention control comparison—although adequate to address the intended research objectives. We used the activity scale of the TOMs to measure conversations because it has good reliability and is likely to detect clinically meaningful change, though it might not detect small changes.^29^ Although cost-effectiveness was an important aspect of the study, it was not powered to detect a meaningful difference in QALYs. Finally, it was not possible to measure the amount of activity participants carried out with puzzle books in the attention control group with as much accuracy as the amount of computer practice carried out and therefore it is not known how well the attention control intervention controlled for CSLT.

In conclusion, this study has shown that CSLT is a low-cost intervention enabling access to additional word finding therapy for patients with chronic aphasia, leading to significant improvement in the ability to find words of personal relevance. Further research should focus on therapy techniques to promote generalisation of new words to functional, everyday communication contexts.

## Data sharing

Requests for patient-level data and statistical code should be made to the corresponding author and will be considered by members of the original trial management group, including the chief investigator and members of CTRU, who will release data on a case by case basis. Data will be shared following the principles for sharing patient-level data as described by Tudur Smith et al (*BMC Medicine* 2015; **13:** 298). The data will not contain any direct identifiers, we will minimise indirect identifiers, and remove free text data to minimise the risk of identification.

For the **intervention manual** see https://www.sheffield.ac.uk/polopoly_fs/1.525339!/file/TherapyManual_Nov15.pdf
