# Supplementary material for: Self-managed, computerised speech and language therapy for patients with chronic aphasia post-stroke compared with usual care or attention control (Big CACTUS): a multicentre, single-blinded, randomised controlled trial
Source: Lancet Neurol. 2019 Sep;18(9):821–33. doi: 10.1016/S1474-4422(19)30192-9 (PMC6700375; doi:10.1016/S1474-4422(19)30192-9)
Supplement: Supplementary appendix [file mmc1.pdf]

THE LANCET
Neurology

Supplementary appendix

This appendix formed part of the original submission and has been peer reviewed. We post it as supplied by the authors.

Supplement to: Palmer R, Dimairo M, Cooper C, et al. Self-managed, computerised speech and language therapy for patients with chronic aphasia post-stroke compared with usual care or attention control (Big CACTUS): a multicentre, single-blinded, randomised controlled trial. *Lancet Neurol* 2019; **18:** 821–33.

Appendix for:

Self-managed, computerised speech and language therapy for patients with chronic aphasia post-stroke compared to usual care or attention control (Big CACTUS): a multi-centre, single-blinded, randomised trial

Authors for statistical report: Munyaradzi Dimairo
Authors for short report on randomisation allocation: Mike Bradburn, Steven Julius

Author affiliation:

Clinical Trials Research Unit (CTRU), School of Health and Related Research (ScHARR), The University of Sheffield, Sheffield, England, UK (Munyaradzi Dimairo PhD, Mike Bradburn MSc, Prof Steven Julius PhD)

Correspondence to:

Prof. Cindy Cooper
Clinical Trials Research Unit (CTRU), School of Health and Related Research (ScHARR), The University of Sheffield, Western Bank, Sheffield. S10 2TN. England, UK

c.l.cooper@sheffield.ac.uk

Table of Contents

1 Statistical Report Version 1·0 9
1.1 Trial design and clinical effectiveness objectives 9
1.1.1 Trial design and governance 9
1.1.2 Primary clinical effectiveness objectives 9
1.1.3 Secondary clinical effectiveness objectives 9
1.2 Outcome measures 9
1.2.1 Co-primary endpoints 9
1.2.2 Key secondary endpoints 9
1.2.3 Other secondary endpoints 9
1.2.4 Safety secondary endpoints 10
1.2.5 Study outcomes 10
1.3 Sample size justification 11
1.4 Randomisation and concealment 11
1.5 Blinding aspects 11
1.6 Analysis populations 12
1.6.1 Modified intention-to-treat (mITT) set 12
1.6.2 Complete Case (CC) set 12
1.6.3 Per protocol (PP) sets 12
1.6.4 Multiple imputation (MI) set 12
1.6.5 Linear Interpolation (LI) set 13
1.6.6 Safety set 13
1.7 Statistical considerations 13
1.7.1 Dealing with deaths prior to six months and missing outcome data 13
1.7.2 Computation of summary outcome measures for analysis 13
1.8 Statistical analysis methods 15
1.8.1 Clinical effectiveness 15
1.8.2 Hochberg multiple hypothesis testing procedure for the co-primary and key secondary endpoints 16
1.8.3 Subgroup evaluation: co-primary and key secondary outcomes at six months 17
1.8.4 AEs and SAEs 17
1.8.5 Negative effects of computer use in the CSLT 17
1.9 Results 17
1.9.1 Participant flow and study discontinuation 17
1.9.2 Characteristics and demographics of randomised participants 20
1.9.3 Description of the usual care recorded three months pre-assessments 26
1.9.4 Computer use and adherence to the CSLT intervention 29
1.9.5 Puzzle books and adherence to the AC intervention 32
1.9.6 Unblinding of outcome assessments 33
1.9.7 Participants response profile for the primary and key secondary endpoints over time 33

1.9.8 Effect of the intervention on the co-primary and key secondary endpoints 38
1.9.9 Computer usage and association with change in outcomes at six months 59
1.9.10 Time post-stroke association with outcomes at 6 months 61
1.9.11 Association between age and outcomes at 6 months 63
1.9.12 Continued computer use and association with change in outcomes at six months 65
1.9.13 Puzzle books sent/contacts made and association with change in outcomes at six months 69
1.9.14 Intervention effect on generalisation to untreated words 70
1.9.15 Intervention effect on generalisation of treated words used in conversation 76
1.9.16 Carer rated communication effectiveness and impact on carer's quality of life 81
1.9.17 COAST and CaCOAST subitems 84
1.9.18 Safety results 87
1.9.19 Post hoc PP analysis of the co-primary and key secondary outcomes 93
1.10 Discussion 97
1.10.1 Main findings 97
1.10.2 Strengths and limitations 98
2 Baseline characteristics of multiple imputation population 99
3 Carer outcomes 100
4 Procedures to maintain blinding of outcome assessors 100
5 Short report on randomisation allocation 101
5.1 Background 101
5.2 Details of the randomisation 101
5.3 Implications of this for power in each arm 104
5.4 Should we have used minimisation? 104
6 Description of the pragmatic design according to the PRECIS (Pragmatic-explanatory continuum indicator summary) domains 105
7 Fidelity to CSLT, AC and UC interventions 106
7.1 Fidelity to the CSLT intervention (method) 106
7.2 Fidelity to the AC and UC interventions (results) 106
8 References 107

List of Figures

Figure 1: Interpretation of the Hochberg hierarchical sequential hypotheses testing strategy 16
Figure 2: Study participant flowchart 19
Figure 3: Distribution of the total usual care SLT time spent by the type of SLT 27
Figure 4: Total duration of usual care SLT received 3 months pre-assessment by the type of SLT 27
Figure 5: Mean duration of total usual care SLT received by the type of SLT (all participants) 28
Figure 6: Mean duration of total usual care SLT given by the type of SLT (only SLT receivers) 28
Figure 7: Relationship between the overall SLT received and time post-stroke 29
Figure 8: Delay in time to access computer therapy 30
Figure 9: Computer therapy practice time per participant over time 30
Figure 10: Distribution of computer use within 6 months 31
Figure 11: The distribution of computer practice sessions within 6 months 31
Figure 12: Distribution of puzzle books sent and successful contacts made within six months 33
Figure 13: Changes in word finding over time stratified by the intervention 34
Figure 14: Computer practice time of CSLT participants who fail to improve word finding 35
Figure 15: Changes in word finding over time stratified by the intervention and severity of word finding 35
Figure 16: Changes in conversation over time stratified by the intervention 36
Figure 17: Changes in conversation over time stratified by intervention and severity of word finding 36
Figure 18: Changes in COAST over time stratified by intervention 37
Figure 19: Changes in COAST over time stratified by intervention and severity of word finding 37
Figure 20: Mean word finding ability over time stratified by the intervention 40
Figure 21: Mean change word finding ability over time stratified by the intervention 40
Figure 22: Mean functional communication over time stratified by the intervention 41
Figure 23: Mean change in functional communication over time stratified by the intervention 41
Figure 24: Mean COAST over time stratified by the intervention 42
Figure 25: Mean change in COAST over time stratified by the intervention 43
Figure 26: Impact of attrition and intervention adherence on word finding at six months 44
Figure 27: Impact of attrition and intervention adherence on functional communication at six months 45
Figure 28: Impact of attrition and intervention adherence on COAST at six months 46
Figure 29: Subgroup influence on word finding results at six months 51
Figure 30: Subgroup influence on functional communication results at six months 52
Figure 31: Subgroup influence on COAST results at six months 53
Figure 32: Long-term intervention effect on word finding of treated words 56
Figure 33: Long-term intervention effect on functional communication 57
Figure 34: Long-term intervention effect COAST 58
Figure 35: Association between computer use and word finding stratified by severity of word finding 59
Figure 36: Association between computer use and functional communication stratified by severity of word finding 60
Figure 37: Association between computer use and COAST stratified by severity of word finding 60
Figure 38: Relationship between time post-stroke and word finding stratified by severity of word finding 61

Figure 39: Relationship between time post-stroke and functional communication stratified by severity of word finding 62
Figure 40: Relationship between time post-stroke and COAST stratified by severity of word finding 62
Figure 41: Relationship between age and word finding stratified by severity of word finding 63
Figure 42: Relationship between age and functional communication stratified by severity of word finding 64
Figure 43: Relationship between age and COAST stratified by severity of word finding 64
Figure 44: Changes in word finding over time stratified by continued computer use beyond six months 65
Figure 45: Average response in word finding over time stratified by continued computer use beyond six months 66
Figure 46: Changes in conversation over time stratified by continued computer use beyond six months 66
Figure 47: Average response in conversation over time stratified by continued computer use beyond six months 67
Figure 48: Changes in COAST over time stratified by continued computer use beyond six months 68
Figure 49: Average response in COAST over time stratified by continued computer use beyond six months 68
Figure 50: Relationship between the numbers of puzzle books sent and key outcomes at six months 69
Figure 51: Relationship between the numbers of contacts made and key outcomes at six months 70
Figure 52: Mean response profile in word finding of untreated words over time 71
Figure 53: Distribution of the numbers of treated words retrieved in conversation over time 76
Figure 54: Mean response profile in word finding of treated words over time 77
Figure 55: Distribution of change in treated words used in conversation over time 79
Figure 56: Association between change in treated words used in conversation and the number of contacts made 79
Figure 57: Average change in the carer rated communication effectiveness over time 82
Figure 58: Average change in the carer's quality of life over time 82
Figure 59: Impact of intervention adherence on word finding at 6 months (sensitivity analysis) 95
Figure 60: Impact of intervention adherence on functional communication at 6 months (sensitivity analysis) 96
Figure 61: Impact of intervention adherence on COAST at 6 months (sensitivity analysis) 97
Figure 62: Illustrating how pragmatic vs. explanatory the Big CACTUS trial was using the PRECIS-2 domains and scoring scale.[24] 105

List of Tables

Table 1: Outcomes, assessments/outcome measures and assessment time points 10

Table 2: Reasons for attrition 18

Table 3: Baseline demographic and characteristics of randomised participants 21

Table 4: Baseline characteristics of randomised participants (outcome covariates) 24

Table 5: Participants who received usual care SLT during the trial 26

Table 6: Summary of the distribution of computer practice time (N=94) 32

Table 7: Reasons for no record of computer therapy use at all (n=11) 32

Table 8: Cases of unblinding of outcome assessments 33

Table 9: Co-primary and key secondary outcomes at six months (mITT) 39

Table 10: Sensitivity analysis on the co-primary and key secondary outcomes at six months (mITT) 39

Table 11: Impact of adherence on the intervention effect on co-primary and key secondary endpoints 47

Table 12: Impact of attrition on the intervention effect on co-primary and key secondary outcomes at six months 48

Table 13. Subgroup analysis results: co-primary and key secondary outcome at six months 49

Table 14: Long-term intervention effect on the co-primary and key secondary outcomes at nine and 12 months 55

Table 15: Data completeness in word finding of untreated words 70

Table 16: Intervention effect on generalisation to untreated words at 6, 9 and 12 months 72

Table 17: Clinical improvement in generalisation to untreated words 73

Table 18: Clinical improvement in word finding of treated and untreated words (complete data) 74

Table 19: Clinical improvement in word finding of treated and untreated words (worst-case scenario) 75

Table 20: Intervention effect on generalisation of treated words used in conversation 78

Table 21: Clinical improvement in generalisation of treated words used in conversation 80

Table 22: Clinical improvement in word finding of treated words and treated words used in conversation 81

Table 23: Intervention effect on the carer rated communication effectiveness and carer's quality of life 83

Table 24: Intervention effect on the subitems of the COAST at 6, 9, and 12 months 85

Table 25. Intervention effect on the subitems of the CaCOAST at 6, 9, and 12 months 86

Table 26: Incidences of AEs (treatment-as-received) 88

Table 27: Incidences of AEs (treatment-as-randomised) 89

Table 28: Incidences of SAEs (treatment as received) 91

Table 29: Incidences of SAEs (treatment-as-randomised) 92

Table 30: Negative effect of computer therapy (only those who used the computer) 93

Table 31: Negative effect of the computer therapy (treatment as randomised) 93

Table 32: Post hoc analysis - impact of adherence on the intervention effect on co-primary and key secondary endpoints 94

Table 33. Baseline characteristics of multiple imputation analysis population (N=270) 99

Table 34: Allocation by site 102

Table 35: Allocation by severity 102

Table 36: Detailed allocation by site and strata 102

Table 37: Possible allocations in a block of size three 104
Table 38: Description of how pragmatic vs. explanatory the Big CACTUS trial was using the PRECIS-2 domains and scoring scale[24] 105
Table 39: Overall fidelity to the CSLT intervention 106

List of abbreviations used

AC	Attention control
ACT NoW	Assessing Communication Therapy in the North West
AE	Adverse event
ANCOVA	Analysis of Covariance
CaCOAST	Carer Communication Outcomes After Stroke
CAT	Comprehensive Aphasia Test
CC	Complete case
CI	Confidence interval
COAST	Communication Outcomes After Stroke
CSLT	Computerised speech and language therapy
CTRU	Clinical Trials Research Unit
EQ-5D	EuroQol health utility questionnaire
GCP	Good Clinical Practice
HTA	Health Technology Assessment
ICH	International Conference on Harmonisation
ICW	Information carrying words
IQR	Interquartile range
IR	Incidence rate
IRR	Incidence rate ratio
ITT	Intention-to-treat
MCA	Middle cerebral artery
MDC	Mean difference in change
MLE	Maximum likelihood estimate
MI	Multiple imputation
MICE	Multiple imputation using chained equations
mITT	Modified Intention-to-Treat
MVI	Mean value imputation
NHS	National Health Service
NIHR	National Institute for Health Research
OR	Odds ratio
PI	Principal Investigator
PP	Per-protocol
SAE	Serious adverse event
SD	Standard deviation
SLT	Speech and language therapy/therapist
TOMS	Therapy Outcome Measures
UC	Usual care
VAS	Visual analogue scale

1 Statistical Report Version 1·0

1.1 Trial design and clinical effectiveness objectives

1.1.1 Trial design and governance

The study is a pragmatic, three-arm, parallel group, single-blind, superiority, individually randomised controlled trial (RCT) which compared outcomes for people with persistent aphasia using computerised speech and language therapy (CSLT) with usual care at home with those having usual care (UC), or attention control (AC) with usual care. Participants were randomised to receive one of the three interventions using a 1:1:1 allocation ratio. The trial is funded by the National Institute for Health Research (NIHR) Health Technology Assessment (HTA) programme (Ref: HTA 12/21/01). The trial registration number is ISRCTN68798818.

1.1.2 Primary clinical effectiveness objectives

- To establish whether self-managed CSLT for word finding increases the ability of people with aphasia to use vocabulary of personal importance,
- To establish whether self-managed CSLT for word finding improves functional communication ability in conversation,
- To investigate whether patients receiving self-managed CSLT perceive greater changes in social participation in daily activities and quality of life,
- To identify whether any effects of the interventions are evident 12 months after therapy has begun.

1.1.3 Secondary clinical effectiveness objectives

- To investigate the generalisation of treatment to finding of untreated words;
- To investigate the carer perception of communication effectiveness and the impact on the carer quality of life;
- To identify any possible adverse events.

1.2 Outcome measures

The trial objectives were evaluated using the following endpoints and for CSLT compared to UC or AC. In addition to baseline measures, all outcomes were assessed at six (end of treatment), nine, and 12 months by speech language therapists (SLTs) at each centre that were blinded to treatment allocation.

1.2.1 Co-primary endpoints

- Change in word finding ability of words personally relevant to the participant at six months from baseline. Word finding ability was measured by a picture naming task using percentage scores based on a set of 100 personally selected words (see Section 1.1.7.2.2);
- Change in functional communication at six months from baseline measured by blinded ratings of video-recorded conversations between a speech language therapist (SLT) and participants, using the activity scale of the Therapy Outcome Measures (TOMS).

1.2.2 Key secondary endpoints

- Change in patient perception of communication and quality life at six months. This was assessed using the Communication Outcomes After Stroke (COAST) – a patient-reported measure of communication-related activity, participation, and quality of life validated for evaluating SLT interventions in the HTA Assessing Communication Therapy in the North West (ACT NoW) project.[1]

1.2.3 Other secondary endpoints

Additional secondary endpoints measured at 6 months are:

- Use of learnt vocabulary in the context of conversation at 6 months measured using a checklist of target words during rating of the videoed conversations.
- Generalisation to untreated words measured using the naming test from the Comprehensive Aphasia Test (CAT);

- Carer perception of communication effectiveness measured using the first 15 questions from the Carer COAST;
- Impact on carers' quality of life measured using the last five questions of the Carer COAST.

All primary and secondary outcomes were also assessed at nine and 12 months post-randomisation to identify any longer-term effect of the interventions.

1.2.4 Safety secondary endpoints

- Negative effects of treatment; patient diary were used to record any difficulties/negative impacts of the intervention every month for the 6-month treatment period,
- Serious adverse events/adverse events (SAEs/AEs) recorded at any time during the study period with formal checks carried out by SLTs every three months.

1.2.5 Study outcomes

Table 1: Outcomes, assessments/outcome measures and assessment time points

Outcome type	Outcome	Assessment/outcome measure	Assessor	Assessment visit time point	Treatment group
		CAT comprehension test	Blinded therapist at site	Baseline	CSLT, UC, AC
Co-primary	Change in ability to retrieve vocabulary of personal importance (impairment)	Naming pictures of 100 words of personal relevance for use in therapy	Blinded therapist at site	Baseline, **6 months (primary endpoint)**, 9 and 12 months	CSLT, UC, AC
Co-primary	Change in functional communication ability in conversation (activity) (general ability to convey information)	Activity scale of the Therapy Outcome Measures (TOMs) used to rate 10 minute videoed conversations, structured around topics of personal importance	Blinded therapist centrally	Baseline, **6 months (primary end point)**, 9 and 12 months	CSLT, UC, AC
Key secondary	Change in self-perception of communication, social participation and quality of life (participation)	Communication Outcomes After Stroke (COAST)	Self-reported with help from blinded therapist at site	Baseline, **6 months (primary end point)**, 9 and 12 months	CSLT, UC, AC
Secondary	Generalisation to untreated words	Comprehensive Aphasia Test (CAT) Naming Objects	Blinded therapist at site	Baseline, 6, 9, 12 months	CSLT, UC, AC
Secondary	Change in number of treated words used in videoed conversations (use of specific words practised)	Number of treated words used in 10-minute videos of conversations, structured around topics of personal importance	Blinded member of research team centrally	Baseline, 6, 9, 12 months	CSLT, UC, AC
Secondary	*Change in carers perception of patient participant' s communication, social participation, and carers quality of life	Carer Communication Outcomes After Stroke (COAST)	Carer self-report	Baseline, 6, 9, 12 months	CSLT, UC, AC
Patient safety	Adverse events (AEs) and serious adverse events (SAEs)	Information requested by Therapist at site	Self-reported	Baseline, 3, 6, 9, 12 months	CSLT, UC, AC
Patient safety	Negative effects of CSLT	Patient diary to record any difficulties or negative impacts of CSLT	Self-reported	1,2,3,4,5,6 months (postal)	CSLT
Health economic	Health-related quality of life (HRQoL) and resource use	Accessible variant of the EQ-5D-5L and when a carer was available, the standard EQ-5D-5L (proxy version 2) was also completed on behalf of participants	Self-reported with support from Blinded therapist at site	Baseline, 6, 9, 12 months	CSLT, UC, AC
	Carer health-related quality of life	EQ-5D-5L and CarerQol	Carer self-report	Baseline, 6, 9, 12 months	CSLT, UC, AC

Note: CSLT, Computer Speech and Language Therapy; AC, activity/attention control; UC, usual care

1.3 Sample size justification

The study aimed to recruit a maximum of 285 participants (95 per arm) across 20-24 SLT sites to address both co-primary endpoints (word finding of personally selected words and functional communication) for 90% power and a 5% two-sided significance level adjusted for a 15% dropout rate observed in an external pilot trial.[2] For the change in word finding, we assumed a 10% mean difference as clinically worthwhile to detect and a standard deviation (SD) of 17·38% estimated from an external pilot trial[2] based on an ANCOVA model. The sample size was inflated by 1·14 to account for the fact that the variance was estimated from a pilot trial.[3, 4] For the change in functional communication (TOMS activity scale), we sought an effect size of 0·45 of the SD as clinically worthwhile and a 0·5 correlation between baseline and outcome observed in the ACT NoW study, personally communicated by Prof Andy Vail, University of Manchester.

For the change in COAST, a key secondary endpoint, we sought 7·2% clinically worthwhile effect to detect, an SD of 18% based on externally supplied data and assumed a 0·5 correlation between baseline and outcome. For a sample size of 285 (95 per arm), the trial had 83% power for the COAST. The observed overall dropout rate was about 9% versus the planned 15%, as a result, further recruitment was terminated at 278 because the trial had the desired statistical power to address co-primary and key-secondary objectives.

1.4 Randomisation and concealment

We used a centralised web-based randomisation system hosted by the Sheffield CTRU to randomise participants to one of the three trial arms using a fixed 1:1:1 allocation ratio. The randomisation schedule was generated using stratified block randomisation with randomly ordered blocks of sizes three and six, stratified by centre and baseline severity of word finding score of the CAT Naming Test; mild (31 to 43), moderate (18 to 30), severe (5 to 17). Only the randomisation statistician knew about the block sizes and were disclosed after the trial had finished. A Sheffield CTRU statistician independent of the trial logged on to the randomisation system to specify the randomisation details and generated the randomisation schedule which was retained within the system. The system offers restricted access such that research team members are granted access to particular functionalities depending on their roles in the trial.

The SLTs randomised participants in their homes with informed consent using the Sheffield CTRU web-based randomisation system and disclose their allocation. If internet connection was unavailable, the SLT phoned the research team at the Sheffield CTRU to perform the randomisation online and gave the allocation immediately over the phone to the SLT to disclose to the participant.

1.5 Blinding aspects

This trial was a single blind-study recognising that participants could not be blinded to their treatment allocation. The SLTs with no previous involvement in the conduct of the trial assessed outcomes. The SLTs were trained, via a webinar session run by the central team, to remain unaware of the allocation of the participants they were assessing. Principal Investigators (PIs) were asked not to disclose baseline case report forms, not openly discuss participants with colleagues in open plan offices, and to remind their participants not to discuss their activities on the trial with any other SLTs they may come into contact with, as it is ‘a secret’.

When outcome assessors contacted participants and conducted their assessments they were advised to remind participants that their activity on the trial is ‘a secret’. It is possible that during a conversation with the participant or carer, outcome assessors could become unblinded by the participant or their carer. If this occurred on the telephone, before the assessment took place, then the assessment was carried out by a different blinded assessor. If this occurred at the end of the visit, when the assessment is complete, then this was not classed as an unblinded assessment, as the actual assessment was carried out when the assessor was still blinded. In the event of unblinding of the SLT occurring in this manner, the next assessment was carried out by a different blinded assessor. All sites had a minimum of two trained SLTs assessors that were blinded to the outcome to allow for unblinding issues. The intention was that for the same assessor to carry-out all outcome assessments for consistency, but if unblinding has occurred then an alternative assessor will be used as blinded assessments will take priority over assessments by the same assessor. If treatment arm allocation was disclosed during an assessment, then the outcome assessor continued with the assessment but subsequently alerted the PI and complete an unblinding form. The unblinding form asked the assessor to record what they believed the participant's treatment allocation to be – a ‘the suspected allocation’. In some instances, the assessor would guess the treatment allocation incorrectly, so the central team reported as ‘suspected unblinding’ only. For example, the SLT may believe the treatment allocation to be UC but in fact, the participant is allocated to AC.

1.6 Analysis populations

1.6.1 Modified intention-to-treat (mITT) set

The mITT population includes all participants for whom consent was obtained, treatment allocated as per randomised list regardless of circumstances after randomisation, and had primary outcome data at the 6-months assessment. Unlike the strict ITT that includes all randomised participants, the mITT excludes participants without primary outcome data at six months.

1.6.2 Complete Case (CC) set

This includes participants with outcome data at a particular assessment. The set was used for subsidiary analysis of the co-primary and key secondary outcomes at nine and 12 months, other secondary outcomes at different assessments, and for plotting mean profile response of outcomes over time across interventions.

1.6.3 Per protocol (PP) sets

The goal here is to explore the intervention effectiveness among participants who adhered to key components of the intervention as intended. Therefore, PP set includes participants for whom key components of the intervention were adhered to, including achieving the minimum amount of practice recommended[5, 6] and having access to support up to and including their 6-month assessment. PP classification relating to adherence to the intervention was only done for the CSLTand AC.

Across all interventions, participants were excluded from the PP if outcome measures were assessed 14 days before or 31 days after the expected 6-month assessment or were randomised but failed to meet at least one inclusion criterion. Thus, the four PP sets in the CSLT and AC were matched as follows:

1) practised computer therapy for a minimum total of 26 hrs (CSLT) or were sent at least six puzzle books (AC) within six months of randomisation (*PP1 CSLT26 AC*);
2) practised computer therapy for a minimum total of ten hrs (CSLT) or were sent at least six puzzle books (AC) within six months of randomisation (*PP2 CSLT10 AC6*);
3) practiced computer therapy for a minimum total of 26 hrs (CSLT) or were sent at least six puzzle books (AC) and contacted for at least four times (if they wish) (AC and CSLT) within six months of randomisation (*PP3 CSLT26 AC6_4*);
4) practiced computer therapy for a minimum total of ten hrs (CSLT) or were sent at least six puzzle books (AC) and contacted for at least four times (if they wish) (AC and CSLT) within six months of randomisation (*PP4 CSLT10 AC6_4*).

1.6.4 Multiple imputation (MI) set

This is for sensitivity analysis and includes all randomised participants excluding deaths prior to six months. For few participants with follow up outcome data but missed related baseline data for some reason, the mean value of those with available baseline data was used to impute missing baseline data during analysis. We adopted a strategy to inform the MI model for missing data. First, potential predictors of outcomes independent of the intervention were clinically pre-specified in the SAP (version 1.2). Second, the characteristics of completers (those meeting mITT inclusion) and all randomised participants (excluding deaths) were descriptively compared to explore predictors of missing data. Third, we explored the association between baseline characteristics and outcomes of interest. Based on these exploratory results, the intervention group appeared to be a mild predictor of missing data. Therefore, it was implausible to assume the data were missing completely at random. Other measured predictors of missing were unclear. As a result, the following strategy for imputing missing data was adopted:

- Intervention group (UC, AC, or CSLT), age, gender (male or female), the presence of a carer (yes or no), severity of word finding (total score), and severity of comprehension ability (total score) and baseline outcome measure under consideration were mandatory covariates in all MI models;
- Co-primary and key secondary outcomes at baseline (covariates), six, nine and 12 months were included in all imputation models and;
- The longitudinal nature of the outcome under consideration was accounted for in the MI models using chained equations[7, 8] via Stata ***mi*** command.

The multiple imputation using chained equations (MICE) analysis was conducted and reported in accordance with the guidance provided by White et al. (2011).[9] The number of imputations (n=20) was chosen based on the observed proportion of missing data as recommended in the literature.[9]

1.6.5 Linear Interpolation (LI) set

For additional sensitivity analysis on the impact of missing data on the results, an LI model (deterministic imputation) was used. Where data were missing at an assessment t_i but valid data are available at previous (t_{i-1}) and future (t_{i+1}) assessments, the missing value was linearly interpolated by the formula:

$$y_{missing} = y_{i-1} + (y_{i+1} - y_{i-1})\frac{t_i - t_{i-1}}{t_{i+1} - t_{i-1}}.$$

1.6.6 Safety set

Safety analysis relates to the evaluation of the intervention effect on AEs and SAEs, and negative effects of computer therapy. This includes all randomised participants with informed consent and treatment allocation for analysis used the actual intervention received based on available evidence such as the number of books sent and computer therapy practice time. For sensitivity analysis, treatment allocation as randomised was used as well.

1.7 Statistical considerations

1.7.1 Dealing with deaths prior to six months and missing outcome data

Some deaths in this trial population during the trial were expected. The research team discussed implications of deaths and approaches to handle them during analysis. The influence of the trial interventions on increasing the risk of mortality was anonymously viewed very unlikely. In addition, the interpretation of imputed missing data, such as word finding and functional communication for participants who died was clinically challenging. As a result, the research team agreed against imputing missing data due to death. Therefore, deaths prior to six month assessment were excluded in any clinical effectiveness analysis but included in the safety analysis. This approach is consistent with related recommendations.[10] There were no deaths after the six month assessment.

We performed sensitivity analysis to explore the impact of missing data due to other reasons unrelated to death on the bias of the results using the MI, LI, and CC sets described in Section 1.1.6.

1.7.2 Computation of summary outcome measures for analysis

1.7.2.1 Categorisation of severity of word finding difficulty and comprehension ability

The severity of word finding difficulty was categorised based on total scores from the CAT Naming Test using a severity rating of mild (31-43), moderate (18-30), and severe (5-17). A categorical comprehension ability variable at baseline was generated based on total scores from the CAT Comprehension of Spoken Words classified as follows:

- severe (0 to 8); inconsistently understanding at two information carrying words (ICW) level,
- moderate (9 to 17); consistently understanding at 2-3 ICW level/simple sentence structures but not complex sentence structures,
- mild (18 to 26); some understanding of complex sentence structures but not consistent,
- within normal limits (27 to 32) based on CAT cut-off score for normal/aphasic.

1.7.2.2 Word finding of personally selected words for treatment

Personal Vocabulary Naming Test was used to assess word finding ability based on 100 personally selected words for treatment. For each personally selected word, word finding ability was then assessed using the scoring system: 0 for an incorrect or no response; 1 for a correct word named correctly after a delay of five seconds and/or for a self-correction; and 2 for a correct prompt answer within five seconds. This scoring system yields a potential maximum score of 200. Although all participants were expected to be assessed based on 100 personally selected words, it was possible that some participants could have been assessed based on less than 100 words for some reason such as tiredness. At least 70 words should have been assessed for an assessment to

be considered valid. If less than 100 words but more than 70 words were assessed, the word finding ability for participant k (Y_k), expressed as a percentage was calculated based on the total score relative to the potential maximum score as:

$$Y_k = \frac{\sum_{i=1}^{X} item_i}{2X} * 100;$$

where $i = \{1, 2, \ldots, X\}$ is the picture item considered for pesonal vocabulary naming and X is the total number of personally selected words assessed.

1.7.2.3 Functional communication

The activity dimension of the TOMS instrument was used to assess functional communication rating (conversation). The rating is measured on a six-point ordinal scale ranging from 0 (unable to communicate in any way) to 5 (communicates effectively in all situations) and allows scoring between ordinal descriptors such as 0·5, 1·5, 2·5, 3·5, and 4·5. Thus, the rating scale has 11 ordinal possibilities which can be treated on a continuous scale. There is a ceiling effect for participants who are able to communicate effectively in all situations with a TOMS rating of 5 at baseline. We report the numbers and proportions of these participants across interventions.

1.7.2.4 Self-perceived communication effectiveness and impact on quality of life

The COAST is a patient-centred measure used to assess self-perceived communication effectiveness and impact on quality of life for people with aphasia and/or dysarthria.[11] The measure has 20 items and each item is assessed on a rating scale of 0 to 4. Other responses (‘not applicable’, ‘unclear’ or ‘no responses’) are permitted. A procedure is then applied to compute a percentage score under a number of scenarios; all applicable and answered items, the existence of ‘not applicable’ items, the existence of ‘unclear’ or ‘no response’ items. We computed the overall percentage score using a validated algorithm as described by Bowen et al (2009).[12]

1.7.2.5 Carer perception of patient’s communication effectiveness and impact on their quality of life

The CaCOAST assesses carer perception of patient’s communication effectiveness and impact on their quality of life.[13] The measure has 20 items, each item is assessed on a scale of 0 to 4, and a percentage summary measure is calculated. The CaCOAST was administered by the research therapists as one questionnaire, however, the first 15 items and the last five items were analysed separately as they address two different research questions. The first 15 items assess carer perception of patient’s communication while the last five items assess the impact of the patient’s communication difficulties on the carer’s quality of life. Although the original scoring algorithm is based on all 20 items, we considered the first 15 items and the last five items separately to assess different aspects. We, therefore, modified the scoring algorithm consistent with the original scoring system using 20 items[12] to compute the $CaCOAST_{15}$ (%) and $CaCOAST_5$ (%) but based on the first 15 and last five items, respectively. This uses the same scoring algorithm as described in Section 1.1.7.2.4 but account for missing data as other aspects are uninformative (‘not applicable’ and ‘unclear’).

1.7.2.6 Generalisation of treated to untreated words

The CAT Naming Test consists of 24 (picture naming tasks) words and assesses generalisation of treated to untreated words. For each picture naming task, the following scoring system was used depending on a participant’s response: 0 for an incorrect response;1 for an accurate response after a delay of more than five seconds, and 2 for an accurate and prompt answer. A total score ranging from 0 to 48 was then generated to assess word finding of untreated words. Missing information (item level or all items) was possible due to tiredness or being unable to complete the tests. For missing items, summary measures from the CAT Naming Test and Comprehension of Spoken Words were calculated assuming conservative worst-case scenario – a zero for a missing item score. No summary measure was calculated if all items were missing.

1.7.2.7 Word finding of treated words used in conversation

The use of vocabulary in the context of the conversation was assessed using a checklist of target words during ratings of videoed conversations at six months. Out of the 100 treated words, personally selected for treatment, the number of words retrieved during videoed conversations was counted (total score ranging from 0 to 100). A correct word retrieved was counted only once regardless of the number of times it was retrieved during the conversation.

1.7.2.8 EQ5D utility and VAS

The EQ-5D-5L version was used to assess health status and produces a single index value for health status for use in the calculation of quality-adjusted life years to inform health economics evaluation of investigative interventions.[14] The instrument consists of an EQ-5D-5L descriptive system and an EQ-5D-5L VAS. The descriptive system has five dimensions assessing mobility, self-care, usual activity, pain/discomfort, and anxiety. Each of these dimensions has five levels of severity which participants were asked to select one of them to best describe their health status 'today': no problems, slight problems, moderate problems, severe problems, and extreme problems. Based on participants' responses from these 5 dimensions, a single index value was calculated as detailed by Devlin et al (2018).[15] The index values are on a scale of 1 (full health) to 0 (state equivalent to dead) and health states considered to be worse than dead attain negative values (<0).
As for the EQ-5D-5L VAS, participants were asked to rate how good or bad their health is 'today' on a scale of 0 (the worst health imaginable) to 100 (best health imaginable). The scores from this continuous scale assess change in overall self-rated health status. The described approaches were applied to the carer, patient-proxy and patients aphasia-friendly versions of the EQ-5D-5L.

1.8 Statistical analysis methods

A pre-specified statistical analysis plan (SAP) written by a blinded trial statistician in accordance with the ICH E9 principle[16] and signed off before unblinded review of the data guided the analysis of this study (SAP version 1.2). All post hoc analyses are declared. For this report, the study is reported in conformity with the CONSORT guidelines for individually randomised parallel group trials [17] and harms.[18]

1.8.1 Clinical effectiveness

For the co-primary and key secondary outcomes at six months, we used a multiple linear regression model adjusted for associated baseline outcome measures and fixed stratification factors; centre and severity of word finding (mild, moderate and severe). The maximum likelihood estimate (MLE) of the intervention effect was expressed as the adjusted mean difference in change (MDC) between the CSLT and UC, and the CSLT and AC, with associated 95% CI and P-value. The MLE of the intervention effect (with associated 95% CI) between the UC and AC obtained via contrasts was used for exploratory analysis. We used a Hochberg procedure described in Section 1.1.8.2 to control for false positive error rate for claiming effectiveness evidence. For sensitivity analysis, we used a multiple linear regression model adjusted for associated baseline outcome measures, fixed stratification factors (centre and severity of word finding), length of time post-stroke (continuous), and the location of stroke (yes or no); middle cerebral artery (MCA), frontal lobe, parietal lobe, and temporal lobe.

The long-term intervention effect at nine and 12 months on the co-primary and key secondary outcomes, and other secondary continuous outcomes (at six, nine, and 12 months) such as CaCOAST, word finding of untreated words and treated words used in conversation was evaluated using a multiple linear regression model adjusted for associated baseline outcome measures and fixed stratification factors; centre and severity of word finding (mild, moderate and severe).

A multiple logistic regression model adjusted for stratification factors (centre and severity of word finding) was used to explore the intervention effect on the proportion of participants achieving pre-defined clinical improvements of 5% and 10% in both word finding of treated words (from Personal Vocabulary Naming Test) and untreated words (from CAT Naming Test). The numbers and proportion of participants meeting each clinical improvement criterion are reported by intervention together with the Odd Ratios (OR) and associated 95% CI and P-value. This was performed under two scenarios by considering: only participants with complete data; and all randomised participants, but assuming that those with missing data failed to achieve clinical improvement (worst-case scenario).

The beneficial effects of the computer therapy on seven themes of the COAST and CaCOAST, each of the seven items (3, 4, 6, 11, 15, 16, 17, 20) were explored at six, nine and 12 months using a bootstrapping procedure [19] following graphical inspection. The median rating (IQR) by the intervention group and the median difference in rating with its 95% CI were estimated without statistical significance testing. Participants for whom item responses were recorded as 'not applicable', 'unclear' or 'no response' were excluded from the analysis of those specific items.

For post hoc analysis at the request of the CI following the disclosure of the results (as per predefined SAP), we

explored the intervention effect on the proportion of participants who did and did not use treated words in conversation based on a 5% or 10 % improvement in treated words (from Personal Vocabulary Naming Test) and used at least five or ten treated words (retrieved during videoed conversation). We calculated the proportion of participants meeting each clinical improvement criterion by intervention. The difference in proportions of participants achieving a 'clinical improvement' criterion between interventions was calculated with associated 95% CI estimated using the normal approximation to the binomial distribution without significance testing. A multiple logistic regression model adjusted for stratification factors (centre and severity of word finding) and baseline measures was used to explore the intervention effect on the proportion of participants achieving clinical improvement of 5% and 10% in the generalisation of word finding to untreated words (from CAT Naming Test) without significance testing.

1.8.2 Hochberg multiple hypothesis testing procedure for the co-primary and key secondary endpoints

We interpreted the co-primary (word finding and functional communication) and key secondary (COAST) outcomes results at six months using a Hochberg procedure to control the chances of falsely declaring statistically significant results (at 5% nominal level) due to multiple hypothesis testing;[20] multiple endpoints (co-primary and key secondary) and key multiple treatment comparisons (CSLT versus UC and CSLT versus AC). Figure 1 illustrates the interpretation strategy of the results in order to claim statistical significance and superiority of the intervention.

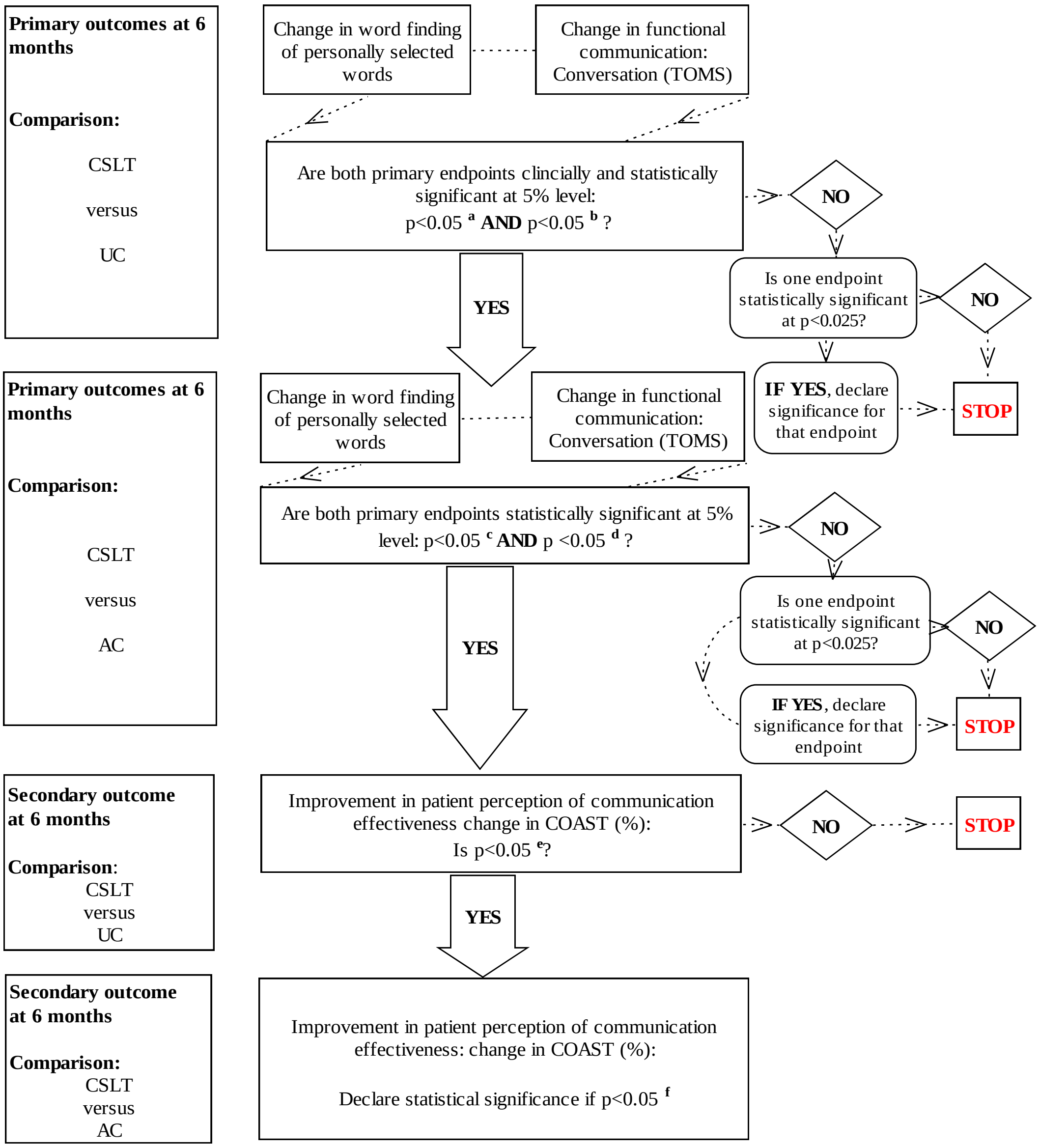

Figure 1: Interpretation of the Hochberg hierarchical sequential hypotheses testing strategy

Note: Superscripts [a, b, c, d, e, f] are referenced in Table 9

1.8.3 Subgroup evaluation: co-primary and key secondary outcomes at six months

The following subgroups were prespecified to explore potential heterogeneity in the intervention effect on the co-primary and key secondary outcomes at six months:

1) the severity of word finding difficulty: mild (31 to 43), moderate (18 to 30), and severe (5 to 17);
2) comprehension ability: within normal limits (27 to 32) mild (18 to 26), moderate (9 to 17), and severe (0 to 8);
3) the length of time post-stroke; categorisation was quantile-based since there was no existing literature to guide the clinical classification.

Subgroup analysis was performed based on the mITT set. The number of participants and mean change in outcomes are reported stratified by intervention received and subgroup category. We assessed effect modification between the intervention and subgroup using a multiple linear regression model that included an interaction term between the intervention and the subgroup of interest adjusted for baseline outcome measures and fixed stratification factors; centre and severity of word finding difficulty (mild, moderate, and severe). We report the overall P-values from the interaction tests to explore the strength of evidence for heterogeneity of the intervention effect across subgroups. Forest plots are used for presentation of results to aid visual interpretation showing the MLE of the intervention effect between the CSLT and UC, and CSLT and AC with associated 95% CIs stratified by subgroup category.

1.8.4 AEs and SAEs

The primary analysis includes all randomised participants based on treatment allocation as received as described in Section 1.1.6.6. Sensitivity analysis was performed using treatment allocation as randomised principle (strict ITT).

We calculated the number and proportion of participants who experienced any AEs or SAEs by intervention. For each participant, we calculated the exposure (study follow-up) and the number of repeated AEs and SAEs and estimated the incidence rate (IR) of AEs and SAEs by intervention. We used a negative binomial regression model to estimate the IR in each intervention and incidence rate ratio (IRR) with associated 95% CI accounting for overdispersion and the exposure without statistical significance testing.

1.8.5 Negative effects of computer use in the CSLT

The primary analysis includes all randomised participants based on treatment allocation as received as described in Section 1.1.6.6. Sensitivity analysis was performed using treatment allocation as randomised principle. The number and proportion of participants experienced any perceived negative effects are summarised stratified by negative effect category; tiredness, vision, headaches, and anxiousness or worrisome. We calculated the total number of repeated events experienced by a participant per negative effect category and exposure (follow-up contributing to the six months data). We used a negative binomial regression model accounting for overdispersion and the exposure without significance testing to estimate the IR with 95% CI.

1.9 Results

1.9.1 Participant flow and study discontinuation

We identified 995 participants eligible for screening across 21 UK SLT centres predominantly via patient records and support groups between September 2014 and August 2016. Of these, we obtained consent in 288 (28·9%) eligible participants and randomised 278 (27·9%): UC (n=101), AC (n=80), and CSLT (n=97). Figure 2 presents the flow of participants from screening to study completion (12 months from randomisation).

Of the 278 randomised participants, 8 (2·9%) died before their 6-month assessments: UC (n=4), AC (n=1), and CSLT (n=3). For the remaining 270, 240 (88·9%) completed their 6-month assessments. The proportions of participants who completed six and 9-month outcome assessments were very similar across interventions. However, the discontinuation rate at 12 months was slightly higher in the AC (23·8%) and the CSLT (23·7%) compared to the UC (16·8%). The reasons for attrition are given in Figure 2 and detailed in Table 2. The most common reasons are personal or family issues and being unhappy with the allocated study arm.

In total, 240 randomised participants were eligible for inclusion in the primary mITT analysis: 86 in the UC, 71 in the AC, and 83 in the CSLT. As for the MI analysis, 270 randomised participants were eligible for inclusion: UC (n=97), AC (n=79), and CSLT (n=94).

Table 2: Reasons for attrition

Discontinuation type	Reason for withdrawal	UC (n=17)	AC (n=19)	CSLT (n=23)	Total (N=59)
Death	NA	4	1	3	8
Investigator decision	Personal/family issue	1	2	3	6
Lost to follow-up	NA	2	3	0	5
Participant withdrew consent	Personal/family issue	5	4	6	15
	Unhappy with allocated study arm	4	3	4	11
	Unwilling to complete follow-up questionnaires	1	3	2	6
	Prefers not to say	0	1	1	2
	Moving out of the area	0	0	1	1
	Other (lost motivation and feeling unwell)	0	1	0	1
	Other (time commitment)	0	0	1	1
	Other [a]	0	0	1	1
	Other [b]	0	1	0	1
	Other [c]	0	0	1	1

Note: [a] participant struggled with voice recognition not working consistently, difficulties with the computer and frustration; [b] Participant wanted to spend their time doing other activities that they enjoy more and feel are more beneficial; [c] reported finds it upsetting if does not do well. “It's not doing me any good" despite explaining that it is just the monitoring now to help with the study and she has done all the hard work”; NA=Not applicable

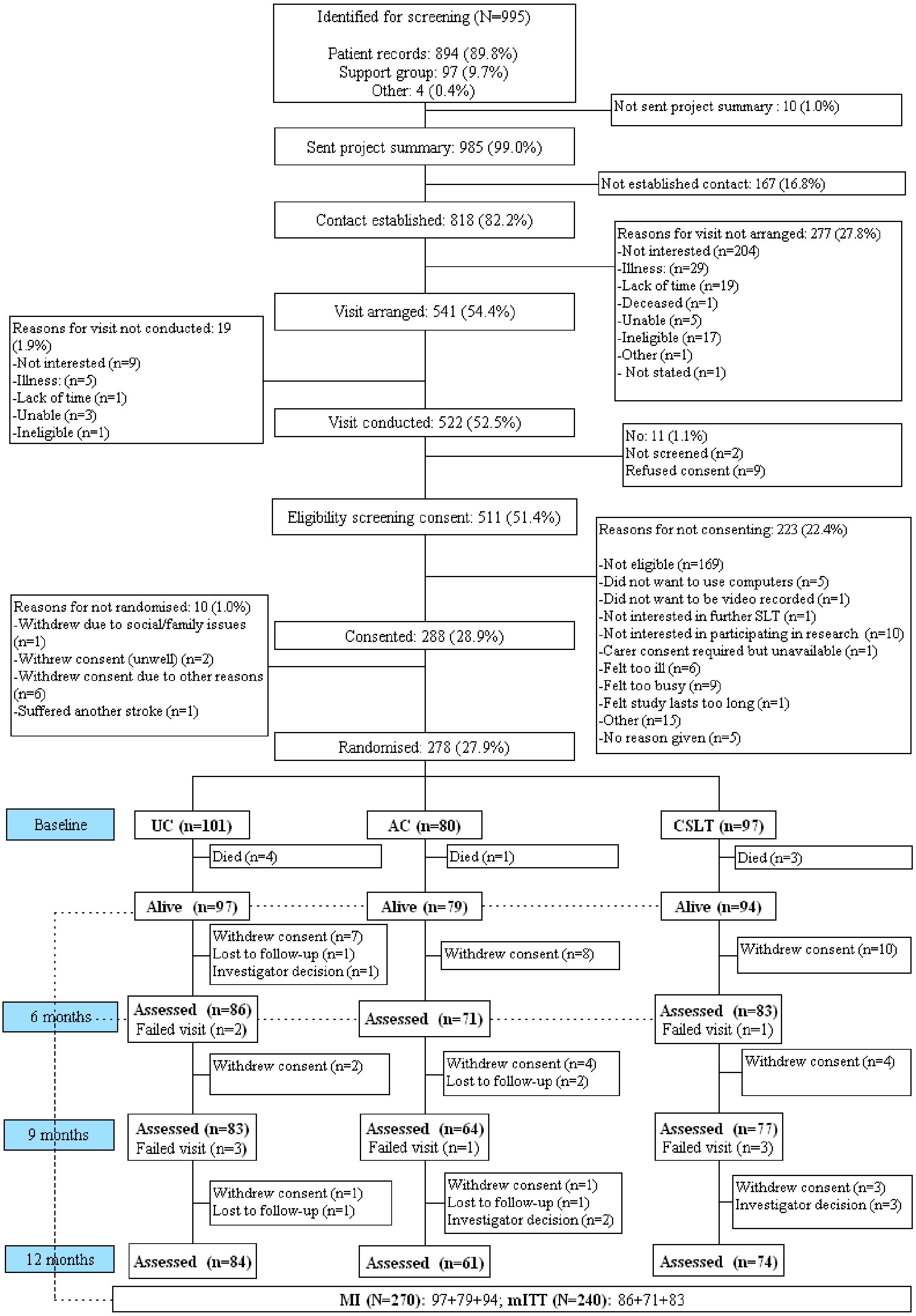

Figure 2: Study participant flowchart

Note: "Patient records (past or present)" included word of mouth from SLT colleagues
MI=multiple imputation population; mITT=modified intention to treat population

1.9.2 Characteristics and demographics of randomised participants

For the 270 participants eligible for inclusion in the clinical effectiveness analysis, recruitment across 21 UK SLT centres (20 NHS trusts) ranged from seven to 22 participants with a median (IQR) of 12 (10 to 15). The majority of participants 164 (60·7%) were male. The overall mean age at consent (SD) was 65·4 (12·9) years, ranging from 23·1 to 91·8 years. Most participants 119 (44·1%) had mild word finding difficulty compared to 80 (29·6%) moderate and 71 (26·3%) severe cases. The overall median time post-stroke (IQR) was approximately 2 (1 to 4) years. Most participants showed no evidence of apraxia of speech (64·4%) and had suffered an infarction stroke (78·5%).

The demographics and characteristics of randomised participants (those meeting MI inclusion; n=270) and completers (those meeting the mITT inclusion; n=240) are detailed in Table 3 and Table 4. In summary, randomised participants and completers were very similar on average (Table 3). In addition, on average, the participants appeared broadly similar across interventions. However, by chance, there were a few exceptions indicating relatively small differences between interventions such as the location of the stroke, the type of stroke and lateralisation, if not brain stem.

The characteristics of the randomised participants and completers (with their available carers) with respect to continuous covariates ('outcomes' assessed at baseline) are shown in Table 4, which are broadly similar between the two populations (MI and completers - mITT) and across interventions. However, the available carers of the UC participants who agreed to take part had slightly higher CaCOAST scores on average compared to their counterparts. It should also be noted that the participant was the unit of randomisation and not the supporting carer.

Table 3: Baseline demographic and characteristics of randomised participants

Variable	All (*excluding 8 deaths*)				Completers (*mITT*)			
	UC (n=97)	AC (n=79)	CSLT (n=94)	Total (N=270)	UC (n=86)	AC (n=71)	CSLT (n=83)	Total (N=240)
**Site**								
Ayr	3(3·1%)	2(2·5%)	4(4·3%)	9(3·3%)	3(3·5%)	2(2·8%)	4(4·8%)	9(3·8%)
Belfast	5(5·2%)	3(3·8%)	3(3·2%)	11(4·1%)	5(5·8%)	3(4·2%)	3(3·6%)	11(4·6%)
Cambridgeshire	5(5·2%)	3(3·8%)	5(5·3%)	13(4·8%)	5(5·8%)	3(4·2%)	4(4·8%)	12(5·0%)
Cwm Taf	3(3·1%)	1(1·3%)	5(5·3%)	9(3·3%)	2(2·3%)	1(1·4%)	4(4·8%)	7(2·9%)
Derbyshire	6(6·2%)	5(6·3%)	5(5·3%)	16(5·9%)	6(7·0%)	5(7·0%)	5(6·0%)	16(6·7%)
Dorset	4(4·1%)	3(3·8%)	5(5·3%)	12(4·4%)	4(4·7%)	3(4·2%)	5(6·0%)	12(5·0%)
Glasgow	8(8·2%)	6(7·6%)	8(8·5%)	22(8·1%)	8(9·3%)	5(7·0%)	7(8·4%)	20(8·3%)
Hull	6(6·2%)	4(5·1%)	5(5·3%)	15(5·6%)	5(5·8%)	4(5·6%)	4(4·8%)	13(5·4%)
Newcastle	6(6·2%)	5(6·3%)	4(4·3%)	15(5·6%)	6(7·0%)	4(5·6%)	4(4·8%)	14(5·8%)
Norfolk	3(3·1%)	5(6·3%)	2(2·1%)	10(3·7%)	2(2·3%)	4(5·6%)	2(2·4%)	8(3·3%)
North Bedford ‡	3(3·1%)	3(3·8%)	4(4·3%)	10(3·7%)	3(3·5%)	3(4·2%)	4(4·8%)	10(4·2%)
North Lincolnshire	4(4·1%)	5(6·3%)	2(2·1%)	11(4·1%)	3(3·5%)	3(4·2%)	2(2·4%)	8(3·3%)
Northampton	5(5·2%)	4(5·1%)	6(6·4%)	15(5·6%)	5(5·8%)	4(5·6%)	6(7·2%)	15(6·3%)
Northern	5(5·2%)	4(5·1%)	3(3·2%)	12(4·4%)	5(5·8%)	4(5·6%)	3(3·6%)	12(5·0%)
Nottinghamshire	6(6·2%)	6(7·6%)	7(7·4%)	19(7·0%)	5(5·8%)	6(8·5%)	7(8·4%)	18(7·5%)
Plymouth	3(3·1%)	1(1·3%)	3(3·2%)	7(2·6%)	3(3·5%)	1(1·4%)	2(2·4%)	6(2·5%)
Sheffield	5(5·2%)	4(5·1%)	6(6·4%)	15(5·6%)	5(5·8%)	4(5·6%)	6(7·2%)	15(6·3%)
Somerset	4(4·1%)	5(6·3%)	5(5·3%)	14(5·2%)	1(1·2%)	4(5·6%)	3(3·6%)	8(3·3%)
South Bedford ‡	6(6·2%)	5(6·3%)	4(4·3%)	15(5·6%)	5(5·8%)	4(5·6%)	2(2·4%)	11(4·6%)
Sunderland	3(3·1%)	2(2·5%)	5(5·3%)	10(3·7%)	2(2·3%)	2(2·8%)	4(4·8%)	8(3·3%)
Swansea	4(4·1%)	3(3·8%)	3(3·2%)	10(3·7%)	3(3·5%)	2(2·8%)	2(2·4%)	7(2·9%)
**Sex**								
Male	60(61·9%)	49(62·0%)	55(58·5%)	164(60·7%)	54(62·8%)	44(62·0%)	47(56·6%)	145(60·4%)
Female	37(38·1%)	30(38·0%)	39(41·5%)	106(39·3%)	32(37·2%)	27(38·0%)	36(43·4%)	95(39·6%)
**Age at consent (years)**	(n=97)	(n=79)	(n=94)	(n=270)	(n=86)	(n=71)	(n=83)	(n=240)
Mean(SD)	65·6(13·1)	64·8(13·1)	65·6(12·7)	65·4(12·9)	64·9(13·0)	63·8(13·1)	64·9(13·0)	64·6(13·0)
Median(IQR)	66·6(55·8, 74·7)	66·2(54·6, 74·9)	66·1(55·5, 75·5)	66·4(55·8, 74·9)	66·5(55·1, 74·3)	65·1(53·0, 73·4)	64·7(54·5, 74·7)	65·8(54·6, 74·2)

Variable	All (*excluding 8 deaths*)				Completers (*mITT*)			
	UC (n=97)	AC (n=79)	CSLT (n=94)	Total (N=270)	UC (n=86)	AC (n=71)	CSLT (n=83)	Total (N=240)
Min, Max	23·1, 91·8	30·4, 88·7	34·1, 89·2	23·1, 91·8	23·1, 89·6	30·4, 88·7	34·1, 89·2	23·1, 89·6
**CAT Comprehension score** [a]	(n=97)	(n=79)	(n=94)	(n=270)	(n=86)	(n=71)	(n=83)	(n=240)
Mean(SD)	21·0(6·0)	19·5(7·2)	20·0(7·0)	20·2(6·7)	21·0(5·9)	19·8(7·0)	20·1(7·3)	20·3(6·7)
Median(IQR)	22·0(17·0, 26·0)	21·0(14·0, 25·0)	21·5(15·0, 26·0)	22·0(15·0, 26·0)	22·0(17·0, 26·0)	21·0(14·0, 26·0)	22·0(14·0, 26·0)	22·0(15·0, 26·0)
Min, Max	0·0, 30·0	1·0, 30·0	0·0, 32·0	0·0, 32·0	0·0, 30·0	1·0, 30·0	0·0, 32·0	0·0, 32·0
**CAT comprehension severity** [a]								
Severe	3(3·1%)	6(7·6%)	5(5·3%)	14(5·2%)	3(3·5%)	3(4·2%)	5(6·0%)	11(4·6%)
Moderate	24(24·7%)	24(30·4%)	29(30·9%)	77(28·5%)	20(23·3%)	24(33·8%)	26(31·3%)	70(29·2%)
Mild	50(51·5%)	36(45·6%)	43(45·7%)	129(47·8%)	46(53·5%)	31(43·7%)	35(42·2%)	112(46·7%)
Within normal limits	20(20·6%)	13(16·5%)	17(18·1%)	50(18·5%)	17(19·8%)	13(18·3%)	17(20·5%)	47(19·6%)
**Severity of word finding difficulty** [b]								
Mild	40(41·2%)	38(48·1%)	41(43·6%)	119(44·1%)	35(40·7%)	35(49·3%)	36(43·4%)	106(44·2%)
Moderate	33(34·0%)	19(24·1%)	28(29·8%)	80(29·6%)	29(33·7%)	17(23·9%)	26(31·3%)	72(30·0%)
Severe	24(24·7%)	22(27·8%)	25(26·6%)	71(26·3%)	22(25·6%)	19(26·8%)	21(25·3%)	62(25·8%)
**Type of aphasia**								
Anomic	39(40·2%)	22(27·8%)	35(37·2%)	96(35·6%)	33(38·4%)	19(26·8%)	33(39·8%)	85(35·4%)
Non-fluent (e.g. Broca's)	40(41·2%)	29(36·7%)	38(40·4%)	107(39·6%)	36(41·9%)	27(38·0%)	34(41·0%)	97(40·4%)
Mixed non-fluent	13(13·4%)	21(26·6%)	15(16·0%)	49(18·1%)	13(15·1%)	20(28·2%)	11(13·3%)	44(18·3%)
Fluent (e.g. Wernicke's)	5(5·2%)	7(8·9%)	6(6·4%)	18(6·7%)	4(4·7%)	5(7·0%)	5(6·0%)	14(5·8%)
**Evidence of apraxia of speech**								
No	64(66·0%)	48(60·8%)	62(66·0%)	174(64·4%)	55(64·0%)	42(59·2%)	52(62·7%)	149(62·1%)
Yes	33(34·0%)	31(39·2%)	32(34·0%)	96(35·6%)	31(36·0%)	29(40·8%)	31(37·3%)	91(37·9%)
**Type of stroke**								
Infarct	79(81·4%)	64(81·0%)	69(73·4%)	212(78·5%)	69(80·2%)	58(81·7%)	60(72·3%)	187(77·9%)
Haemorrhage	14(14·4%)	7(8·9%)	14(14·9%)	35(13·0%)	12(14·0%)	6(8·5%)	13(15·7%)	31(12·9%)
Not known	9(9·3%)	8(10·1%)	11(11·7%)	28(10·4%)	9(10·5%)	7(9·9%)	10(12·0%)	26(10·8%)
**Location of stroke**								
Middle cerebral artery (MCA)	47(48·5%)	48(60·8%)	43(45·7%)	138(51·1%)	41(47·7%)	43(60·6%)	37(44·6%)	121(50·4%)
Frontal lobe	8(8·2%)	5(6·3%)	11(11·7%)	24(8·9%)	7(8·1%)	5(7·0%)	9(10·8%)	21(8·8%)

Variable	All (*excluding 8 deaths*) UC (n=97)	AC (n=79)	CSLT (n=94)	Total (N=270)	Completers (*mITT*) UC (n=86)	AC (n=71)	CSLT (n=83)	Total (N=240)
Temporal lobe	13(13·4%)	3(3·8%)	3(3·2%)	19(7·0%)	12(14·0%)	1(1·4%)	3(3·6%)	16(6·7%)
Parietal lobe	14(14·4%)	7(8·9%)	11(11·7%)	32(11·9%)	13(15·1%)	6(8·5%)	9(10·8%)	28(11·7%)
Occipital lobe	5(5·2%)	0(0·0%)	3(3·2%)	8(3·0%)	4(4·7%)	0(0·0%)	3(3·6%)	7(2·9%)
Cerebellum	1(1·0%)	0(0·0%)	0(0·0%)	1(0·4%)	1(1·2%)	0(0·0%)	0(0·0%)	1(0·4%)
Not known	25(25·8%)	23(29·1%)	33(35·1%)	81(30·0%)	22(25·6%)	21(29·6%)	31(37·3%)	74(30·8%)
**Lateralisation (if not brain stem)**								
Right side	9(9·3%)	1(1·3%)	5(5·3%)	15(5·6%)	9(10·5%)	1(1·4%)	5(6·0%)	15(6·3%)
Left side	79(81·4%)	73(92·4%)	81(86·2%)	233(86·3%)	69(80·2%)	65(91·5%)	71(85·5%)	205(85·4%)
Not known	12(12·4%)	5(6·3%)	11(11·7%)	28(10·4%)	11(12·8%)	5(7·0%)	10(12·0%)	26(10·8%)
**Time post stroke (years)**	(n=97)	(n=79)	(n=94)	(n=270)	(n=86)	(n=71)	(n=83)	(n=240)
Mean(SD)	2·8(2·7)	3·4(4·6)	2·8(2·9)	3·0(3·4)	2·8(2·6)	3·6(4·8)	2·9(2·9)	3·0(3·5)
Median(IQR)	1·9(0·9, 3·8)	1·9(1·0, 4·3)	1·8(0·7, 3·6)	1·9(0·9, 4·0)	1·9(0·9, 4·0)	2·1(1·0, 4·5)	1·9(0·7, 3·6)	1·9(0·9, 4·0)
Min, Max	0·3, 15·7	0·4, 36·1	0·4, 12·7	0·3, 36·1	0·3, 15·7	0·4, 36·1	0·4, 12·7	0·3, 36·1

Note: [a] Based on a CAT Comprehension of Spoken Sentences with possible total scores ranging from 0 to 32; [b] based on the CAT Naming Test with possible total scores ranging from 0 to 48; Min=minimum; Max=maximum; SD=standard deviation; IQR=interquartile range (25th percentile, 75th percentile); ‡ 2 sites in same NHS trust

Table 4: Baseline characteristics of randomised participants (outcome covariates)

Variable	All (*excluding 8 deaths*)				Completers (*mITT*)			
	**UC (n=97)**	**AC (n=79)**	**CSLT (n=94)**	**Total (N=270)**	**UC (n=86)**	**AC (n=71)**	**CSLT (n=83)**	**Total (N=240)**
**Word finding ability (%)** [a]	(n=97)	(n=79)	(n=94)	(n=270)	(n=86)	(n=71)	(n=83)	(n=240)
Mean(SD)	42·8(18·1)	41·4(20·7)	43·2(19·0)	42·6(19·1)	42·6(18·1)	41·7(20·6)	43·7(19·0)	42·7(19·1)
Median(IQR)	44·0(30·0, 57·0)	37·5(23·5, 59·0)	43·8(30·0, 57·5)	41·8(27·0, 57·5)	42·3(30·0, 57·0)	37·5(25·0, 59·0)	43·0(30·0, 58·2)	41·5(27·8, 57·5)
Min, Max	5·0, 85·0	9·0, 82·0	4·5, 86·0	4·5, 86·0	5·0, 85·0	9·5, 82·0	4·5, 86·0	4·5, 86·0
**Functional communication (TOMS)** [b]	(n=96)	(n=78)	(n=93)	(n=267)	(n=86)	(n=70)	(n=82)	(n=238)
Mean(SD)	3·1(1·0)	2·7(1·0)	2·9(1·2)	2·9(1·1)	3·1(1·0)	2·7(1·1)	2·9(1·2)	2·9(1·1)
Median(IQR)	3·0(2·5, 4·0)	2·5(2·0, 3·5)	3·0(2·0, 4·0)	3·0(2·0, 4·0)	3·0(2·5, 4·0)	2·5(2·0, 3·5)	3·0(2·0, 4·0)	3·0(2·0, 4·0)
Min, Max	0·5, 5·0	1·0, 4·5	0·5, 5·0	0·5, 5·0	0·5, 5·0	1·0, 4·5	0·5, 5·0	0·5, 5·0
**COAST (%)** [c]	(n=94)	(n=79)	(n=89)	(n=262)	(n=84)	(n=71)	(n=79)	(n=234)
Mean(SD)	59·9(13·1)	60·0(13·8)	58·2(13·6)	59·3(13·5)	59·8(13·2)	59·5(14·0)	58·4(13·6)	59·2(13·5)
Median(IQR)	61·3(52·5, 68·8)	60·0(48·8, 68·8)	57·5(48·8, 68·8)	60·0(50·0, 68·8)	61·3(51·9, 68·8)	60·0(48·8, 67·5)	57·5(47·5, 68·8)	60·0(48·8, 68·8)
Min, Max	26·3, 86·3	26·3, 96·3	26·3, 87·5	26·3, 96·3	26·3, 86·3	26·3, 96·3	26·3, 87·5	26·3, 96·3
**CaCOAST 15 (%)** [d]	(n=58)	(n=49)	(n=62)	(n=169)	(n=53)	(n=44)	(n=56)	(n=153)
Mean(SD)	56·8(14·9)	53·7(13·2)	52·8(15·6)	54·4(14·7)	56·5(14·7)	54·0(13·3)	53·6(14·7)	54·7(14·3)
Median(IQR)	57·5(46·7, 66·7)	51·7(43·3, 66·7)	50·8(41·7, 63·3)	55·0(43·3, 66·7)	58·3(46·7, 66·7)	52·5(43·3, 66·7)	50·8(42·5, 64·2)	55·0(43·3, 66·7)
Min, Max	26·7, 81·7	28·3, 78·3	18·3, 81·7	18·3, 81·7	26·7, 81·7	28·3, 78·3	20·0, 81·7	20·0, 81·7
**CaCOAST 5 (%)** [e]	(n=58)	(n=49)	(n=62)	(n=169)	(n=53)	(n=44)	(n=56)	(n=153)
Mean(SD)	54·7(19·3)	44·7(16·4)	48·2(21·0)	49·4(19·5)	54·1(18·5)	45·1(16·7)	48·7(20·5)	49·5(19·0)
Median(IQR)	55·0(40·0, 70·0)	50·0(30·0, 55·0)	47·5(30·0, 65·0)	50·0(35·0, 65·0)	55·0(40·0, 65·0)	50·0(30·0, 55·0)	50·0(32·5, 65·0)	50·0(35·0, 65·0)
Min, Max	20·0, 100·0	10·0, 95·0	5·0, 90·0	5·0, 100·0	20·0, 100·0	10·0, 95·0	5·0, 90·0	5·0, 100·0
**Word finding of untreated words** [f] **(CAT score)**	(n=97)	(n=79)	(n=94)	(n=270)	(n=86)	(n=71)	(n=83)	(n=240)
Mean(SD)	26·4(11·0)	26·2(11·5)	26·5(11·4)	26·4(11·2)	26·2(11·0)	26·6(11·3)	26·6(11·3)	26·4(11·1)
Median(IQR)	28·0(18·0, 36·0)	30·0(16·0, 36·0)	27·5(17·0, 38·0)	28·0(17·0, 36·0)	27·5(17·0, 35·0)	30·0(16·0, 37·0)	27·0(17·0, 38·0)	28·0(17·0, 36·0)
Min, Max	5·0, 43·0	6·0, 42·0	5·0, 43·0	5·0, 43·0	5·0, 43·0	6·0, 42·0	5·0, 43·0	5·0, 43·0
**EQ VAS score (patients-aphasia friendly)** [h]	(n=97)	(n=79)	(n=94)	(n=270)	(n=86)	(n=71)	(n=83)	(n=240)
Mean(SD)	69·8(17·6)	68·9(20·0)	67·0(21·1)	68·6(19·5)	70·0(17·5)	68·7(20·3)	67·0(21·5)	68·6(19·8)

Variable	All (*excluding 8 deaths*)				Completers (*mITT*)			
	UC (n=97)	AC (n=79)	CSLT (n=94)	Total (N=270)	UC (n=86)	AC (n=71)	CSLT (n=83)	Total (N=240)
Median(IQR)	75·0(55·0, 80·0)	75·0(55·0, 85·0)	70·0(50·0, 85·0)	70·0(51·0, 85·0)	75·0(60·0, 80·0)	70·0(55·0, 85·0)	70·0(50·0, 85·0)	70·0(53·0, 85·0)
Min, Max	30·0, 100·0	10·0, 100·0	10·0, 100·0	10·0, 100·0	30·0, 100·0	10·0, 100·0	10·0, 100·0	10·0, 100·0
**EQ-5D-5L index (patients-aphasia friendly)** [g]	(n=97)	(n=79)	(n=93)	(n=269)	(n=86)	(n=71)	(n=83)	(n=240)
Mean(SD)	0·72(0·20)	0·70(0·22)	0·70(0·22)	0·71(0·21)	0·75(0·17)	0·70(0·22)	0·70(0·22)	0·72(0·20)
Median(IQR)	0·75(0·61, 0·88)	0·75(0·57, 0·87)	0·75(0·61, 0·87)	0·75(0·61, 0·87)	0·76(0·66, 0·88)	0·75(0·57, 0·89)	0·75(0·61, 0·87)	0·75(0·61, 0·87)
Min, Max	0·21, 1·00	0·05, 1·00	0·02, 1·00	0·02, 1·00	0·21, 1·00	0·05, 1·00	0·02, 1·00	0·02, 1·00
**EQ-5D-5L Index (carer)** [g]	(n=59)	(n=49)	(n=63)	(n=171)	(n=53)	(n=44)	(n=57)	(n=154)
Mean(SD)	0·85(0·18)	0·83(0·18)	0·82(0·19)	0·83(0·18)	0·85(0·18)	0·83(0·19)	0·82(0·19)	0·83(0·19)
Median(IQR)	0·90(0·82, 0·95)	0·87(0·78, 0·92)	0·87(0·75, 0·92)	0·88(0·79, 0·94)	0·90(0·82, 0·95)	0·88(0·78, 0·92)	0·87(0·75, 0·92)	0·89(0·79, 0·93)
Min, Max	0·21, 1·00	0·19, 1·00	-0·00, 1·00	-0·00, 1·00	0·21, 1·00	0·19, 1·00	-0·00, 1·00	-0·00, 1·00
**EQ VAS score (carer)** [h]	(n=58)	(n=49)	(n=63)	(n=170)	(n=53)	(n=44)	(n=57)	(n=154)
Mean(SD)	79·5(15·0)	76·6(19·0)	76·0(18·8)	77·4(17·6)	79·2(15·3)	77·7(17·7)	75·4(19·2)	77·4(17·5)
Median(IQR)	80·0(75·0, 90·0)	80·0(65·0, 90·0)	80·0(65·0, 90·0)	80·0(70·0, 90·0)	80·0(75·0, 90·0)	80·0(70·0, 90·0)	80·0(65·0, 90·0)	80·0(70·0, 90·0)
Min, Max	25·0, 100·0	20·0, 98·0	25·0, 100·0	20·0, 100·0	25·0, 100·0	20·0, 98·0	25·0, 100·0	20·0, 100·0
**EQ VAS score (proxy)** [h]	(n=73)	(n=56)	(n=65)	(n=194)	(n=64)	(n=49)	(n=59)	(n=172)
Mean(SD)	62·5(18·9)	64·1(21·9)	62·0(21·6)	62·8(20·6)	62·6(18·9)	65·4(21·1)	62·0(21·6)	63·2(20·4)
Median(IQR)	65·0(50·0, 80·0)	70·0(50·0, 80·0)	60·0(45·0, 80·0)	65·0(50·0, 80·0)	65·0(50·0, 80·0)	70·0(55·0, 80·0)	60·0(45·0, 80·0)	66·0(50·0, 80·0)
Min, Max	15·0, 95·0	4·0, 95·0	10·0, 100·0	4·0, 100·0	15·0, 95·0	4·0, 95·0	10·0, 100·0	4·0, 100·0
**EQ-5D-5L Index (patient-proxy)** [g]	(n=73)	(n=56)	(n=64)	(n=193)	(n=64)	(n=49)	(n=58)	(n=171)
Mean(SD)	0·63(0·23)	0·64(0·21)	0·61(0·24)	0·63(0·23)	0·64(0·23)	0·65(0·22)	0·60(0·24)	0·63(0·23)
Median(IQR)	0·68(0·49, 0·78)	0·70(0·51, 0·77)	0·65(0·39, 0·79)	0·68(0·45, 0·78)	0·69(0·51, 0·78)	0·71(0·51, 0·78)	0·63(0·39, 0·79)	0·68(0·45, 0·78)
Min, Max	-0·11, 1·00	-0·06, 1·00	0·04, 1·00	-0·11, 1·00	-0·11, 1·00	-0·06, 1·00	0·04, 1·00	-0·11, 1·00

Note: [a] word finding ability of personally chosen words (%) based on the Personal Vocabulary Naming Test; [b] TOMS rating score ranges from 0 to 5, with higher scores meaning improved functional communication; [c] higher score indicates positive self-perceived communication and impact patient's quality of life; [d] Based on the first 15 items of the CaCOAST, with higher scores indicating positive carer's perception of patient's communication ability; [e] based on the last 5 items of the CaCOAST with higher scores indicating positive carer perception of the impact of the patient's communication ability on the carer's quality of life; [f] based on a CAT Naming Test with total scores ranging from 0 to 48 and higher scores indicate improved word finding ability of untreated words; [g] higher values indicate higher health-related quality of life; [g] higher score indicates positive perception of health status, with 0 and 100 meaning worst and best health status imaginable; Min=minimum; Max=maximum; SD=standard deviation; IQR=interquartile range (25th percentile, 75th percentile); -0·00 means <0.

1.9.3 Description of the usual care recorded three months pre-assessments

Table 5 presents the proportion of participants that received usual care SLT during the trial three months prior to an assessment stratified by the type of SLT they received across interventions. In general, the distribution of usual care SLT seems comparable across interventions; however, slightly fewer participants in the AC received usual care SLT compared to the UC and CSLT, especially three months prior to baseline. In addition, slightly more participants in the UC received the UC SLT compared to their counterparts. The proportions of participants receiving SLT decreased across groups as the trial progresses.

Table 5: Participants who received usual care SLT during the trial

Nature of usual care SLT	Timepoint	UC (n = 96) [a]	AC (n = 79)	CSLT (n = 94)
Overall SLT	Baseline	43(44·8%)	30(38·0%)	42(44·7%)
	3 months	33(34·4%)	22(27·8%)	30(31·9%)
	6 months	23(24·0%)	14(17·7%)	21(22·3%)
	9 months	17(17·7%)	12(15·2%)	12(12·8%)
	12 months	13(13·5%)	10(12·7%)	8(8·5%)
Rehabilitation SLT	Baseline	35(36·5%)	21(26·6%)	35(37·2%)
	3 months	23(24·0%)	16(20·3%)	17(18·1%)
	6 months	18(18·8%)	10(12·7%)	13(13·8%)
	9 months	11(11·5%)	7(8·9%)	8(8·5%)
	12 months	8(8·3%)	5(6·3%)	7(7·4%)
Enabling SLT	Baseline	30(31·3%)	23(29·1%)	25(26·6%)
	3 months	21(21·9%)	14(17·7%)	19(20·2%)
	6 months	10(10·4%)	8(10·1%)	12(12·8%)
	9 months	9(9·4%)	6(7·6%)	5(5·3%)
	12 months	6(6·3%)	5(6·3%)	4(4·3%)
Supportive SLT	Baseline	34(35·4%)	19(24·1%)	29(30·9%)
	3 months	25(26·0%)	17(21·5%)	22(23·4%)
	6 months	19(19·8%)	6(7·6%)	11(11·7%)
	9 months	10(10·4%)	9(11·4%)	6(6·4%)
	12 months	7(7·3%)	10(12·7%)	4(4·3%)

Note: [a] usual care SLT data were not collected for one participant.

The distribution of total time spent providing usual care SLT to each participant by intervention and nature of SLT given is displayed in Figure 3. Figure 4 shows the corresponding distribution of the total usual care SLT time summed up across participants within interventions.

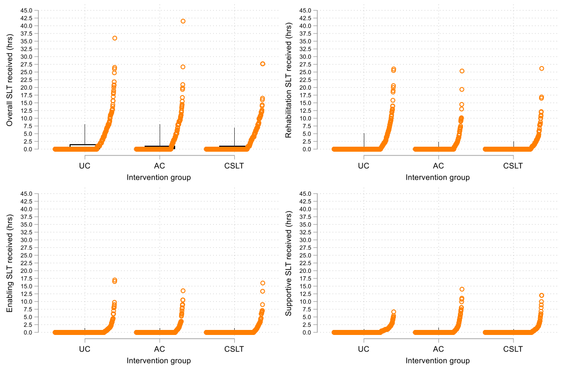

Figure 3: Distribution of the total usual care SLT time spent by the type of SLT

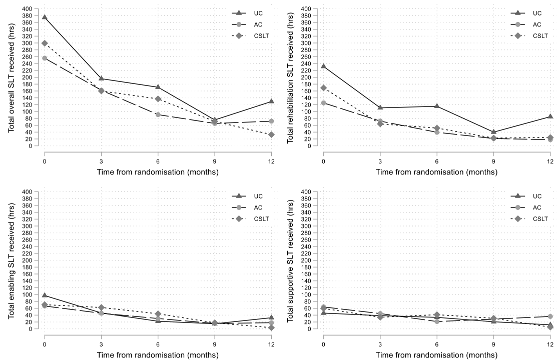

Figure 4: Total duration of usual care SLT received 3 months pre-assessment by the type of SLT

The distribution of the average duration of usual care SLT time spent per participant for each SLT treatment by intervention is shown in Figure 5. The averages here are calculated including all participants in the denominator regardless of whether they received usual care SLT or not. Figure 6 shows the corresponding figure only including participants who received usual care SLT.

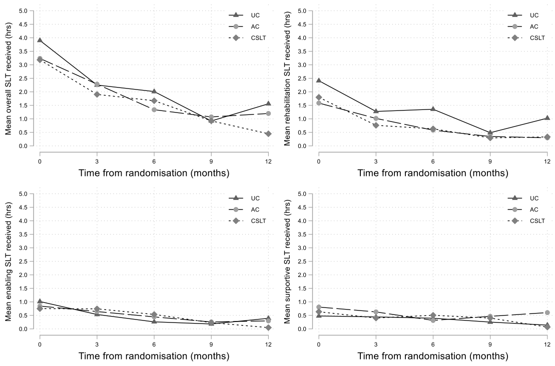

Figure 5: Mean duration of total usual care SLT received by the type of SLT (all participants)

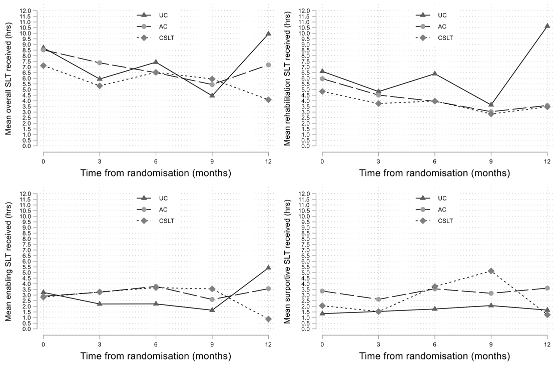

Figure 6: Mean duration of total usual care SLT given by the type of SLT (only SLT receivers)

The association between total duration of overall SLT received three months pre-assessment and time post-stroke at different assessments is displayed in Figure 7. In general, most participants who received usual care SLT received it very early on after suffering a stroke.

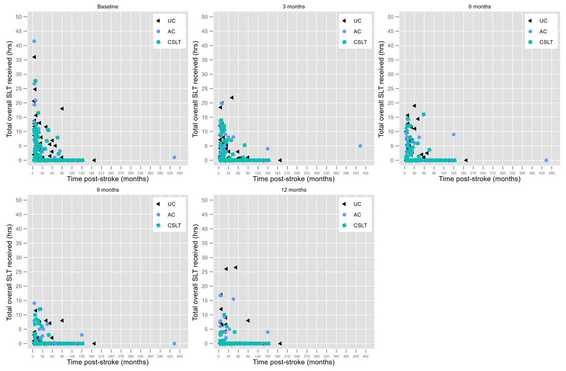

Figure 7: Relationship between the overall SLT received and time post-stroke

1.9.4 Computer use and adherence to the CSLT intervention

1.9.4.1 Patterns in computer practice

Figure 8 shows the distribution of the timing of computer therapy access since randomisation among 94 CSLT participants, excluding three deaths prior to 6-month assessment. Most participants received access to computer therapy within a month of randomisation. Two outlying participants received access to computer therapy after 3·5 months (112 and 114 days) from randomisation. The first case was due to issues with participant's computer and *StepByStep*© software version on the Big CACTUS laptop, the therapist went on sick leave and study leave, and the participant was also hospitalised with cardiac problems. For the second case, the PI went on a long-term sick leave and later resigned – so there was no one available to support the participant.

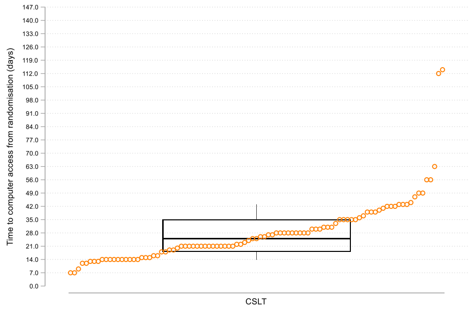

Figure 8: Delay in time to access computer therapy

The patterns of computer practice time per participant from randomisation including continued computer use after six months (dotted vertical line at ~183 days) are displayed in Figure 9. Each line regardless of the colour represents the participant's computer practice activity during the course of the trial. Of the 94 CSLT participants, 57 (60·6%) continued to use the computer therapy beyond the six months of randomisation.

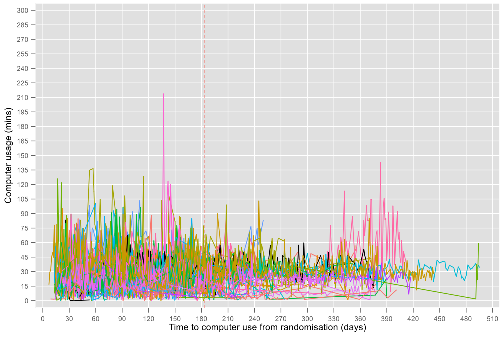

Figure 9: Computer therapy practice time per participant over time

The distributions of total computer practice time and the mean computer practice time per month and per week within six months from randomisation stratified by continued computer use (beyond six months) are displayed in Figure 10. The corresponding distribution of the total number of computer sessions and the average number of computer sessions contributing to computer practice time within six months are shown in Figure 11.

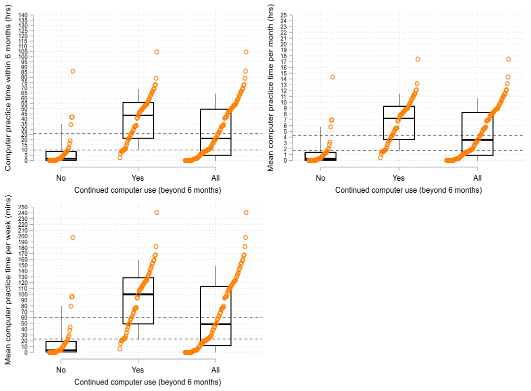

Figure 10: Distribution of computer use within 6 months

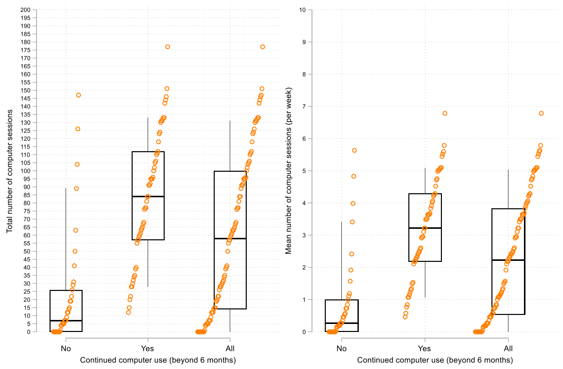

Figure 11: The distribution of computer practice sessions within 6 months

The distributions that are shown in Figure 10 and Figure 11 are summarised in Table 6. For instance, the median total computer practice time per participant within six months was 21·1 hours, with an IQR of 4·9 to 49·7 and a maximum of 104·5 hours.

Table 6: Summary of the distribution of computer practice time (N=94)

Computer use classification	Mean(SD)	Median(IQR)	Min, Max
**Computer practice time within 6 months**			
Total (hrs)	28·0(25·6)	21·1(4·9, 49·7)	0·0, 104·5
Average per month (hrs)	4·7(4·3)	3·5(0·8, 8·3)	0·0, 17·4
Average per week (mins)	64(59)	49(11, 114)	0, 240
**Number of computer sessions**			
Total	60(49)	58(14, 100)	0, 177
Average per week	2·3(1·9)	2·2(0·5, 3·8)	0·0, 6·8

Note: (Min, Max), (minimum, maximum); SD, standard deviation; IQR, interquartile range (25th, 75th percentiles)

1.9.4.2 Per-protocol adherence

Of the 97 participants randomised to receive CSLT, three died prior to 6-month assessment and were excluded in the analysis. Of the remaining 94, 60 (63·8%) and 43 (45·7%) used the computer therapy for at least ten hours and 26 hours within six months of randomisation, respectively. These were deemed to have adhered to the key components of the CSLT intervention as described in Section 1.1.6.3. In addition, no computer use was recorded for 11 participants (11·7%) at all within six months of randomisation and reasons are summarised in Table 7

Table 7: Reasons for no record of computer therapy use at all (n=11)

Number	Reason
1	Withdrew from intervention due to illness
1	Delayed access due to technical issues with the tablet, key file corrupted, could not manage to use a computer and participant withdrew from the study
3	key files corrupted or lost
1	Unable to contact participant so no 6 month assessment was done or a key file extracted
1	PI did not extract a key file as participant did not practice and withdrew early due to illness issues
1	Felt it would be too big a commitment and withdrew the same day they received access
1	Problems in using touch screen causing participant upset, PI did not extract a key file as participant did not practice and discontinued from the study
1	Broken laptop so no key file was extracted by the PI
1	Felt they cannot cope or manage laptop by self, recently unwell and lives alone so withdrew the same day they received access

Note: PI, principal investigator; key file, StepByStep© data file that was copied from the laptop/tablet of the participant to show their practice data

1.9.5 Puzzle books and adherence to the AC intervention

Only one of the 80 participants randomised to receive the AC intervention died before 6-month assessment and was excluded in the analysis. Of the remaining 79, only 14 (17·7%) were sent at least six puzzle books within six months. In addition, only these 14 (17·7%) who were sent at least six puzzle books were also contacted for at least four times within six months. Figure 12 shows the distribution of the number of books sent and successful contacts made within six months of randomisation.

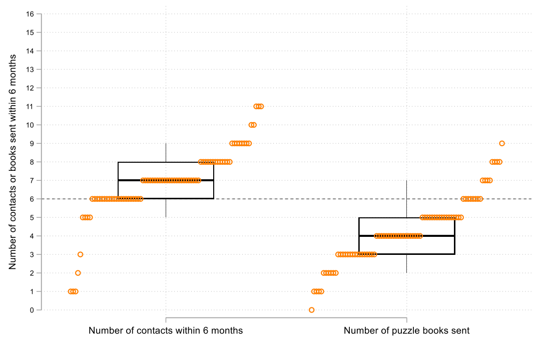

Figure 12: Distribution of puzzle books sent and successful contacts made within six months

1.9.6 Unblinding of outcome assessments

At 6-month assessment, 29 (10·7%) cases of suspected unblinding were reported and only in one case the allocated intervention was incorrectly guessed. Majority of cases happened before or during the 6-month assessment, and the proportions of participants were higher in the CSLT and AC compared to the UC. However, the reported cases of unblinding are negligible relative to the number of outcome assessments made during the trial. Table 8 summarises cases of suspected unblinding and when the allocated intervention was guessed correctly.

Table 8: Cases of unblinding of outcome assessments

Classification	Timepoint	UC (n=97)	AC (n=79)	CSLT (n=94)
Suspected unblinding	6 months	3(3·1%)	9(11·3%)	17(18·1%)
	9 months	2(2·1%)	4(5·0%)	3(3·2%)
	12 months	0(0·0%)	1(1·3%)	1(1·1%)
Unblinding [a]	6 months	2(2·1%)	8(10·0%)	17(18·1%)
	9 months	1(1·0%)	4(5·0%)	3(3·2%)
	12 months	0(0·0%)	0(0·0%)	1(1·1%)

Note: [a] correctly guessed the intervention

1.9.7 Participants response profile for the primary and key secondary endpoints over time

The changes in participants' responses with respect to word finding over time stratified by intervention are shown in Figure 13. Each line regardless of colour indicates participant's response profile. There appears to be marked improvements in the CSLT compared to the UC or AC. The responses are further stratified by baseline severity of word finding difficulty (mild, moderate, and severe) as shown in Figure 15. The figure suggests general improvements in the CSLT across the severity of word finding categories, however, marked improvements in word finding were observed in the mild and moderate categories compared to a severe subgroup (see Section 1.1.9.8.4).

The pattern in functional communication responses is unclear but appears to be similar across interventions and word finding severity categories as illustrated in Figure 16 and Figure 17. The COAST response patterns over time stratified by intervention and also by the severity of word finding difficulty are displayed in Figure 18 and Figure 19, respectively. Participants in the AC appear to have deteriorated at 6 months from baseline. The interpretation of these visual response plots should be complemented by main and subgroup results presented in Sections 1.1.9.8 and 1.1.9.8.4, respectively.

Only five (1·9%) participants had TOMS rating on the ceiling (score of 5): 3/94 (3·2%) in the CSLT and 2/97 (2·1%) in the UC. These participants cannot show any further improvements in functional communication during the trial since they were deemed to communicate effectively in all situations at baseline. There were no participants with a TOMS rating score of 0 (unable to communicate in any way) at baseline.

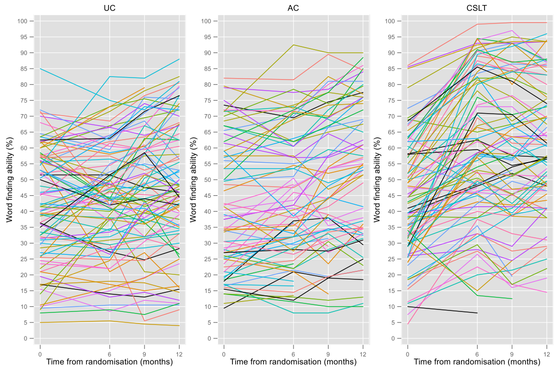

Figure 13: Changes in word finding over time stratified by the intervention

Only ten participants in the CSLT failed to improve their word finding ability at six months; computer therapy use was not recorded at all on three participants. The distribution of computer practice time for these participants is shown in Figure 14.

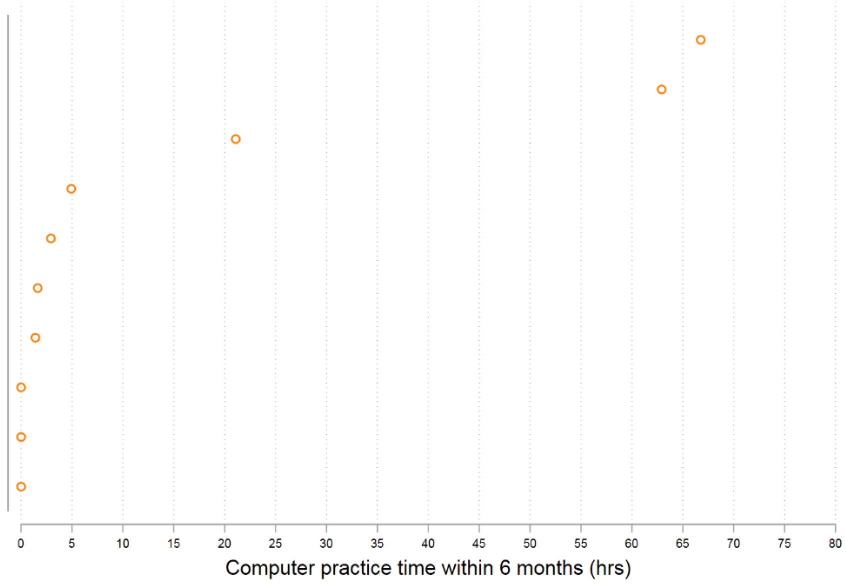

Figure 14: Computer practice time of CSLT participants who fail to improve word finding

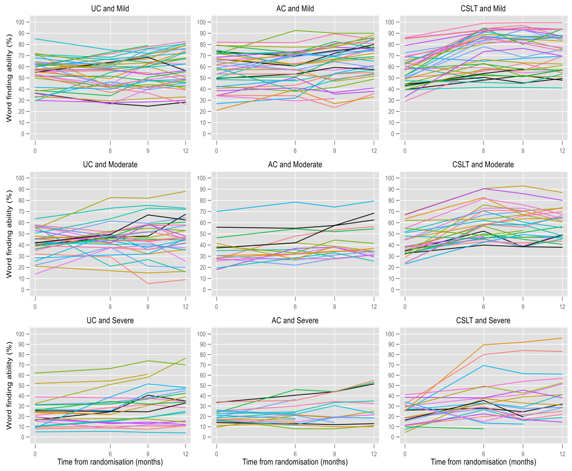

Figure 15: Changes in word finding over time stratified by the intervention and severity of word finding

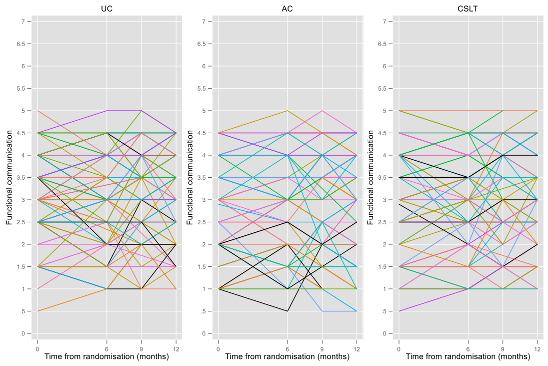

Figure 16: Changes in conversation over time stratified by the intervention

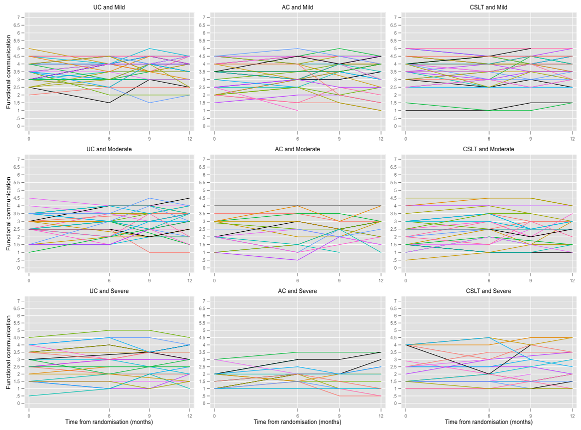

Figure 17: Changes in conversation over time stratified by intervention and severity of word finding

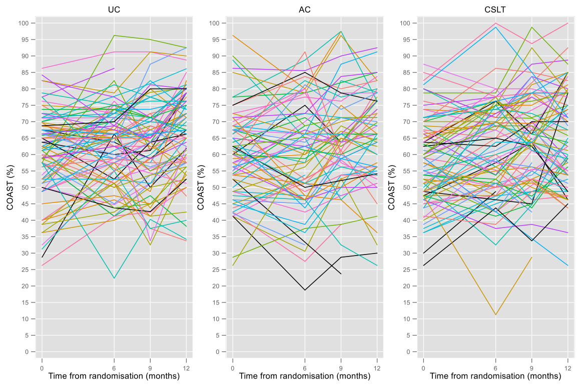

Figure 18: Changes in COAST over time stratified by intervention

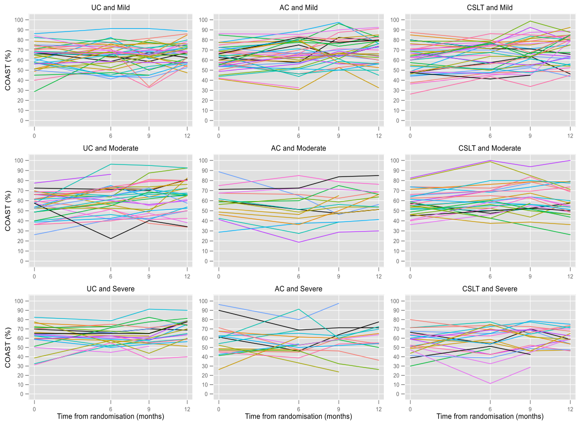

Figure 19: Changes in COAST over time stratified by intervention and severity of word finding

1.9.8 Effect of the intervention on the co-primary and key secondary endpoints

The first three primary clinical effectiveness objectives are to establish whether self-managed CSLT intervention increases the ability of people with aphasia to use the vocabulary of personal importance, improves functional communication ability in conversation, and results in perceived greater changes in social participation in daily activities and quality of life. This section addresses these objectives. The effect of the intervention on the word finding ability, functional communication, and patient perception of communication effectiveness and its impact on their quality of life at six months are presented in Table 9.

1.9.8.1 Word finding and functional communication: co-primary endpoints

For word finding, 86, 71, and 83 participants in the UC, AC, and CSLT were included in the mITT analysis, respectively. The mean improvement in word finding of personally selected words (SD) was 1·1% (11·2) in the UC and 2·4% (8·8) in the AC compared to 16·4% (15·3) in the CSLT, indicating an adjusted mean difference in word finding improvement of 16·2% (95%CI: 12·7 to 19·6; $p<0·0001$) in favour of the CSLT compared to the UC. Figure 20 and Figure 21 display the unadjusted mean responses in word finding and mean change in word finding over time stratified by the intervention, respectively.

As shown in Table 9, the mean change in functional communication was very similar between the CSLT and UC with an adjusted mean difference in change of -0·03 (95%CI: -0·21 to 0·14; $p=0·709$) slightly in favour of the UC. In line with the pre-specified Hochberg multiple testing strategy, shown in Figure 1, we can only claim the clinical effectiveness of the CSLT compared to the UC in improving word finding of personal importance (at 2.5% nominal level).

Since both comparisons between CSLT and UC with respect to word finding and functional communication were not statistically significant at 5% significance level, further statistical significance testing is prohibited (Figure 1). However, the mean improvement in word finding of personally selected words of 14.4% (10.8% to 18.1%) in favour of the CSLT compared to the AC supports that the clinical effectiveness in improving word finding of personally selected words is attributed to the CSLT intervention rather than the attention provided. The mean improvements in word finding between the AC and UC were similar although slightly in favour of the UC; 1.8% (95%CI: -1.9 to 5.4). The mean changes in functional communication were very similar across interventions (Table 9, Figure 22, and Figure 23). Thus, there is insufficient evidence to support that the CSLT intervention improves functional communication ability in conversation.

The effects of the intervention on word finding and functional communication were very similar after adjusting for additional covariates (time post-stroke and the location of stroke) (Table 9 versus Table 10).

Table 9: Co-primary and key secondary outcomes at six months (mITT)

Co-primary and key secondary outcomes at 6m	UC		AC		CSLT		CSLT vs UC †		CSLT vs AC ‡		AC vs UC †	
	n	Mean(SD)	n	Mean(SD)	n	Mean(SD)	Adjusted MDC (95%CI)	P-value	Adjusted MDC (95%CI)	P-value	Adjusted MDC (95%CI)	P-value
**Co-primary outcomes**												
Change in word finding (%)[1]	86	1.1(11.2)	71	2.4(8.8)	83	16.4(15.3)	16.2(12.7, 19.6) [a]	<0.0001	14.4(10.8, 18.1) [c]	<0.0001	1.8(-1.9, 5.4)	0.338
Change in functional communication [2]	84	0.05(0.59)	68	0.10(0.61)	81	0.04(0.58)	-0.03(-0.21, 0.14) [b]	0.709	-0.01(-0.20, 0.18) [d]	0.915	-0.02(-0.21, 0.17)	0.812
**Key secondary outcome**												
Change in COAST (%) [3]	83	2.7(12.6)	68	-0.3(12.7)	82	3.3(11.3)	0.5(-3.1, 4.1) [e]	0.772	3.8(-0.0, 7.5) [f]	0.051	-3.2(-7.0, 0.5)	0.089

Note: Results based on a multiple linear regression model adjusted for baseline measures and fixed stratification factors (centre and severity of word finding)
[a, b, c,d, e, f] are referenced in Figure 1 to aid interpretation of Hochberg sequential and hierarchical hypotheses testing procedure for decision-making
† UC as the reference group; ‡ AC as the reference group; MDC, mean difference in change; SD, standard deviation; CI, confidence interval
Interpretation: [1] higher scores indicate improved vocabulary of personal importance; [2] higher scores indicate improved functional communication ability in conversation, [3] higher percentage score indicates improved patient perception of communication effectiveness and its impact on their quality of life.

Table 10: Sensitivity analysis on the co-primary and key secondary outcomes at six months (mITT)

Sensitivity analysis: Co-primary and key secondary outcomes at 6m	UC		AC		CSLT		CSLT vs UC †		CSLT vs AC ‡		AC vs UC †	
	n	Mean(SD)	n	Mean(SD)	n	Mean(SD)	Adjusted MDC (95%CI)	P-value	Adjusted MDC (95%CI)	P-value	Adjusted MDC (95%CI)	P-value
**Co-primary outcomes**												
Change in word finding (%)	86	1.1(11.2)	71	2.4(8.8)	83	16.4(15.3)	16.3(12.8, 19.8)	<0.0001	14.7(11.0, 18.4)	<0.0001	1.6(-2.1, 5.4)	0.385
Change in functional communication	84	0.05(0.59)	68	0.10(0.61)	81	0.04(0.58)	-0.05(-0.23, 0.13)	0.596	-0.03(-0.22, 0.16)	0.781	-0.02(-0.22, 0.17)	0.830
**Key secondary outcome**												
Change in COAST (%)	83	2.7(12.6)	68	-0.3(12.7)	82	3.3(11.3)	0.9(-2.8, 4.5)	0.644	3.6(-0.2, 7.5)	0.064	-2.8(-6.6, 1.1)	0.156

Note: Sensitivity analysis results from multiple linear regression models adjusted for baseline measures, fixed stratification factors (centre and severity of word finding), and potential confounders (length of time post stroke and location of stroke)
† UC as the reference group; ‡ AC as the reference group; MDC, mean difference in change; SD, standard deviation; CI: confidence interval
Interpretation: [1] higher scores indicate improved personal vocabulary of personal importance; [2] higher scores indicate improved functional communication ability in conversation; [3] higher percentage score indicates improved patient perception of communication effectiveness and its impact on their quality of life.

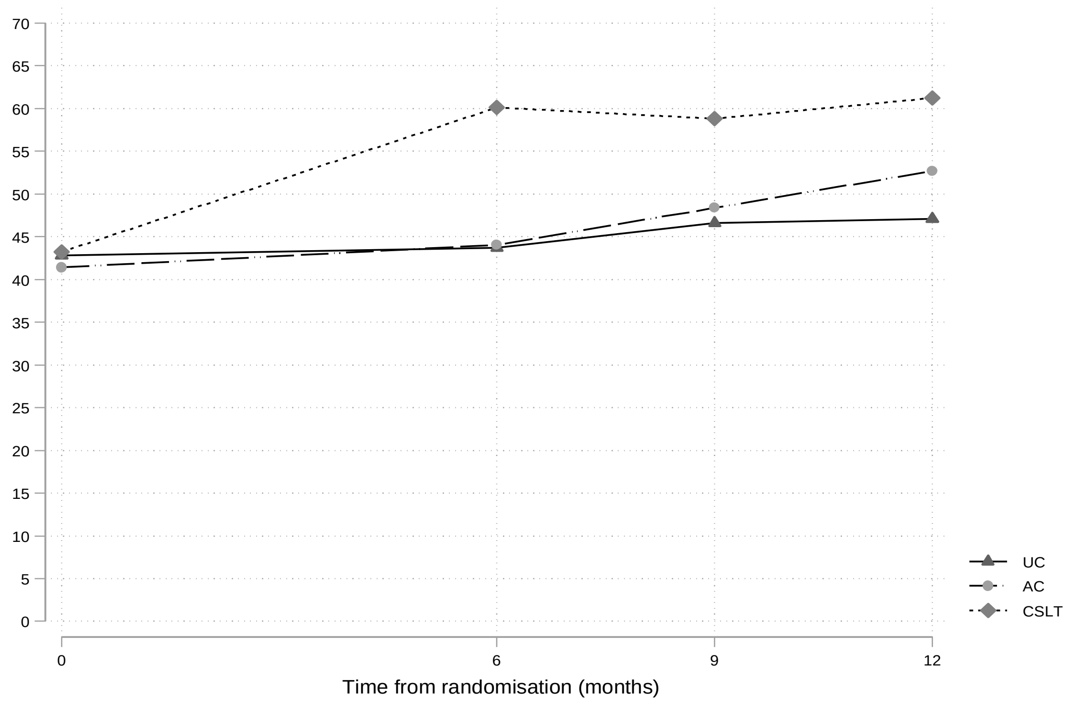

Figure 20: Mean word finding ability over time stratified by the intervention

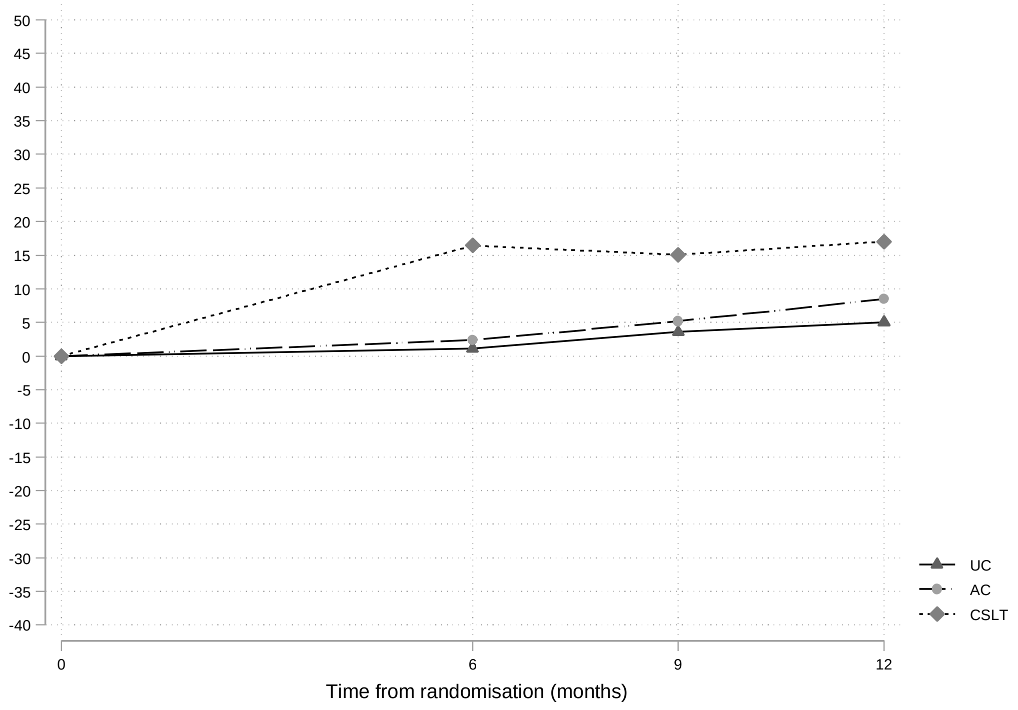

Figure 21: Mean change word finding ability over time stratified by the intervention

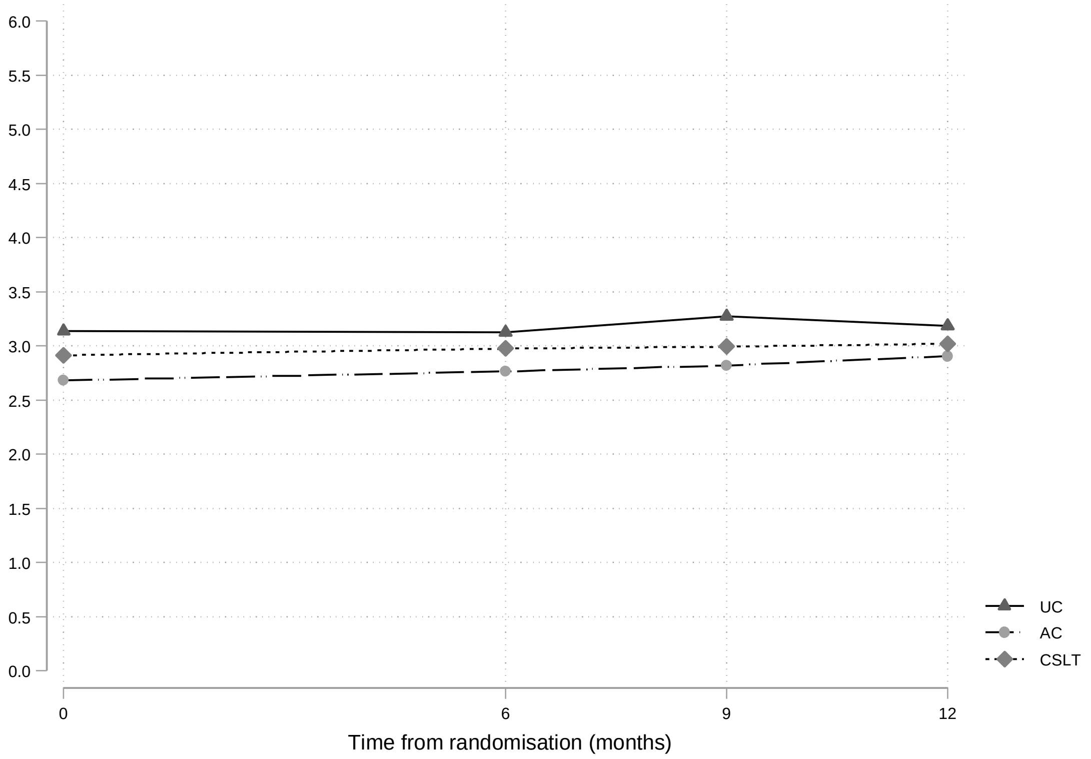

Figure 22: Mean functional communication over time stratified by the intervention

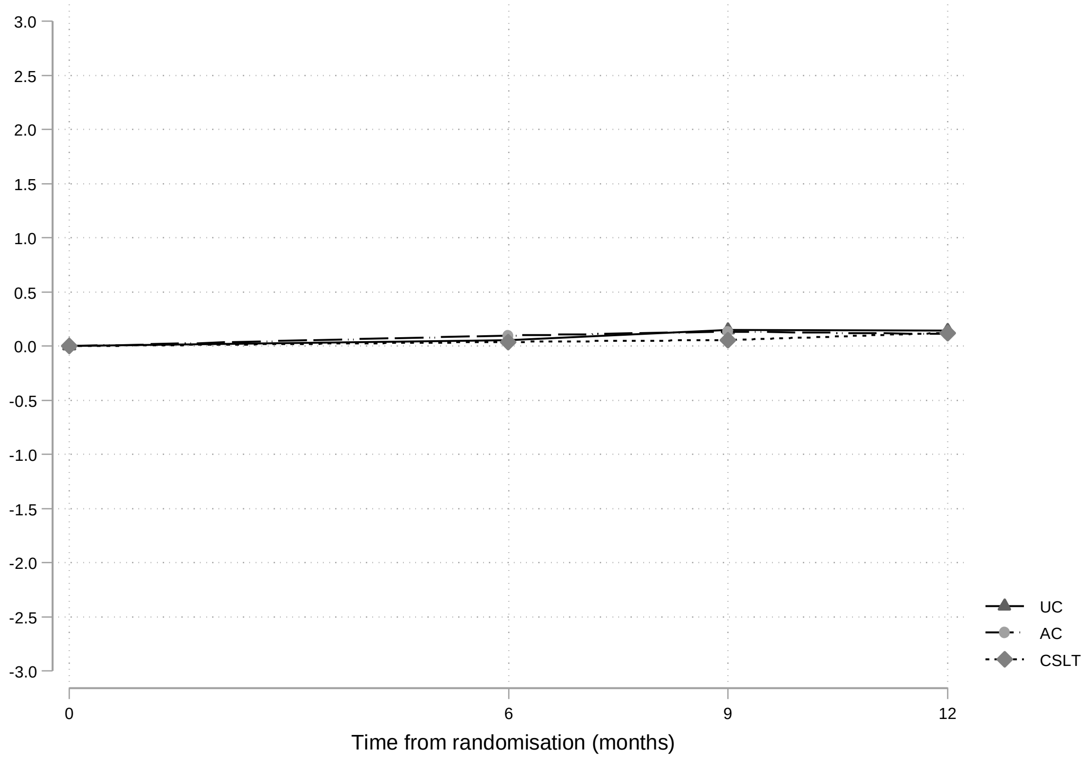

Figure 23: Mean change in functional communication over time stratified by the intervention

1.9.8.2 Patient perception of communication and its impact on their life (key secondary endpoint)

Table 9 summarises the effect of the intervention on the COAST at six months. The average improvement in the COAST was only 3.3% and 2.7% in the CSLT and UC respectively – whereas the AC decreased slightly by 0.3%. The unadjusted average profile of the COAST and change in COAST over time are displayed in Figure 24 and Figure 25, respectively. The adjusted mean difference in change in COAST was only 0.5% (95%CI: -3.1 to 4.1) marginally in favour of the CSLT compared to the UC. The adjusted mean difference in change of 3.8% (95%CI: -0.0 to 7.5) observed in the CSLT compared to the AC was due to the fact that the AC barely changed on average at six months whilst the UC experienced small average improvement which was comparable to the CSLT. In summary, there is insufficient evidence to support that the intervention improves the patient's perception of communication and its impact on their life.

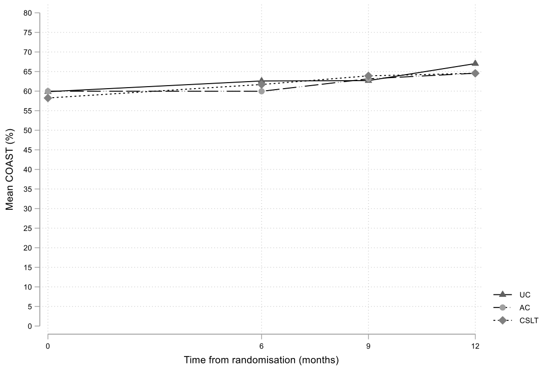

Figure 24: Mean COAST over time stratified by the intervention

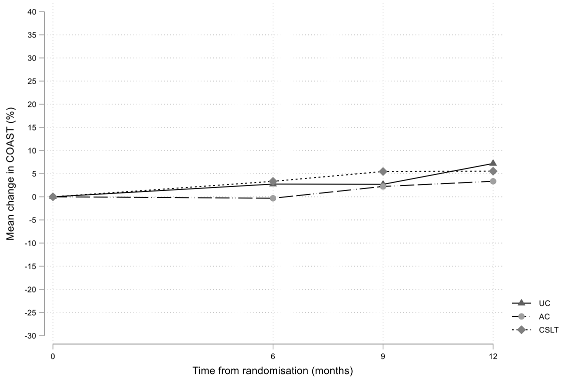

Figure 25: Mean change in COAST over time stratified by the intervention

1.9.8.3 Impact of attrition and adherence: co-primary and key secondary endpoints

As part of sensitivity analysis, this section presents results exploring the influence of attrition and intervention adherence on the effect of the intervention on word finding, functional communication, and COAST at six months. Figure 26, Figure 27, and Figure 28 display the corresponding results on forest plots for the MI set, four PP sets, and LI set described in Section 1.1.6 and how they compare with the primary mITT results presented in Table 9. In general, the main results are consistent across all analysis sets and similar to the primary analysis sets. Detailed results displayed in these forests plots are summarised in Table 11 and Table 12 for additional information. The results of PP comparisons that involve the AC should be treated with caution because of small sample size due to poor adherence in the AC, as described in Section 1.1.9.5.

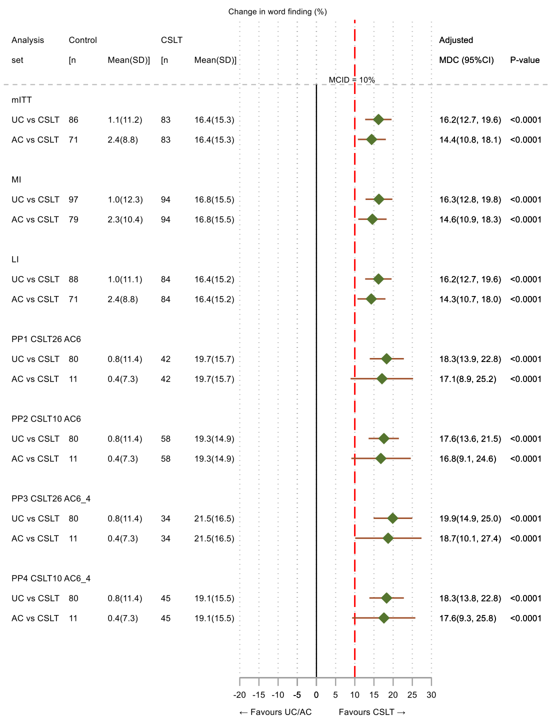

Figure 26: Impact of attrition and intervention adherence on word finding at six months

Note: (mITT, modified intention-to-treat; CC, complete case; MI, multiple imputation; LI, linear interpolation; PP – per protocol sets are defined in Section 1.1.6.3)

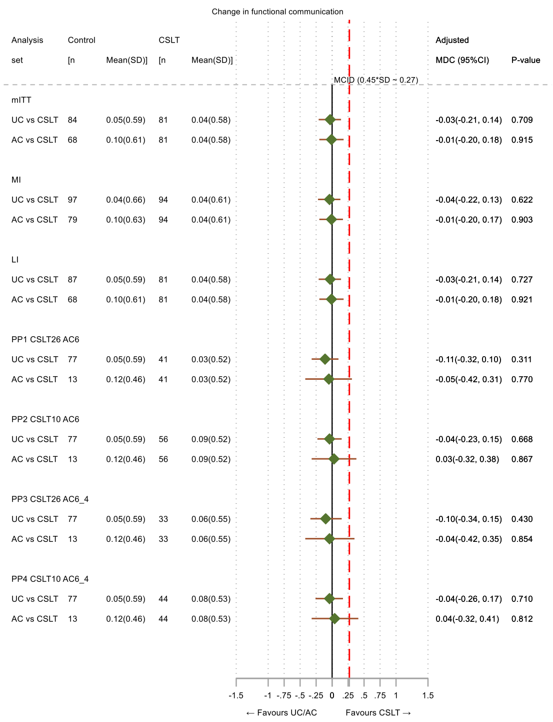

Figure 27: Impact of attrition and intervention adherence on functional communication at six months

Note: (mITT, modified intention-to-treat; CC, complete case; MI, multiple imputation; LI, linear interpolation; PP – per protocol sets are defined in Section 1.1.6.3)

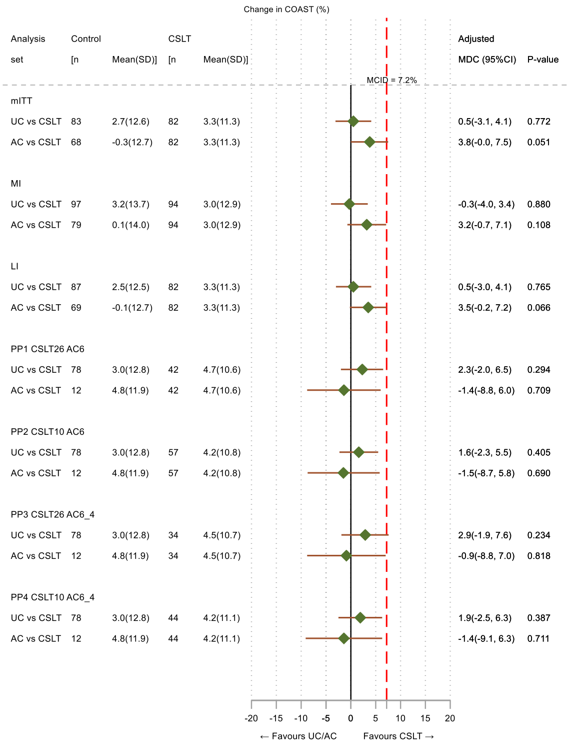

Figure 28: Impact of attrition and intervention adherence on COAST at six months

Note: (mITT, modified intention-to-treat; CC, complete case; MI, multiple imputation; LI, linear interpolation; PP – per protocol sets are defined in Section 1.1.6.3)

Table 11: Impact of adherence on the intervention effect on co-primary and key secondary endpoints

Primary and key secondary outcomes at 6m and PP set	UC		AC		CSLT		CSLT vs UC †		CSLT vs AC ‡		AC vs UC †	
	n	Mean(SD)	n	Mean(SD)	n	Mean(SD)	Adjusted MDC (95%CI)	P-value	Adjusted MDC (95%CI)	P-value	Adjusted MDC (95%CI)	P-value
**Co-primary outcomes**												
Change in word finding (%) [1]												
PP1 CSLT26 AC6	80	0.8(11.4)	11	0.4(7.3)	42	19.7(15.7)	18.3(13.9, 22.8)	<0.0001	17.1(8.9, 25.2)	<0.0001	1.3(-6.5, 9.0)	0.750
PP2 CSLT10 AC6	80	0.8(11.4)	11	0.4(7.3)	58	19.3(14.9)	17.6(13.6, 21.5)	<0.0001	16.8(9.1, 24.6)	<0.0001	0.7(-6.8, 8.3)	0.847
PP3 CSLT26 AC6_4	80	0.8(11.4)	11	0.4(7.3)	34	21.5(16.5)	19.9(14.9, 25.0)	<0.0001	18.7(10.1, 27.4)	<0.0001	1.2(-6.7, 9.2)	0.763
PP4 CSLT10 AC6_4	80	0.8(11.4)	11	0.4(7.3)	45	19.1(15.5)	18.3(13.8, 22.8)	<0.0001	17.6(9.3, 25.8)	<0.0001	0.7(-7.1, 8.6)	0.857
Change in functional communication [2]												
PP1 CSLT26 AC6	77	0.05(0.59)	12	0.08(0.47)	41	0.03(0.52)	-0.11(-0.32, 0.10)	0.311	-0.02(-0.40, 0.35)	0.903	-0.09(-0.45, 0.27)	0.634
PP2 CSLT10 AC6	77	0.05(0.59)	12	0.08(0.47)	56	0.09(0.52)	-0.04(-0.23, 0.15)	0.661	0.06(-0.30, 0.42)	0.739	-0.10(-0.45, 0.25)	0.564
PP3 CSLT26 AC6_4	77	0.05(0.59)	12	0.08(0.47)	33	0.06(0.55)	-0.10(-0.34, 0.15)	0.436	-0.00(-0.40, 0.40)	0.985	-0.09(-0.46, 0.28)	0.621
PP4 CSLT10 AC6_4	77	0.05(0.59)	12	0.08(0.47)	44	0.08(0.53)	-0.04(-0.26, 0.17)	0.710	0.08(-0.30, 0.46)	0.690	-0.12(-0.48, 0.24)	0.523
**Key secondary outcome**												
Change in COAST (%) [3]												
PP1 CSLT26 AC6	78	3.0(12.8)	12	4.8(11.9)	42	4.7(10.6)	2.3(-2.0, 6.5)	0.294	-1.4(-8.8, 6.0)	0.709	3.7(-3.4, 10.8)	0.308
PP2 CSLT10 AC6	78	3.0(12.8)	12	4.8(11.9)	57	4.2(10.8)	1.6(-2.3, 5.5)	0.405	-1.5(-8.7, 5.8)	0.690	3.1(-3.9, 10.1)	0.383
PP3 CSLT26 AC6_4	78	3.0(12.8)	12	4.8(11.9)	34	4.5(10.7)	2.9(-1.9, 7.6)	0.234	-0.9(-8.8, 7.0)	0.818	3.8(-3.5, 11.0)	0.304
PP4 CSLT10 AC6_4	78	3.0(12.8)	12	4.8(11.9)	44	4.2(11.1)	1.9(-2.5, 6.3)	0.387	-1.4(-9.1, 6.3)	0.711	3.4(-3.9, 10.6)	0.360

Note: PP sets "PP1 CSLT26 AC6", "PP2 CSLT10 AC6", "PP3 CSLT26 AC6_4", and "PP4 CSLT10 AC6_4" are defined in Section 1.1.6.3.
Results based on a multiple linear regression model adjusted for baseline measures and fixed stratification factors (centre and severity of word finding)
† UC as the reference group; ‡ AC as the reference group; MDC, mean difference in change; SD, standard deviation; CI, confidence interval
Interpretation: [1] higher scores indicate improved vocabulary of personal importance; [2] higher scores indicate improved functional communication ability in conversation, [3] higher percentage score indicates improved patient perception of communication effectiveness and its impact on their quality of life.

Table 12: Impact of attrition on the intervention effect on co-primary and key secondary outcomes at six months

Co-primary and key secondary outcomes at 6 months	UC		AC		CSLT		CSLT vs UC †		CSLT vs AC ‡		AC vs UC †	
	n	Mean(SD)	n	Mean(SD)	n	Mean(SD)	Adjusted MDC (95%CI)	P-value	Adjusted MDC (95%CI)	P-value	Adjusted MDC (95%CI)	P-value
**mITT**												
Change in word finding (%)	86	1.1(11.2)	71	2.4(8.8)	83	16.4(15.3)	16.2(12.7, 19.6)	<0.0001	14.4(10.8, 18.1)	<0.0001	1.8(-1.9, 5.4)	0.338
Change in functional communication	84	0.05(0.59)	68	0.10(0.61)	81	0.04(0.58)	-0.03(-0.21, 0.14)	0.709	-0.01(-0.20, 0.18)	0.915	-0.02(-0.21, 0.17)	0.812
Change in COAST (%)	83	2.7(12.6)	68	-0.3(12.7)	82	3.3(11.3)	0.5(-3.1, 4.1)	0.772	3.8(-0.0, 7.5)	0.051	-3.2(-7.0, 0.5)	0.089
**MI**												
Change in word finding (%)	97	1.0(12.3)	79	2.3(10.4)	94	16.8(15.5)	16.3(12.8, 19.8)	<0.0001	14.6(10.9, 18.3)	<0.0001	1.7(-2.0, 5.4)	0.368
Change in functional communication	97	0.04(0.66)	79	0.10(0.63)	94	0.04(0.61)	-0.04(-0.22, 0.13)	0.622	-0.01(-0.20, 0.17)	0.903	-0.03(-0.22, 0.15)	0.724
Change in COAST (%)	97	3.2(13.7)	79	0.1(14.0)	94	3.0(12.9)	-0.3(-4.0, 3.4)	0.880	3.2(-0.7, 7.1)	0.108	-3.5(-7.2, 0.3)	0.068
**LI**												
Change in word finding (%)	88	1.0(11.1)	71	2.4(8.8)	84	16.4(15.2)	16.2(12.7, 19.6)	<0.0001	14.3(10.7, 18.0)	<0.0001	1.9(-1.7, 5.4)	0.307
Change in functional communication	87	0.05(0.59)	68	0.10(0.61)	81	0.04(0.58)	-0.03(-0.21, 0.14)	0.727	-0.01(-0.20, 0.18)	0.921	-0.02(-0.21, 0.17)	0.825
Change in COAST (%)	87	2.5(12.5)	69	-0.1(12.7)	82	3.3(11.3)	0.5(-3.0, 4.1)	0.765	3.5(-0.2, 7.2)	0.066	-3.0(-6.6, 0.7)	0.111

Note: MI, multiple imputation; mITT, modified intention-to-treat; LI, linear interpolation; Results based on a multiple linear regression model adjusted for baseline measures and fixed stratification factors (centre and severity of word finding); † UC as the reference group; ‡ AC as the reference group; MDC, mean difference in change; SD, standard deviation; CI, confidence interval

Interpretation: [1] higher scores indicate improved vocabulary of personal importance; [2] higher scores indicate improved functional communication ability in conversation, [3] higher percentage score indicates improved patient perception of communication effectiveness and its impact on their quality of life.

1.9.8.4 Subgroup influence on effectiveness: co-primary and key secondary endpoints

This section presents results exploring potential heterogeneity in the intervention effect across pre-specified subgroups on word finding, functional communication, and COAST at six months. The results for the CSLT vs UC and CSLT vs AC comparisons are presented in Table 13 and graphically displayed in forests plots together with the mITT results (Figure 29, Figure 30, and Figure 31). Extreme caution should be taken when interpreting the intervention effect in the severe category of the CAT comprehension ability due to very small sample sizes. In addition, due consideration should be given to the clinical or biological plausibility of subgroup results.

For word finding, the results appear to be consistent and similar to the mITT results. However, the intervention effect seems to be more pronounced in patients that were within normal limits of the CAT comprehension ability. In general, functional communication and COAST results appear to be broadly consistent across subgroups.

Table 13. Subgroup analysis results: co-primary and key secondary outcome at six months

Co-primary and key secondary outcomes at 6 months	Subgroup	UC		AC		CSLT		CSLT vs UC †		CSLT vs AC ‡	
		n	Mean(SD)	n	Mean(SD)	n	Mean(SD)	Adjusted MDC (95%CI)	Interaction P-value	Adjusted MDC (95%CI)	Interaction P-value
	Word finding severity										
Change in word finding (%)	Mild	35	-2.7(10.3)	35	1.8(9.5)	36	16.3(14.2)	20.1(14.8, 25.5)		15.9(10.5, 21.2)	
	Moderate	29	3.9(13.1)	17	3.3(8.6)	26	18.2(12.5)	13.8(7.8, 19.9)		13.9(6.8, 21.0)	
	Severe	22	3.3(8.1)	19	2.7(7.8)	21	14.4(19.9)	12.8(5.9, 19.7)	0.255	13.2(6.1, 20.3)	0.828
Change in functional communication	Mild	35	-0.01(0.65)	35	0.04(0.59)	35	-0.13(0.51)	-0.08(-0.35, 0.18)		-0.10(-0.37, 0.17)	
	Moderate	27	0.15(0.59)	15	0.00(0.68)	26	0.29(0.49)	0.08(-0.23, 0.39)		0.33(-0.04, 0.69)	
	Severe	22	0.05(0.51)	17	0.32(0.58)	19	0.05(0.69)	-0.09(-0.44, 0.27)	0.88	-0.21(-0.59, 0.18)	0.145
Change in COAST (%)	Mild	34	1.2(12.3)	35	3.0(10.2)	36	3.6(9.7)	2.4(-3.0, 7.8)		1.7(-3.7, 7.0)	
	Moderate	28	6.0(13.6)	16	-5.2(10.3)	24	5.1(8.9)	-0.4(-6.6, 5.9)		9.0(1.6, 16.4)	
	Severe	20	0.9(11.7)	17	-2.6(17.2)	18	1.3(16.2)	-1.4(-8.8, 6.1)	0.809	3.0(-4.7, 10.6)	0.322
	CAT comprehension ability										
Change in word finding (%)	Severe	3	14.0(3.9)	3	4.0(1.7)	5	13.8(22.3)	1.5(-15.1, 18.2)		10.9(-5.7, 27.6)	
	Moderate	20	-0.5(8.8)	24	2.8(8.8)	26	17.9(14.2)	16.9(10.2, 23.6)		14.3(7.9, 20.7)	
	Mild	46	2.3(10.1)	31	2.9(9.4)	35	13.7(16.2)	13.0(7.8, 18.1)		11.0(5.5, 16.6)	
	Within normal limits	17	-2.6(15.2)	13	-0.1(8.4)	17	20.6(12.4)	25.1(17.3, 32.9)	0.034	23.6(15.1, 32.1)	0.271
Change in functional communication	Severe	3	0.83(0.58)	3	0.50(0.50)	4	0.13(0.75)	-0.46(-1.33, 0.40)		-0.07(-0.95, 0.81)	
	Moderate	20	-0.10(0.53)	22	0.02(0.59)	25	-0.02(0.64)	0.07(-0.27, 0.41)		-0.02(-0.36, 0.31)	
	Mild	45	0.04(0.63)	29	0.19(0.67)	35	0.01(0.55)	-0.09(-0.35, 0.17)		-0.13(-0.41, 0.15)	
	Within normal limits	16	0.13(0.47)	13	-0.04(0.52)	16	0.22(0.48)	0.09(-0.31, 0.49)	0.587	0.30(-0.13, 0.73)	0.541
Change in COAST (%)	Severe	3	25.0(12.3)	3	14.9(16.3)	4	-3.1(23.4)	-22.1(-39.7, -4.4)		-20.7(-38.4, -2.9)	
	Moderate	17	-0.5(9.7)	22	-3.2(16.4)	23	6.5(11.9)	5.6(-1.6, 12.8)		7.8(1.1, 14.6)	
	Mild	45	1.9(13.5)	30	0.1(9.9)	34	2.1(10.1)	-0.7(-6.0, 4.6)		2.8(-2.9, 8.5)	
	Within normal limits	17	4.3(9.8)	13	0.1(8.6)	17	3.9(8.6)	0.8(-7.0, 8.6)	0.039	5.0(-3.5, 13.5)	0.049
	Time post-stroke										
Change in word finding (%)	<Q1	24	3.4(9.2)	17	4.4(6.7)	26	14.6(19.0)	12.0(5.5, 18.6)		11.6(4.4, 18.7)	
	Q1 - <Q2	21	-1.6(14.1)	18	2.0(9.2)	20	18.2(15.7)	16.5(9.3, 23.7)		12.8(5.4, 20.3)	
	Q2 - <Q3	20	0.5(11.4)	12	4.0(9.8)	21	20.0(10.5)	20.1(12.9, 27.3)		18.5(9.9, 27.0)	

	≥Q3	21	1.8(10.0)	24	0.4(9.3)	16	12.5(13.0)	16.1(8.4, 23.9)	0.572	13.7(6.3, 21.2)	0.647
Change in functional communication	<Q1	24	0.19(0.59)	16	0.22(0.63)	25	0.02(0.70)	-0.15(-0.47, 0.18)		-0.07(-0.44, 0.30)	
	Q1 - <Q2	21	-0.17(0.58)	17	-0.03(0.65)	19	0.24(0.42)	0.34(-0.02, 0.71)		0.34(-0.04, 0.73)	
	Q2 - <Q3	19	0.21(0.54)	12	0.00(0.74)	21	-0.10(0.44)	-0.30(-0.66, 0.07)		-0.12(-0.54, 0.31)	
	≥Q3	20	-0.03(0.62)	22	0.18(0.50)	15	0.07(0.65)	-0.02(-0.42, 0.38)	0.052	-0.17(-0.56, 0.22)	0.145
Change in COAST (%)	<Q1	22	1.8(16.5)	16	2.7(10.4)	24	1.7(13.5)	0.0(-6.7, 6.8)		-1.6(-8.9, 5.7)	
	Q1 - <Q2	21	3.9(12.2)	17	0.1(14.5)	19	6.4(9.7)	1.6(-5.7, 8.8)		6.2(-1.4, 13.8)	
	Q2 - <Q3	19	5.0(10.9)	12	-8.1(11.4)	20	6.1(10.0)	1.7(-5.6, 9.1)		12.7(4.2, 21.2)	
	≥Q3	20	0.5(10.2)	23	1.4(12.4)	15	-0.5(9.9)	-0.9(-8.8, 6.9)	0.814	-0.1(-7.7, 7.5)	0.047

Note: Q1, 25th percentile~1 year; Q2, 50th percentile~2 years; Q3, 75th percentile~4 years; CAT, Comprehension Aphasia Test; Results based on a multiple linear regression model adjusted for baseline measures and fixed stratification factors (centre and severity of word finding) with interaction between intervention group and subgroup; [†]UC as the reference group; [‡] AC as the reference group; MDC, mean difference in change; SD, standard deviation; CI, confidence interval

Interpretation: [1] higher scores indicate improved vocabulary of personal importance; [2] higher scores indicate improved functional communication ability in conversation, [3] higher percentage score indicates improved patient perception of communication effectiveness and its impact on their quality of life.

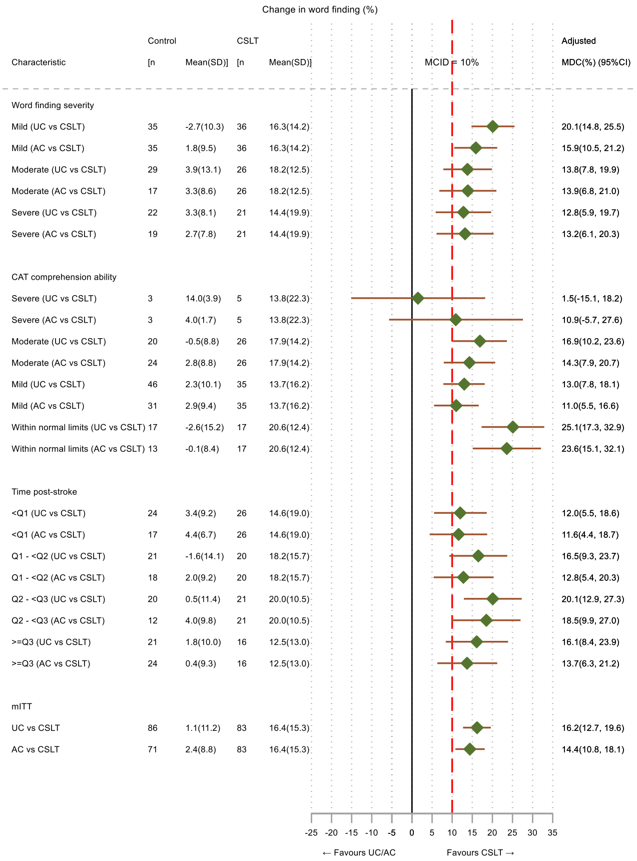

Figure 29: Subgroup influence on word finding results at six months

Note: (Q1, 25th percentile~1 year; Q2, 50th percentile~2 years; Q3, 75th percentile~4 years; CAT, Comprehension Aphasia Test; mITT, modified intention-to-treat; MCID, minimum clinically important difference; CI, confidence interval)

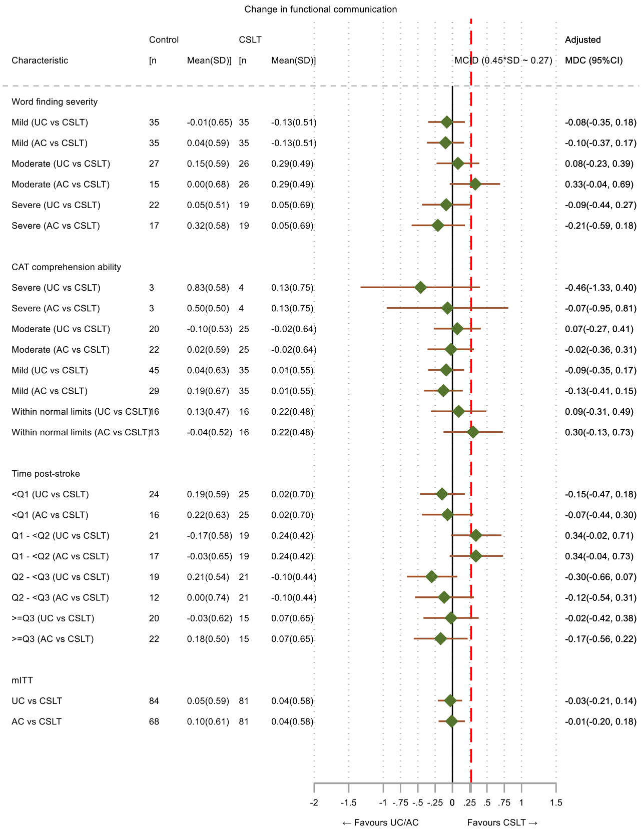

Figure 30: Subgroup influence on functional communication results at six months

Note: (Q1, 25th percentile~1 year; Q2, 50th percentile~2 years; Q3, 75th percentile~4 years; CAT, Comprehension Aphasia Test; mITT, modified intention-to-treat; MCID, minimum clinically important difference; CI, confidence interval)

Characteristic	Control [n	Mean(SD)]	CSLT [n	Mean(SD)]	Adjusted MDC(%) (95%CI)
**Word finding severity**					
Mild (UC vs CSLT)	34	1.2(12.3)	36	3.6(9.7)	2.4(-3.0, 7.8)
Mild (AC vs CSLT)	35	3.0(10.2)	36	3.6(9.7)	1.7(-3.7, 7.0)
Moderate (UC vs CSLT)	28	6.0(13.6)	24	5.1(8.9)	-0.4(-6.6, 5.9)
Moderate (AC vs CSLT)	16	-5.2(10.3)	24	5.1(8.9)	9.0(1.6, 16.4)
Severe (UC vs CSLT)	20	0.9(11.7)	18	1.3(16.2)	-1.4(-8.8, 6.1)
Severe (AC vs CSLT)	17	-2.6(17.2)	18	1.3(16.2)	3.0(-4.7, 10.6)
**CAT comprehension ability**					
Severe (UC vs CSLT)	3	25.0(12.3)	4	-3.1(23.4)	-22.1(-39.7, -4.4)
Severe (AC vs CSLT)	3	14.9(16.3)	4	-3.1(23.4)	-20.7(-38.4, -2.9)
Moderate (UC vs CSLT)	17	-0.5(9.7)	23	6.5(11.9)	5.6(-1.6, 12.8)
Moderate (AC vs CSLT)	22	-3.2(16.4)	23	6.5(11.9)	7.8(1.1, 14.6)
Mild (UC vs CSLT)	45	1.9(13.5)	34	2.1(10.1)	-0.7(-6.0, 4.6)
Mild (AC vs CSLT)	30	0.1(9.9)	34	2.1(10.1)	2.8(-2.9, 8.5)
Within normal limits (UC vs CSLT)	17	4.3(9.8)	17	3.9(8.6)	0.8(-7.0, 8.6)
Within normal limits (AC vs CSLT)	13	0.1(8.6)	17	3.9(8.6)	5.0(-3.5, 13.5)
**Time post-stroke**					
<Q1 (UC vs CSLT)	22	1.8(16.5)	24	1.7(13.5)	0.0(-6.7, 6.8)
<Q1 (AC vs CSLT)	16	2.7(10.4)	24	1.7(13.5)	-1.6(-8.9, 5.7)
Q1 - <Q2 (UC vs CSLT)	21	3.9(12.2)	19	6.4(9.7)	1.6(-5.7, 8.8)
Q1 - <Q2 (AC vs CSLT)	17	0.1(14.5)	19	6.4(9.7)	6.2(-1.4, 13.8)
Q2 - <Q3 (UC vs CSLT)	19	5.0(10.9)	20	6.1(10.0)	1.7(-5.6, 9.1)
Q2 - <Q3 (AC vs CSLT)	12	-8.1(11.4)	20	6.1(10.0)	12.7(4.2, 21.2)
>=Q3 (UC vs CSLT)	20	0.5(10.2)	15	-0.5(9.9)	-0.9(-8.8, 6.9)
>=Q3 (AC vs CSLT)	23	1.4(12.4)	15	-0.5(9.9)	-0.1(-7.7, 7.5)
**mITT**					
UC vs CSLT	83	2.7(12.6)	82	3.3(11.3)	0.5(-3.1, 4.1)
AC vs CSLT	68	-0.3(12.7)	82	3.3(11.3)	3.8(-0.0, 7.5)

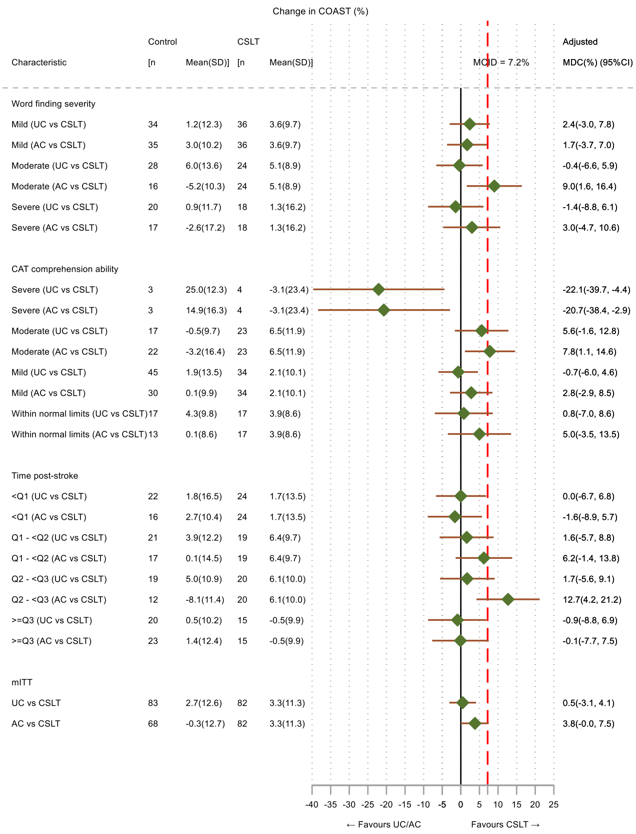

Figure 31: Subgroup influence on COAST results at six months

Note: (Q1, 25th percentile~1 year; Q2, 50th percentile~2 years; Q3, 75th percentile~4 years; CAT, Comprehension Aphasia Test; mITT, modified intention-to-treat; MCID, minimum clinically important difference; CI, confidence interval)

1.9.8.5 Long-term effects of the intervention: co-primary and key secondary outcomes

The fourth primary objective of the clinical effectiveness analysis is to identify whether any effects of the interventions are evident 12 months after therapy has begun. To address this primary objective, Figure 20 to Figure 25 display the unadjusted average profile response of participants over time for word finding, functional communication, and COAST stratified by the intervention. In addition, the effectiveness results for the co-primary and key secondary outcomes at nine and 12 months are summarised in Table 14 and graphically displayed in Figure 32 to Figure 34, together with the primary results at six months for comparability.

In summary, the short-term clinical effectiveness of the intervention on word finding of personal importance was sustained in long-term at nine and 12 months across analysis sets. For the functional communication and COAST, no changes in outcomes of clinical importance were observed and results are consistent with the primary results at six months and across the analysis sets considered.

Table 14: Long-term intervention effect on the co-primary and key secondary outcomes at nine and 12 months

Co-primary and key secondary outcomes at 9 and 12 months	UC		AC		CSLT		CSLT vs UC †		CSLT vs AC ‡		AC vs UC †	
	n	Mean(SD)	N	Mean(SD)	n	Mean(SD)	Adjusted MDC (95%CI)	P-value	Adjusted MDC (95%CI)	P-value	Adjusted MDC (95%CI)	P-value
**9 months**												
CC												
Change in word finding (%)	83	3.6(12.9)	64	5.2(9.2)	77	15.1(15.5)	12.2(8.3, 16.0)	<0.0001	10.0(5.9, 14.1)	<0.0001	2.2(-1.9, 6.2)	0.290
Change in functional communication	83	0.15(0.70)	61	0.13(0.67)	73	0.06(0.59)	-0.16(-0.35, 0.04)	0.108	0.06(-0.16, 0.27)	0.585	-0.22(-0.43, -0.00)	0.046
Change in COAST (%)	81	2.7(12.5)	61	2.2(12.8)	74	5.5(10.9)	2.7(-1.0, 6.4)	0.156	3.4(-0.6, 7.4)	0.098	-0.7(-4.6, 3.2)	0.716
MI												
Change in word finding (%)	97	3.6(13.7)	79	5.0(11.3)	94	14.9(16.2)	11.5(7.8, 15.3)	<0.0001	9.7(5.6, 13.8)	<0.0001	1.8(-2.1, 5.7)	0.352
Change in functional communication	97	0.12(0.77)	79	0.08(0.76)	94	0.05(0.70)	-0.15(-0.35, 0.05)	0.130	0.07(-0.14, 0.27)	0.533	-0.22(-0.44, 0.00)	0.053
Change in COAST (%)	97	3.0(14.2)	79	2.7(13.7)	94	5.1(12.6)	2.1(-1.5, 5.8)	0.251	2.6(-1.3, 6.6)	0.189	-0.5(-4.4, 3.4)	0.792
LI												
Change in word finding (%)	88	3.6(12.6)	71	5.3(9.2)	84	15.0(15.9)	11.9(8.2, 15.6)	<0.0001	9.7(5.8, 13.6)	<0.0001	2.2(-1.6, 6.1)	0.260
Change in functional communication	87	0.15(0.69)	68	0.12(0.74)	81	0.07(0.62)	-0.11(-0.31, 0.08)	0.250	0.08(-0.13, 0.29)	0.443	-0.20(-0.41, 0.02)	0.069
Change in COAST (%)	87	2.8(12.6)	69	3.2(14.1)	82	5.4(11.7)	2.6(-1.0, 6.3)	0.155	2.5(-1.4, 6.4)	0.203	0.1(-3.6, 3.9)	0.938
**12 months**												
CC												
Change in word finding (%)	84	5.1(15.3)	61	8.5(10.2)	74	17.0(14.2)	12.7(8.7, 16.7)	<0.0001	9.3(4.8, 13.7)	<0.0001	3.4(-0.8, 7.7)	0.115
Change in functional communication	79	0.15(0.69)	59	0.11(0.81)	70	0.12(0.60)	-0.07(-0.29, 0.14)	0.497	0.11(-0.13, 0.35)	0.366	-0.18(-0.42, 0.05)	0.125
Change in COAST (%)	82	7.2(12.4)	59	3.4(12.4)	74	5.5(12.2)	-1.0(-4.6, 2.7)	0.606	3.3(-0.7, 7.3)	0.101	-4.3(-8.1, -0.4)	0.029
MI												
Change in word finding (%)	97	5.2(16.0)	79	7.7(13.4)	94	16.1(16.6)	11.2(7.1, 15.4)	<0.0001	8.9(4.3, 13.5)	<0.0001	2.3(-2.0, 6.6)	0.291
Change in functional communication	97	0.13(0.79)	79	0.09(0.89)	94	0.12(0.74)	-0.09(-0.30, 0.12)	0.399	0.10(-0.13, 0.33)	0.383	-0.19(-0.43, 0.05)	0.112
Change in COAST (%)	97	7.4(13.7)	79	3.4(14.2)	94	5.3(13.9)	-2.3(-6.0, 1.4)	0.229	2.3(-1.7, 6.3)	0.251	-4.6(-8.6, -0.6)	0.024
LI												
Change in word finding (%)	88	5.3(15.2)	71	7.8(11.0)	84	15.8(16.2)	11.2(7.1, 15.3)	<0.0001	8.5(4.2, 12.9)	<0.0001	2.7(-1.6, 6.9)	0.216
Change in functional communication	87	0.14(0.73)	68	0.06(0.93)	81	0.14(0.75)	-0.04(-0.27, 0.20)	0.763	0.22(-0.03, 0.48)	0.084	-0.26(-0.51, -0.01)	0.045
Change in COAST (%)	87	6.8(12.4)	69	4.9(16.2)	82	6.3(12.9)	-0.9(-4.9, 3.1)	0.667	1.7(-2.5, 5.9)	0.429	-2.6(-6.7, 1.6)	0.221

Note: CC, complete case; CI, confidence interval; MI, multiple imputation; mITT, modified intention-to-treat; LI, linear interpolation; Results based on a multiple linear regression model adjusted for baseline measures and fixed stratification factors (centre and severity of word finding); † UC as the reference group; ‡ AC as the reference group; MDC, mean difference in change; SD, a standard deviation

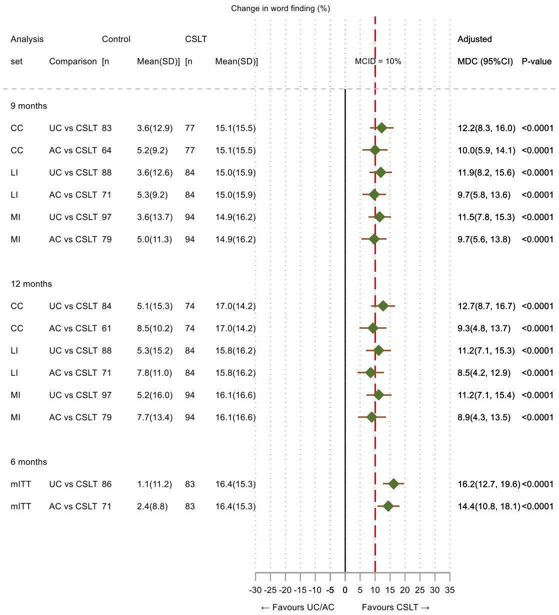

Figure 32: Long-term intervention effect on word finding of treated words

Note: (CC, complete case; CI, confidence interval; MI, multiple imputation; mITT, modified intention-to-treat; LI, linear interpolation; SD, standard deviation; MCID, minimum clinically important difference)

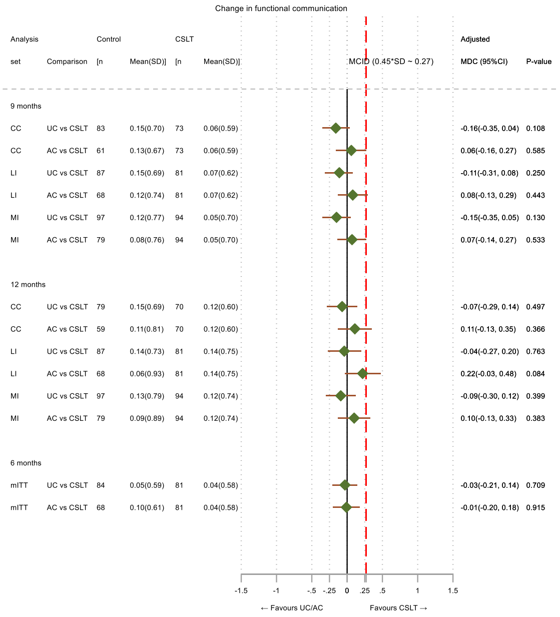

Figure 33: Long-term intervention effect on functional communication

Note: (CC, complete case; CI, confidence interval; MI, multiple imputation; mITT, modified intention-to-treat; LI, linear interpolation; SD, standard deviation; MCID, minimum clinically important difference)

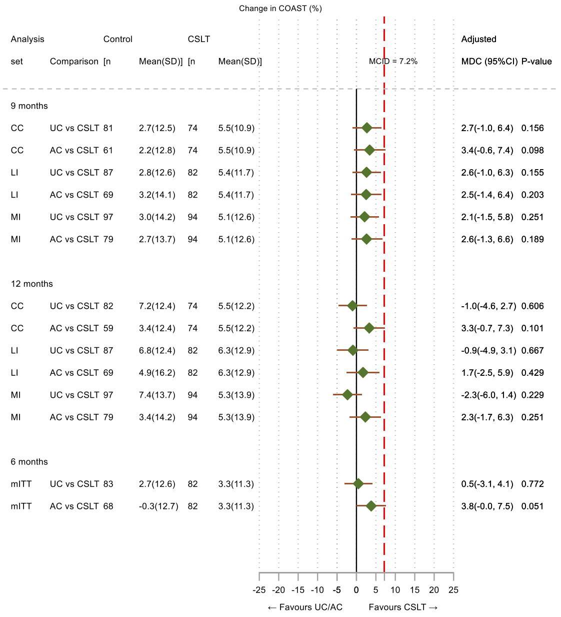

Figure 34: Long-term intervention effect COAST

Note: (CC, complete case; CI, confidence interval; MI, multiple imputation; mITT, modified intention-to-treat; LI, linear interpolation; SD, standard deviation; MCID, minimum clinically important difference)

1.9.9 Computer usage and association with change in outcomes at six months

There is debate among researchers about the amount of computer practice therapy required to benefit patients. To further this debate, the relationships between total computer practice time within six months and change in word finding, functional communication, and COAST stratified by severity of word finding difficulty are shown in Figure 35, Figure 36, and Figure 37, respectively. There appears to be some association between computer practice time and change in word finding. For instance, regarding the change in word finding, those with severe word finding difficulty at baseline appear to benefit more with increased computer practice time whereas the gains in word finding among those with mild or moderate seem to diminish with increased computer practice time beyond the total 26 hours say within six months.

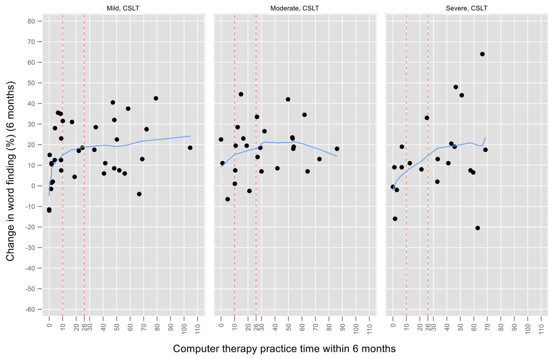

Figure 35: Association between computer use and word finding stratified by severity of word finding

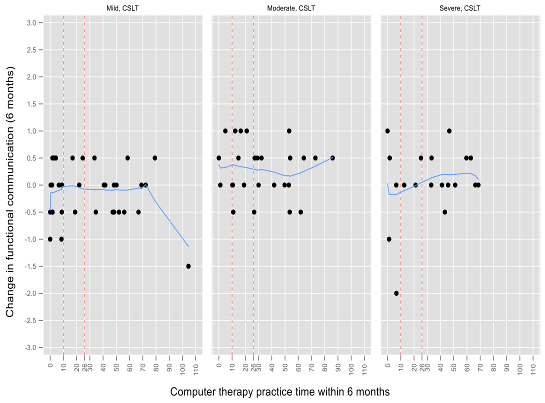

Figure 36: Association between computer use and functional communication stratified by severity of word finding

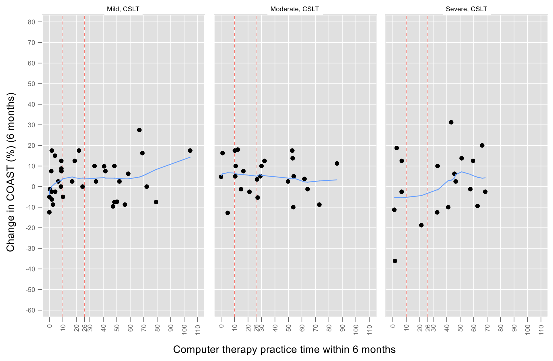

Figure 37: Association between computer use and COAST stratified by severity of word finding

1.9.10 Time post-stroke association with outcomes at 6 months

The relationships between time post-stroke and change in word finding, functional communication, and COAST stratified by severity of word finding and the intervention group are shown in Figure 38, Figure 39, and Figure 40, respectively. In summary, there appears to be no clear relationship between time post-stroke and the co-primary and key secondary endpoints.

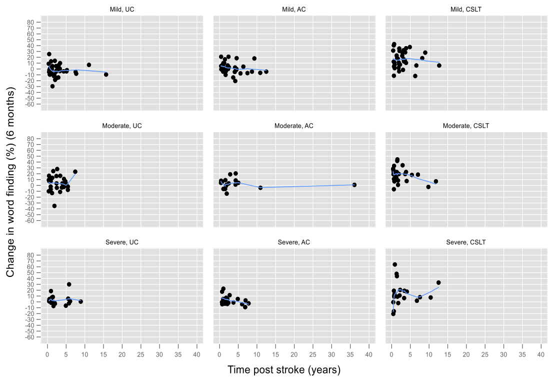

Figure 38: Relationship between time post-stroke and word finding stratified by severity of word finding

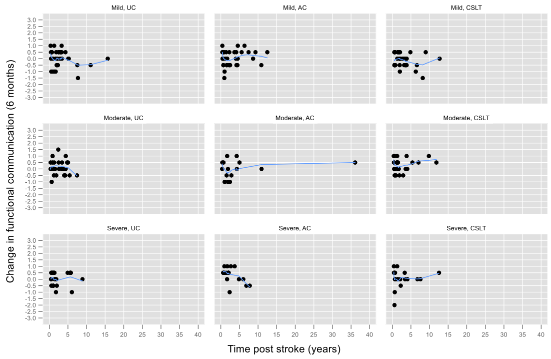

Figure 39: Relationship between time post-stroke and functional communication stratified by severity of word finding

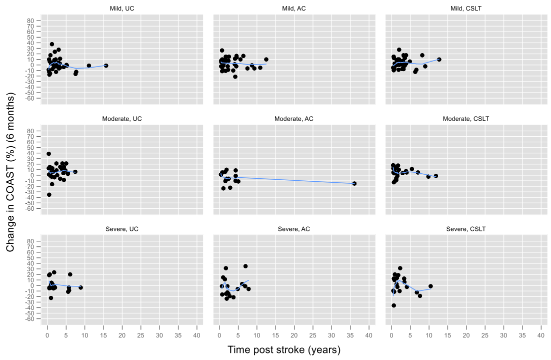

Figure 40: Relationship between time post-stroke and COAST stratified by severity of word finding

1.9.11 Association between age and outcomes at 6 months

The relationships between age and change in word finding, functional communication, or COAST stratified by severity of word finding and the intervention group are shown in Figure 41, Figure 42, and Figure 43 respectively. There seems to be no clear evidence of an association between age and key outcomes

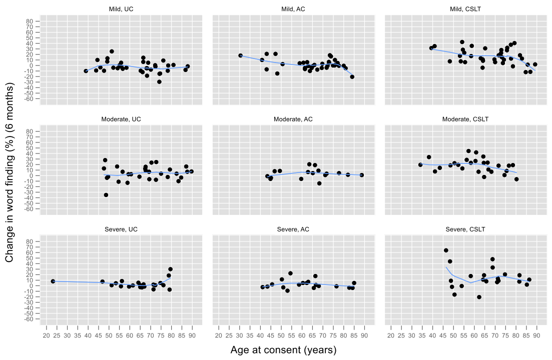

Figure 41: Relationship between age and word finding stratified by severity of word finding

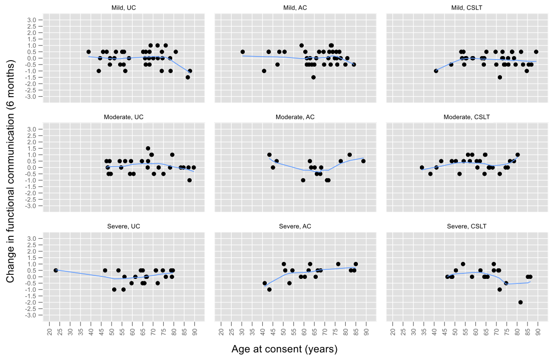

Figure 42: Relationship between age and functional communication stratified by severity of word finding

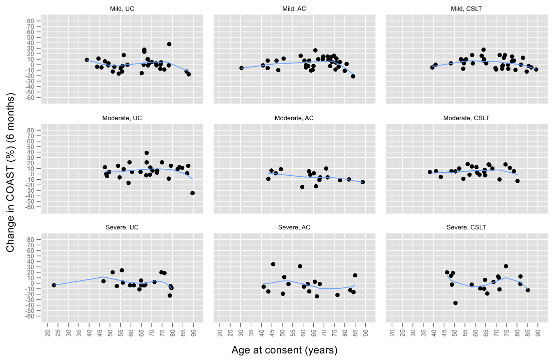

Figure 43: Relationship between age and COAST stratified by severity of word finding

1.9.12 Continued computer use and association with change in outcomes at six months

Patterns in participants' responses with respect to word finding, functional communication, and COAST are displayed in Figure 44, Figure 46, and Figure 48 stratified by whether a participant in the CSLT continued to use the computer therapy beyond the six months (yes versus no). The corresponding average response profiles are displayed in Figure 45, Figure 47, and Figure 49.

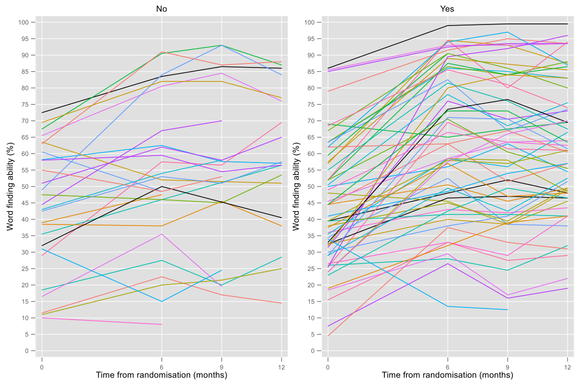

Figure 44: Changes in word finding over time stratified by continued computer use beyond six months

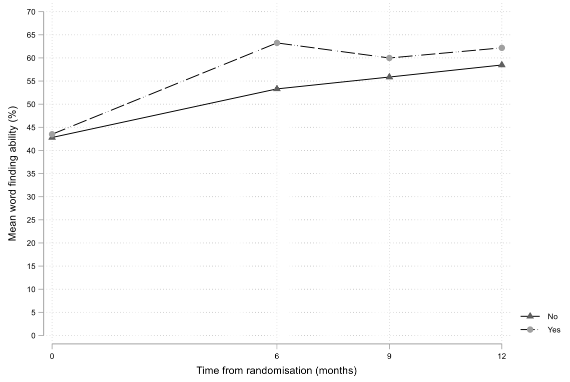

Figure 45: Average response in word finding over time stratified by continued computer use beyond six months

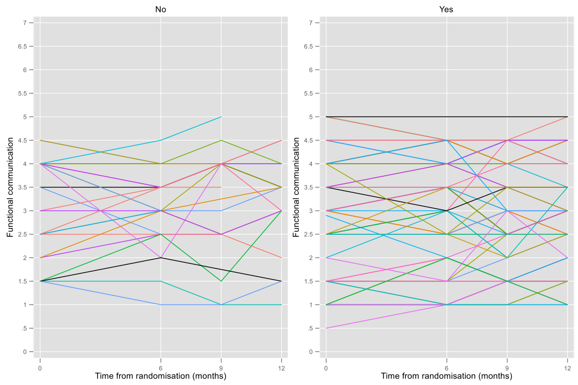

Figure 46: Changes in conversation over time stratified by continued computer use beyond six months

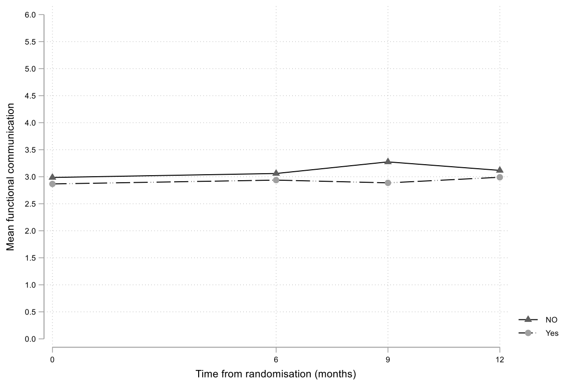

Figure 47: Average response in conversation over time stratified by continued computer use beyond six months

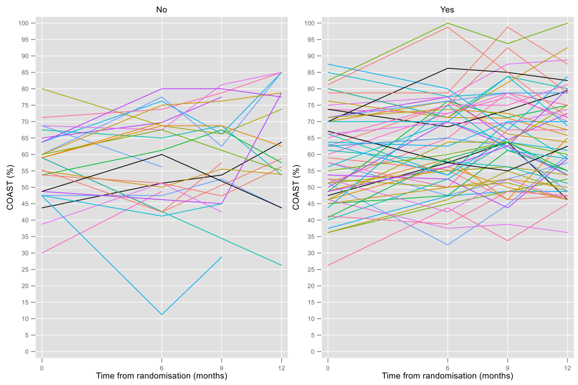

Figure 48: Changes in COAST over time stratified by continued computer use beyond six months

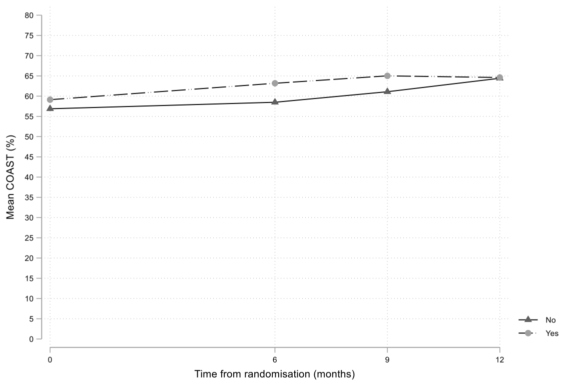

Figure 49: Average response in COAST over time stratified by continued computer use beyond six months

1.9.13 Puzzle books sent/contacts made and association with change in outcomes at six months

Figure 50 displays the relationship between the numbers of puzzle books sent to participants in the AC and change in their word finding, functional communication, and COAST at six months. In addition, the corresponding relationship between the numbers of contacts made and these outcomes at six months is shown in Figure 51. There seems to be some positive linear association between the number of puzzle books sent and the change in the COAST. However, there does not appear to be a clear association with change in word finding and functional communication.

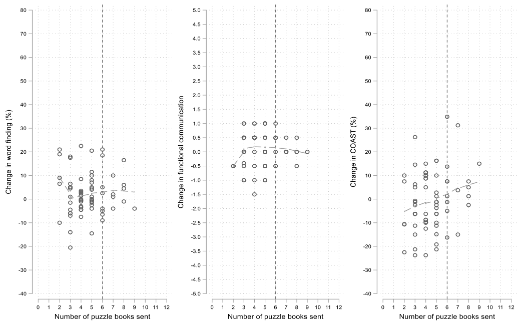

Figure 50: Relationship between the numbers of puzzle books sent and key outcomes at six months

There appears to be some positive association between the number of contacts made and change in the COAST. The association with change in word finding and functional communication is unclear (Figure 51).

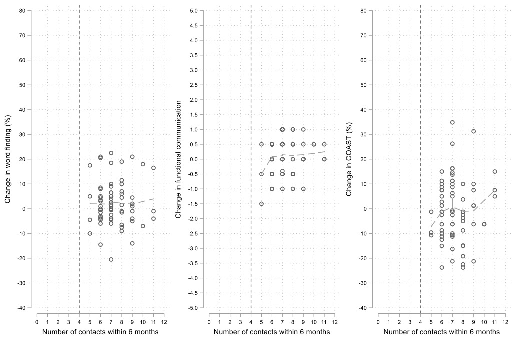

Figure 51: Relationship between the numbers of contacts made and key outcomes at six months

1.9.14 Intervention effect on generalisation to untreated words

In this section, results exploring whether there is a generalisation of word finding of personal importance (treated words) to untreated words (from CAT Naming Test) are presented. A possible total score from the picture naming tasks of 24 untreated words ranges from 0 to 48. Table 15 summarises the number of participants with missing outcome data during follow-up.

Table 15: Data completeness in word finding of untreated words

Assessment	UC (n=97)	AC (n=79)	CSLT (n=94)
6 months	11(11·3%)	10(12·7%)	12(12·8%)
9 months	14(14·4%)	16(20·3%)	18(19·1%)
12 months	14(14·4%)	19(24·1%)	20(21·3%)

Figure 52 displays the mean response profile of word finding of untreated words during follow-up. Table 16 summarises the effect of the intervention on word finding of untreated words at six, nine, and 12 months.

At six months, the mean change (SD) in word finding of untreated words was 3·9 (7·9), 0·7 (8·5), 3·3 (7·0) in the UC, AC, and CSLT, respectively. This indicates an adjusted mean difference in change of -0·3 (95%CI: -2·7 to 2·1) in favour of the UC compared to the CSLT. On average, the word finding of untreated words of participants in the AC was lower than the UC and CSLT across assessments (Figure 52). In summary, there is no sufficient evidence to support the positive effect of the intervention in improving generalisation of word finding to untreated words either in short or long-term (Table 16 and Figure 52).

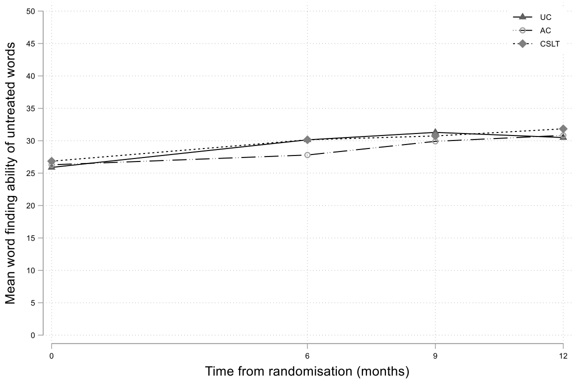

Figure 52: Mean response profile in word finding of untreated words over time

Table 16: Intervention effect on generalisation to untreated words at 6, 9 and 12 months

Change in word finding of untreated words	UC		AC		CSLT		CSLT vs UC †		CSLT vs AC ‡		AC vs UC †	
	n	Mean(SD)	n	Mean(SD)	n	Mean(SD)	Adjusted MDC (95%CI)	P-value	Adjusted MDC (95%CI)	P-value	Adjusted MDC (95%CI)	P-value
**CC**												
6 months	86	3·9(7·9)	69	0·7(8·5)	82	3·3(7·0)	-0·3(-2·7, 2·1)	0·810	2·6(0·1, 5·1)	0·045	-2·9(-5·3, -0·4)	0·025
9 months	83	4·8(8·0)	63	2·5(6·9)	76	4·0(7·9)	-0·7(-3·1, 1·6)	0·534	1·4(-1·2, 3·9)	0·299	-2·1(-4·6, 0·4)	0·100
12 months	83	4·5(8·5)	60	2·8(7·1)	74	4·8(7·3)	0·6(-1·8, 3·0)	0·634	1·8(-0·8, 4·5)	0·177	-1·3(-3·9, 1·3)	0·342
**MI**												
6 months	97	3·9(8·9)	79	0·8(8·6)	94	3·4(7·9)	-0·4(-2·7, 2·0)	0·754	2·4(-0·1, 4·9)	0·058	-2·8(-5·3, -0·3)	0·030
9 months	97	5·0(8·7)	79	2·4(7·8)	94	3·9(8·8)	-1·0(-3·3, 1·3)	0·396	1·4(-1·2, 3·9)	0·288	-2·4(-4·9, 0·1)	0·064
12 months	97	4·8(9·2)	79	2·3(8·3)	94	4·7(8·4)	-0·1(-2·5, 2·4)	0·967	2·1(-0·5, 4·6)	0·117	-2·1(-4·7, 0·5)	0·108
**LI**												
6 months	88	3·8(7·9)	70	0·7(8·4)	83	3·3(7·0)	-0·2(-2·5, 2·2)	0·893	2·5(0·0, 5·0)	0·047	-2·7(-5·1, -0·2)	0·032
9 months	88	4·6(8·2)	70	2·0(8·3)	83	4·0(7·8)	-0·3(-2·7, 2·1)	0·808	2·0(-0·6, 4·6)	0·131	-2·3(-4·8, 0·3)	0·078
12 months	88	4·3(9·1)	70	2·0(9·6)	83	4·8(7·9)	0·8(-1·9, 3·4)	0·571	2·7(-0·2, 5·5)	0·064	-1·9(-4·7, 0·9)	0·179

Note: Total score from the picture naming tasks ranges from 0 to 48; CC, complete case; MI, multiple imputation; LI, linear interpolation; Results based on a multiple linear regression model adjusted for baseline measures and fixed stratification factors (centre and severity of word finding); † UC as the reference group; ‡ AC as the reference group; MDC: mean difference in change; SD: standard deviation; CI: confidence interval; positive higher scores indicate improved word finding of untreated words

1.9.14.1 Clinical improvement in generalisation to untreated words

It was highlighted that the literature often reports the proportion of participants who do or not generalise word finding to untreated words. In consonant with the literature, a post hoc analysis was performed to estimate the proportion of participants who generalised word finding of untreated words as defined by a clinical improvement of at least 5% and 10% from baseline. As shown in Table 17, 47 (54·7%) in the UC, 34 (49·3%) in the AC, and 39 (47·6%) in the CSLT recorded a clinical improvement of at least 5% at six months from baseline. The odds of clinical improvement in word finding of untreated words were slightly in favour of the UC compared to CSLT at six and nine months, as supported by ORs below 1. However, there is insufficient evidence to suggest the differences in the proportions of participants showing clinical improvement in word finding of untreated words since the CIs around ORs fail to exclude the null intervention effect of 1. At 12 months, the proportion of participants achieving a clinical improvement in word finding of at least 5% and 10% were very similar across interventions. In summary, these results do not support that the intervention increases the proportion of participants achieving a clinical improvement of at least 5% and 10% in the generalisation of word finding to untreated words.

Table 17: Clinical improvement in generalisation to untreated words

Change in word finding of untreated words	UC	AC	CSLT	CSLT vs UC † OR(95%CI)	CSLT vs AC ‡ OR(95%CI)	AC vs UC † OR(95%CI)
**6 months**	(n=86)	(n=69)	(n=82)			
≥5%	47(54·7%)	34(49·3%)	39(47·6%)	0·7(0·4, 1·4)	0·9(0·4, 1·8)	0·8(0·4, 1·6)
≥10%	35(40·7%)	26(37·7%)	31(37·8%)	0·9(0·4, 1·7)	0·9(0·4, 1·9)	1·0(0·5, 1·9)
**9 months**	(n=83)	(n=63)	(n=76)			
≥5%	53(63·9%)	33(52·4%)	40(52·6%)	0·6(0·3, 1·2)	1·0(0·5, 2·2)	0·6(0·3, 1·2)
≥10%	41(49·4%)	25(39·7%)	31(40·8%)	0·7(0·4, 1·4)	1·0(0·5, 2·2)	0·7(0·3, 1·4)
**12 months**	(n=83)	(n=60)	(n=74)			
≥5%	49(59·0%)	35(58·3%)	46(62·2%)	1·4(0·7, 2·7)	1·2(0·6, 2·6)	1·1(0·5, 2·3)
≥10%	37(44·6%)	26(43·3%)	30(40·5%)	1·0(0·5, 2·0)	0·9(0·4, 1·9)	1·1(0·5, 2·3)

Note: Complete cases only; Results based on a multiple logistic regression model adjusted for baseline word finding of untreated words score and fixed stratification factors (centre and severity of word finding); † UC as the reference group; ‡ AC as the reference group; OR: Odds Ratio; CI: confidence interval; OR above 1 means higher odd of showing clinical improvement in the intervention compared to the comparator; OR below 0 means higher odd of showing clinical improvement in the comparator compared to the intervention.

1.9.14.2 Clinical word finding improvement in treated and untreated words

This section presents results exploring the effect of the intervention on the proportion of participants achieving pre-defined clinical improvement in both word finding of treated words (from Personal Vocabulary Naming Test) and untreated words (scores from the CAT Naming Test). In general, the proportion of participants achieving clinical improvement is higher in the CSLT compared to either UC or AC. Table 18 presents results only for participants with complete data. At six months, 27 (32·9%) in the CSLT and 12 (14·0%) in the UC achieved at least 10% change in word finding of both treated and untreated words, indicating a 4·3 (95%CI: 1·8 to 10·3) odds of achieving clinical improvement in the CSLT compared to the UC. In general, the proportion participants achieving at least a 5% or 10% clinical improvement in both the word finding of treated words and untreated words is higher in the CSLT compared to UC or AC at six, nine and 12 months. Table 19 presents sensitivity analysis results assuming participants with missing data did not achieve the desired clinical improvements and the results appear to be consistent with those presented in Table 18.

Table 18: Clinical improvement in word finding of treated and untreated words (complete data)

Change in word finding of treated [a] and untreated words [b]	UC	AC	CSLT	CSLT vs UC†		CSLT vs AC‡		AC vs UC†	
				OR(95%CI)	P-value	OR(95%CI)	P-value	OR(95%CI)	P-value
**6 months**	(n=86)	(n=69)	(n=82)						
≥5%	25(29·1%)	16(23·2%)	35(42·7%)	2·2(1·1, 4·6)	0·027	2·7(1·2, 5·8)	0·014	0·8 (0·4, 1·9)	0·677
≥10%	12(14·0%)	7(10·1%)	27(32·9%)	4·3(1·8, 10·3)	0·001	5·1(1·9, 13·9)	0·001	0·8 (0·3, 2·5)	0·749
≥10% (treated words) and ≥5% (untreated words)	13(15·1%)	9(13·0%)	31(37·8%)	4·8(2·1, 11·2)	<0·001	4·7(1·9, 11·8)	0·001	1·0 (0·4, 2·8)	0·958
**9 months**	(n=83)	(n=63)	(n=76)						
≥5%	33(39·8%)	22(34·9%)	35(46·1%)	1·5(0·8, 3·1)	0·239	1·8(0·8, 4·0)	0·133	0·8 (0·4, 1·8)	0·664
≥10%	21(25·3%)	13(20·6%)	23(30·3%)	1·5(0·7, 3·2)	0·338	1·7(0·7, 4·0)	0·254	0·9 (0·4, 2·1)	0·768
≥10% (treated words) and ≥5% (untreated words)	22(26·5%)	15(23·8%)	28(36·8%)	1·9(0·9, 4·1)	0·083	2·0(0·9, 4·5)	0·108	1·0 (0·4, 2·3)	0·972
**12 months**	(n=83)	(n=60)	(n=74)						
≥5%	35(42·2%)	28(46·7%)	40(54·1%)	2·2(1·1, 4·5)	0·034	1·6(0·7, 3·4)	0·260	1·4 (0·7, 3·0)	0·386
≥10%	23(27·7%)	15(25·0%)	28(37·8%)	2·4(1·1, 5·3)	0·030	2·3(1·0, 5·6)	0·061	1·0 (0·4, 2·5)	0·928
≥10% (treated words) and ≥5% (untreated words)	26(31·3%)	18(30·0%)	36(48·6%)	3·3(1·5, 7·2)	0·002	3·3(1·4, 7·7)	0·006	1·0 (0·4, 2·3)	0·991

Note: Results based on a multiple logistic regression model adjusted for baseline measures and fixed stratification factors (centre and severity of word finding); † UC as the reference group; ‡ AC as the reference group; OR, Odds Ratio; CI, confidence interval; [a] from the Personal Vocabulary Naming Test; [b] from the CAT Naming Test

Table 19: Clinical improvement in word finding of treated and untreated words (worst-case scenario)

Change in word finding of treated [a] and untreated words [b]	UC	AC	CSLT	CSLT vs UC†		CSLT vs AC‡		AC vs UC†	
				OR(95%CI)	P-value	OR(95%CI)	P-value	OR(95%CI)	P-value
**6 months**	(n=97)	(n=79)	(n=94)						
≥5%	25(25·8%)	16(20·3%)	35(37·2%)	2·0(1·0, 3·9)	0·046	2·4(1·1, 5·0)	0·024	0·8 (0·4, 1·8)	0·657
≥10%	12(12·4%)	7(8·9%)	27(28·7%)	3·8(1·6, 8·7)	0·002	4·5(1·7, 12·0)	0·002	0·8 (0·3, 2·4)	0·726
≥10% (treated words) and ≥5% (untreated words)	13(13·4%)	9(11·4%)	31(33·0%)	4·2(1·9, 9·3)	0·001	4·1(1·7, 10·0)	0·002	1·0 (0·4, 2·7)	0·981
**9 months**	(n=97)	(n=79)	(n=94)						
≥5%	33(34·0%)	22(27·8%)	35(37·2%)	1·3(0·7, 2·5)	0·434	1·6(0·8, 3·3)	0·173	0·8 (0·4, 1·6)	0·514
≥10%	21(21·6%)	13(16·5%)	23(24·5%)	1·3(0·6, 2·7)	0·516	1·5(0·7, 3·5)	0·297	0·8 (0·4, 1·9)	0·642
≥10% (treated words) and ≥5% (untreated words)	22(22·7%)	15(19·0%)	28(29·8%)	1·6(0·8, 3·3)	0·176	1·8(0·8, 3·8)	0·151	0·9 (0·4, 2·0)	0·842
**12 months**	(n=97)	(n=79)	(n=94)						
≥5%	35(36·1%)	28(35·4%)	40(42·6%)	1·4(0·8, 2·7)	0·272	1·3(0·7, 2·5)	0·455	1·1 (0·6, 2·2)	0·771
≥10%	23(23·7%)	15(19·0%)	28(29·8%)	1·5(0·8, 3·2)	0·234	1·8(0·8, 4·0)	0·143	0·9 (0·4, 1·9)	0·710
≥10% (treated words) and ≥5% (untreated words)	26(26·8%)	18(22·8%)	36(38·3%)	2·0(1·0, 3·9)	0·049	2·2(1·1, 4·7)	0·030	0·9 (0·4, 1·9)	0·731

Note: No change assumed for missing data; Results based on a multiple logistic regression model adjusted for baseline measures and fixed stratification factors (centre and severity of word finding); † UC as the reference group; ‡ AC as the reference group; OR, Odds Ratio; CI, confidence interval; [a] from the Personal Vocabulary Naming Test; [b] from the CAT Naming Test.

1.9.15 Intervention effect on generalisation of treated words used in conversation

This section presents the results of the intervention effect on the use of learned vocabulary (treated words finding) in conversation based on a possible total number of unique words retrieved in video conversations ranging from 0 to 100. The underlying distribution of the numbers of treated words used in conversation is shown in Figure 53 (clustered boxplots) stratified by intervention at different assessments, including the baseline.

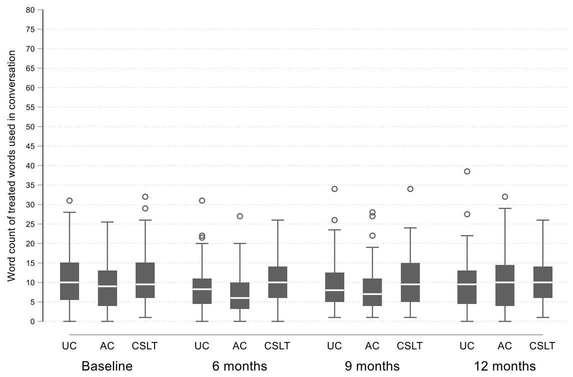

Figure 53: Distribution of the numbers of treated words retrieved in conversation over time

The average response profiles in the numbers of treated words used in conversation over time are shown in Figure 54. The UC and AC experienced an average decrease of about two treated words at six months while the CSLT remained almost the same throughout the trial. This translated to an adjusted mean difference in change in treated words used in conversation at six months of 2·0 (95%CI: 0·6 to 3·4) and 2·9 (95%CI: 1·4 to 4·4) in favour of the CLST compared to the UC and AC, respectively. The effect diminished at nine and 12 months as the UC and AC improved slightly on average. Table 20 detail results that are very consistent across analysis sets considered at six, nine, and 12 months.

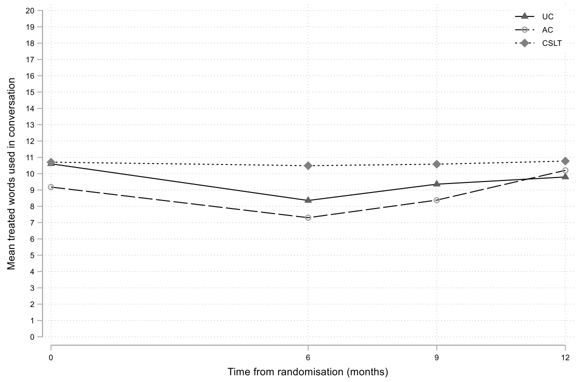

Figure 54: Mean response profile in word finding of treated words over time

Table 20: Intervention effect on generalisation of treated words used in conversation

Change in treated words used in conversation	UC		AC		CSLT		CSLT vs UC †		CSLT vs AC ‡		AC vs UC †	
	n	Mean(SD)	n	Mean(SD)	n	Mean(SD)	Adjusted MDC (95%CI)	P-value	Adjusted MDC (95%CI)	P-value	Adjusted MDC (95%CI)	P-value
**CC**												
6 months	84	-1·9(5·3)	68	-2·0(5·2)	81	0·1(6·6)	2·0(0·6 to 3·4)	0·006	2·9(1·4 to 4·4)	<0·001	-0·9(-2·4 to 0·6)	0·241
9 months	83	-1·0(5·6)	61	-1·4(5·7)	73	0·4(7·0)	1·4(-0·3 to 3·0)	0·099	2·2(0·4 to 4·0)	0·017	-0·8(-2·6 to 0·9)	0·357
12 months	79	-0·4(5·5)	59	0·1(6·6)	70	0·5(5·6)	0·8(-0·8 to 2·4)	0·351	0·8(-1·0 to 2·5)	0·401	0·0(-1·7 to 1·7)	0·996
**MI**												
6 months	97	-2·0(6·0)	79	-1·7(5·8)	94	-0·3(7·0)	2·0(0·5, 3·5)	0·009	2·8(1·2, 4·3)	<0·001	-0·8(-2·3, 0·7)	0·317
9 months	97	-1·3(6·3)	79	-1·2(6·5)	94	-0·1(7·4)	1·3(-0·3, 2·9)	0·103	2·2(0·4, 4·1)	0·017	-0·9(-2·7, 0·9)	0·330
12 months	97	-0·4(6·2)	79	0·5(6·8)	94	0·3(6·6)	0·7(-0·8, 2·3)	0·354	0·8(-0·9, 2·5)	0·347	-0·1(-1·8, 1·6)	0·908
**LI**												
6 months	87	-1·9(5·2)	68	-1·8(5·3)	81	0·3(6·8)	2·2(0·8, 3·6)	0·003	2·9(1·4, 4·4)	<0·001	-0·8(-2·3, 0·7)	0·308
9 months	87	-1·1(5·7)	68	-1·1(5·7)	81	0·5(7·4)	1·6(0·0, 3·1)	0·047	2·3(0·7, 4·0)	0·006	-0·8(-2·4, 0·9)	0·361
12 months	87	-0·6(5·8)	68	0·3(6·4)	81	0·4(7·0)	0·8(-0·8, 2·5)	0·328	0·9(-0·9, 2·7)	0·311	-0·1(-1·8, 1·7)	0·915

Note: Total unique words retrieved from the video conversations range from 0 to 100; CC, complete case; MI, multiple imputation; LI, linear interpolation; Results based on a multiple linear regression model adjusted for baseline measures and fixed stratification factors (centre and severity of word finding); † UC as the reference group; ‡ AC as the reference group; MDC, mean difference in change; SD, standard deviation; CI, confidence interval; high positive scores indicate improved word finding of untreated words

1.9.15.1 Effect of volunteer/assistant on generalisation of treated words used in conversation

This section explores the effect of having a volunteer or assistant on the generalisation of treated words used in conversation only for the CSLT participants. The underlying distributions of change in the treated words used in conversation and whether a participant had access to a volunteer or assistant for a minimum of four visits (including scheduled and unscheduled visits) if they wished at six, nine and 12 months are shown in Figure 55 (clustered boxplots). In addition, Figure 56 shows the relationship between changes in treated words used in conversation and the number of volunteer or assistant contacts made at six, nine and 12 months.

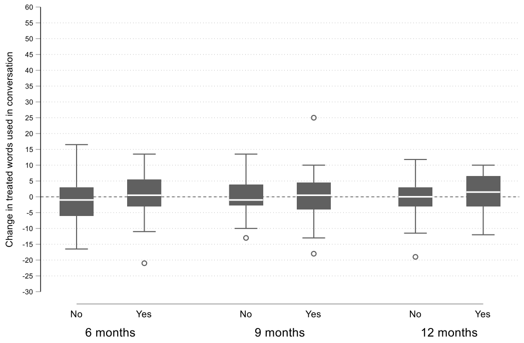

Figure 55: Distribution of change in treated words used in conversation over time

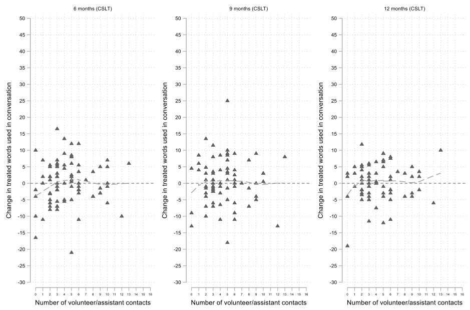

Figure 56: Association between change in treated words used in conversation and the number of contacts made

1.9.15.2 Clinical improvement in generalisation of treated words used in conversation

This section presents post hoc analysis results of the proportion of participants meeting clinical improvement in the generalisation of treated words used in conversation (words retrieved during videoed conversations) of ≥5 and ≥10 words. As shown in Table 21, 23 (28·4%) in the CSLT, 6 (8·8%) in the AC, and 8 (9·5%) recorded a clinical improvement of at least five words at six months from baseline. That is, only about 1 in 10 participants in the UC or AC showed a clinical improvement of at least five words compared to approximately 3 in 10 in the CSLT. This indicates an 18·9% or 19·6% increase in the proportion of participants showing clinical improvement in the generalisation of treated words used in conversation in the CSLT compared to the AC or UC, respectively at six months. A handful of participants improved by more than ten words across interventions in short and long-term at six, nine and 12 months.

Table 21: Clinical improvement in generalisation of treated words used in conversation

Change in treated words used in conversation	UC	AC	CSLT	CSLT vs UC† Difference in proportions (95%CI)	CSLT vs AC‡ Difference in proportions (95%CI)	AC vs UC† Difference in proportions (95%CI)
**6 months**	(n=84)	(n=68)	(n=81)			
≥5 words	8(9·5%)	6(8·8%)	23(28·4%)	18·9(7·2, 30·5)	19·6(7·7, 31·5)	-0·7 (-9·9, 8·5)
≥10 words	1(1·2%)	0(0·0%)	5(6·2%)	5·0(-0·7, 10·7)	6·2(0·9, 11·4)	-1·2 (-3·5, 1·1)
**9 months**	(n=83)	(n=61)	(n=73)			
≥5 words	11(13·3%)	8(13·1%)	15(20·5%)	7·3(-4·5, 19·1)	7·4(-5·1, 20·0)	-0·1 (-11·3, 11·0)
≥10 words	3(3·6%)	1(1·6%)	4(5·5%)	1·9(-4·7, 8·5)	3·8(-2·3, 10·0)	-2·0 (-7·1, 3·2)
**12 months**	(n=79)	(n=59)	(n=70)			
≥5 words	12(15·2%)	12(20·3%)	18(25·7%)	10·5(-2·4, 23·5)	5·4(-9·1, 19·9)	5·1 (-7·8, 18·1)
≥10 words	3(3·8%)	4(6·8%)	2(2·9%)	-0·9(-6·7, 4·8)	-3·9(-11·4, 3·6)	3·0 (-4·7, 10·7)

Note: Complete cases only; Results obtained using the normal approximation to the binomial distribution; † UC as the reference group; ‡ AC as the reference group; CI, confidence interval.

1.9.15.3 Clinical improvement in word finding of personally selected (treated) words and treated words used in conversation

In this section, based on post hoc analysis, we explore the effect of the intervention on the proportion of participants achieving clinical improvement in word finding of treated words (from Personal Vocabulary Naming Test) and treated words used in conversation (from words retrieved during videoed conversation) relative to their baseline. No participants in the UC and only 1 (1·4%) in the AC achieved a clinical improvement in word finding of treated words (personal selected) of at least 5% and treated words used in conversation of at least five words compared to 21 (25·3%) in the CSLT at six months. This indicates a 25·3% (95% CI: 15·9 to 34·7) and 23·9% (95% CI: 14·1 to 33·6) difference in the proportions of participants achieving clinical improvement of 5% in favour of the CSLT compared to UC or AC, respectively. In general, the proportion of participants achieving clinical improvement of at least a 10% treated words and ten treated words used in conversation was either very small or zero across interventions and assessments. Results at six, nine, and 12 months are detailed in Table 22.

Table 22: Clinical improvement in word finding of treated words and treated words used in conversation

Change in word finding of treated words (%) and treated words used in conversation	UC	AC	CSLT	CSLT vs UC† Difference in proportions (95%CI)	CSLT vs AC ‡ Difference in proportions (95%CI)
**6 months**	(n=86)	(n=71)	(n=83)		
≥5% and ≥5 words	0(0·0%)	1(1·4%)	21(25·3%)	25·3(15·9, 34·7)	23·9(14·1, 33·6)
≥10% and ≥10 words	0(0·0%)	0(0·0%)	3(3·6%)	3·6(-0·4, 7·6)	3·6(-0·4, 7·6)
**9 months**	(n=83)	(n=64)	(n=77)		
≥5% and ≥5 words	4(4·8%)	6(9·4%)	14(18·2%)	13·4(3·6, 23·1)	8·8(-2·4, 20·0)
≥10% and ≥10 words	2(2·4%)	1(1·6%)	4(5·2%)	2·8(-3·2, 8·7)	3·6(-2·2, 9·4)
**12 months**	(n=84)	(n=61)	(n=74)		
≥5% and ≥5 words	4(4·8%)	9(14·8%)	18(24·3%)	19·6(8·8, 30·3)	9·6(-3·6, 22·8)
≥10% and ≥10 words	0(0·0%)	2(3·3%)	1(1·4%)	1·4(-1·3, 4·0)	-1·9(-7·1, 3·3)

Note: Complete cases only; Results obtained using normal approximation to the binomial distribution; † UC as the reference group; ‡ AC as the reference group; CI, confidence interval

1.9.16 Carer rated communication effectiveness and impact on carer's quality of life

One of the secondary objectives is to investigate the effect of the intervention on the carer rated communication effectiveness (using the first 15 questions from the CaCOAST) and impact on the carer's quality of life (using the last five questions of the CaCOAST). These are referred to as 'CaCOAST 15' and 'CaCOAST 5' respectively. It should be noted that this exploratory analysis includes only available carers who agreed to take part. In addition, the unit of randomisation was the participant and not the carer.

The unadjusted mean changes in the carer rated communication effectiveness and impact on the carer's quality of life are displayed in Figure 57 and Figure 58, respectively. Interpretation should be done in reference to the results presented in Table 23 that adjusted for baseline differences.

The mean change in carer rated communication effectiveness at six months was 6·8% in the CSLT compared to 1·0% in the UC, resulting in an adjusted mean difference in change of 4·6% (95%CI: 0·3 to 9·0) in favour of the CSLT intervention (Table 23). This small improvement in carer rated communication effectiveness in the CSLT group was similar compared to the AC group; 5·1% (95% CI: 0·5% to 9·7%). However, the long-term effects of the intervention on average change in the carer rated communication effectiveness were very small: 0·6% (95%CI: -4·4 to 5·7) and 2·7% (95%CI: -1·9 to 7·4) in favour of the CSLT compared to the UC at nine and 12 months respectively.

For the carer rated impact on their quality of life at six months, the UC decreased by 1% while the CSLT improved by 5·3%, translating to a positive adjusted mean difference in change of 5·3% (95%CI: -1·1 to 11·7) in favour of the CSLT group. However, although this seems to be of potential clinical relevance, we cannot rule out the lack of benefit and the improvement in carer's quality of life in the CSLT group compared to AC was close to zero; 0·3% (95% CI: -6·4% to 6·9%). In other words, about 5% improvement in carer's quality of life could be attributed the attention given rather than the computer therapy alone. The average effect at nine months was slightly lower compared to the six months: 4·0% (95%CI: -3·3 to 11·2) in favour of the CSLT compared to the UC. Although CIs around observed effects do not exclude the null treatment effect of zero (no difference), clinical judgements (of patients say) should be made on whether the observed treatment effects are clinically important or relevant. The interpretation should be made in consideration of the observed effect between the CSLT and AC group.

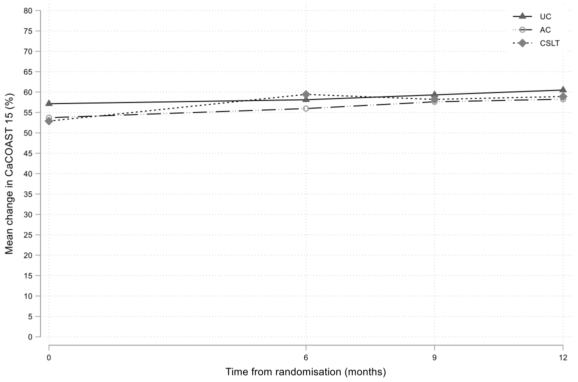

Figure 57: Average change in the carer rated communication effectiveness over time

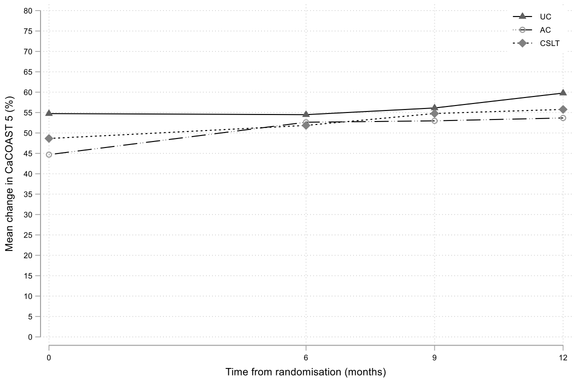

Figure 58: Average change in the carer's quality of life over time

Table 23: Intervention effect on the carer rated communication effectiveness and carer's quality of life

CaCOAST domain	UC		AC		CSLT		CSLT vs UC†		CSLT vs AC‡		AC vs UC†	
	n	Mean(SD)	n	Mean(SD)	n	Mean(SD)	Adjusted MDC (95%CI)	P-value	Adjusted MDC (95%CI)	P-value	Adjusted MDC (95%CI)	P-value
**Change in CaCOAST 15 (%) [1]**												
6 months	48	1·0(11·5)	38	2·6(11·3)	51	6·8(11·5)	4·6(0·3, 9·0)	0·038	5·1(0·5, 9·7)	0·030	-0·5(-5·2, 4·2)	0·846
9 months	43	2·5(11·7)	37	4·4(11·3)	47	5·7(13·2)	0·6(-4·4, 5·7)	0·802	0·5(-4·7, 5·6)	0·855	0·2(-5·1, 5·5)	0·953
12 months	45	3·1(11·5)	34	4·2(11·3)	44	6·6(11·6)	2·7(-1·9, 7·4)	0·244	1·9(-3·1, 6·9)	0·444	0·8(-4·2, 5·8)	0·752
**Change in CaCOAST 5 (%) [2]**												
6 months	48	-1·0(15·8)	38	7·2(14·6)	51	5·8(17·3)	5·3(-1·1, 11·7)	0·105	0·3(-6·4, 6·9)	0·940	5·0(-2·0, 12·0)	0·156
9 months	43	-0·6(18·4)	37	8·2(14·6)	47	8·5(18·7)	4·0(-3·3, 11·2)	0·279	0·2(-7·0, 7·5)	0·949	3·7(-4·0, 11·4)	0·337
12 months	45	4·0(20·1)	34	7·2(13·6)	44	8·5(19·5)	0·6(-6·3, 7·4)	0·871	3·4(-4·0, 10·8)	0·363	-2·8(-10·4, 4·8)	0·460

Note: Results based on a multiple linear regression model adjusted for baseline measures and fixed stratification factors (centre and severity of word finding); only for CC set; † UC as the reference group; ‡ AC as the reference group; MDC, mean difference in change; SD, standard deviation; CI, confidence interval; **1** high positive scores indicate improved carer rated communication effectiveness; **2** high positive scores indicate improved impact on carer's quality of life

1.9.17 COAST and CaCOAST subitems

In the prior CACTUS pilot trial, the participants and carers were interviewed about the benefits of computer therapy and seven themes that mapped on to eight items of the COAST and CaCOAST. In this section, results exploring whether qualitative findings from the pilot trial are supported by quantitative data are presented. Table 24 and Table 25 summarise the distribution of the eight items of interest of the COAST and CaCOAST with median differences in ratings and 95% CI between the CSLT versus UC and CSLT versus AC. In general, the distribution of the rating of the items appears to be similar and the quantitative evidence does not seem to support the qualitative findings from the pilot trial.

Table 24: Intervention effect on the subitems of the COAST at 6, 9, and 12 months

Assessment	COAST items	UC		AC		CSLT		CSLT vs UC†	CSLT vs AC‡
		n	Median(IQR)	n	Median(IQR)	n	Median(IQR)	Median Diff (95%CI)	Median Diff (95% CI)
**6 months**	Item 3: Chat with someone you know well	86	3·0 (2·0 , 4·0)	71	3·0(2·0 , 4·0)	83	3·0(2·0 , 4·0)	0·0(-0·0 , 0·0)	0·0(-0·2 , 0·2)
	Item 4: Short conversation with unfamiliar person	86	2·0 (1·0 , 3·0)	70	2·0(1·0 , 3·0)	83	2·0(2·0 , 3·0)	0·0(-0·1 , 0·1)	0·0(-0·1 , 0·1)
	Item 6: Make self-understood in longer sentences	84	2·0 (1·5 , 3·0)	70	2·0(1·0 , 3·0)	83	2·0(1·0 , 2·0)	0·0(-0·2 , 0·2)	0·0(-0·3 , 0·3)
	Item 11: How well can you write	85	2·0 (1·0 , 3·0)	71	2·0(1·0 , 2·0)	83	2·0(1·0 , 2·0)	0·0(-0·7 , 0·7)	0·0(-1·1 , 1·1)
	Item 15: Confidence	84	2·0 (1·0 , 2·0)	69	2·0(2·0 , 3·0)	83	2·0(2·0 , 3·0)	0·0(-0·1 , 0·1)	0·0(-0·1 , 0·1)
	Item 16: Family	83	3·0 (2·0 , 3·0)	65	2·0(2·0 , 3·0)	82	3·0(2·0 , 3·0)	0·0(-1·1 , 1·1)	1·0(-0·3 , 2·3)
	Item 17: Social life	83	2·0 (2·0 , 3·0)	69	2·0(2·0 , 3·0)	80	2·0(2·0 , 3·0)	0·0(-0·7 , 0·7)	0·0(-0·7 , 0·7)
	Item 20· Quality of life	83	3·0 (2·0 , 3·0)	70	3·0(2·0 , 3·0)	82	2·0(2·0 , 3·0)	-1·0(-2·1 , 0·1)	-1·0(-2·0 , 0·0)
**9 months**	Item 3: Chat with someone you know well	82	3·0 (2·0 , 3·0)	63	3·0(2·0 , 3·0)	76	3·0(2·0 , 3·0)	0·0(-0·1 , 0·1)	0·0(-0·3 , 0·3)
	Item 4: Short conversation with unfamiliar person	82	2·0 (1·0 , 3·0)	63	2·0(2·0 , 3·0)	75	2·0(1·0 , 3·0)	0·0(-0·1 , 0·1)	0·0(-0·1 , 0·1)
	Item 6: Make self-understood in longer sentences	82	2·0 (1·0 , 2·0)	63	2·0(2·0 , 3·0)	77	2·0(1·0 , 3·0)	0·0(-0·2 , 0·2)	0·0(-0·2 , 0·2)
	Item 11: How well can you write	81	2·0 (1·0 , 3·0)	63	2·0(1·0 , 3·0)	74	2·0(1·0 , 3·0)	0·0(-0·8 , 0·8)	0·0(-0·8 , 0·8)
	Item 15: Confidence	81	2·0 (2·0 , 2·0)	63	2·0(2·0 , 3·0)	74	2·0(2·0 , 3·0)	0·0(-0·0 , 0·0)	0·0(-0·3 , 0·3)
	Item 16: Family	82	3·0 (2·0 , 3·0)	62	3·0(2·0 , 3·0)	74	3·0(2·0 , 3·0)	0·0(-1·1 , 1·1)	0·0(-1·2 , 1·2)
	Item 17: Social life	81	2·0 (2·0 , 3·0)	61	2·0(2·0 , 3·0)	74	2·0(2·0 , 3·0)	0·0(-0·6 , 0·6)	0·0(-0·8 , 0·8)
	Item 20· Quality of life	81	3·0 (2·0 , 3·0)	63	3·0(2·0 , 3·0)	74	3·0(2·0 , 3·0)	0·0(-1·0 , 1·0)	0·0(-1·2 , 1·2)
**12 months**	Item 3: Chat with someone you know well	84	3·0 (3·0 , 4·0)	61	3·0(3·0 , 4·0)	74	3·0(2·0 , 4·0)	0·0(· , ·) [a]	0·0(· , ·) [a]
	Item 4: Short conversation with unfamiliar person	82	2·0 (2·0 , 3·0)	61	2·0(2·0 , 3·0)	73	2·0(2·0 , 3·0)	0·0(-0·5 , 0·5)	0·0(-0·6 , 0·6)
	Item 6: Make self-understood in longer sentences	83	2·0 (2·0 , 3·0)	61	2·0(1·0 , 3·0)	74	2·0(1·0 , 3·0)	0·0(-0·1 , 0·1)	0·0(-0·1 , 0·1)
	Item 11: How well can you write	83	2·0 (1·0 , 3·0)	61	2·0(1·0 , 3·0)	74	2·0(1·0 , 3·0)	0·0(-0·2 , 0·2)	0·0(-0·3 , 0·3)
	Item 15: Confidence	82	2·0 (2·0 , 3·0)	61	2·0(2·0 , 3·0)	74	2·0(2·0 , 3·0)	0·0(-0·9 , 0·9)	0·0(-1·1 , 1·1)
	Item 16: Family	80	3·0 (2·0 , 3·0)	57	3·0(2·0 , 3·0)	73	3·0(2·0 , 3·0)	0·0(-0·8 , 0·8)	0·0(-1·0 , 1·0)
	Item 17: Social life	81	2·0 (2·0 , 3·0)	59	2·0(2·0 , 3·0)	72	2·5(1·0 , 3·0)	0·5(-0·7 , 1·7)	0·5(-0·6 , 1·6)
	Item 20· Quality of life	81	3·0 (2·0 , 3·0)	58	3·0(2·0 , 3·0)	74	3·0(2·0 , 3·0)	0·0(-0·9 , 0·9)	0·0(-1·0 , 1·0)

Note: † UC as the reference group; ‡ AC as the reference group; IQR, interquartile range; CI, confidence interval; Diff, difference; [a] could not estimate CI as distributions overlap perfectly

Table 25. Intervention effect on the subitems of the CaCOAST at 6, 9, and 12 months

Assessment	CaCOAST Items	UC		AC		CSLT		CSLT vs UC†	CSLT vs AC‡
		n	Median(IQR)	n	Median(IQR)	n	Median(IQR)	Median Diff (95%CI)	Median Diff (95%CI)
**6 months**	Item 3: Chat with someone they know well	48	2·5(2·0, 3·0)	38	2·0(2·0, 3·0)	53	3·0(2·0, 3·0)	0·5(-0·8, 1·8)	1·0(-0·3, 2·3)
	Item 4: Chat with an unfamiliar person	49	2·0(1·0, 3·0)	38	2·0(1·0, 3·0)	53	2·0(1·0, 3·0)	0·0(-0·8, 0·8)	0·0(-1·0, 1·0)
	Item 6: Use longer sentences	49	2·0(1·0, 2·0)	38	1·0(1·0, 2·0)	53	2·0(1·0, 3·0)	0·0(-1·2, 1·2)	1·0(-0·2, 2·2)
	Item 11: Write	49	2·0(1·0, 3·0)	38	1·5(1·0, 2·0)	53	1·0(1·0, 3·0)	-1·0(-2·3, 0·3)	-0·5(-1·9, 0·9)
	Item 15: Their confidence communicating	49	2·0(1·0, 3·0)	38	2·0(1·0, 2·0)	53	2·0(1·0, 3·0)	0·0(-0·7, 0·7)	0·0(-0·7, 0·7)
	Item 16: Family life	49	3·0(2·0, 3·0)	38	2·0(2·0, 3·0)	53	2·0(2·0, 3·0)	-1·0(-2·1, 0·1)	0·0(-1·0, 1·0)
	Item 17: Social life	49	2·0(1·0, 3·0)	38	2·0(2·0, 3·0)	52	2·0(1·0, 3·0)	0·0(-0·7, 0·7)	0·0(-0·3, 0·3)
	Item 20: Quality of life	49	3·0(2·0, 3·0)	38	2·0(2·0, 3·0)	53	2·0(2·0, 3·0)	-1·0(-2·2, 0·2)	0·0(-1·2, 1·2)
**9 months**	Item 3: Chat with someone they know well	44	3·0(2·0, 3·0)	37	3·0(2·0, 3·0)	49	3·0(2·0, 3·0)	0·0(-1·0, 1·0)	0·0(-0·9, 0·9)
	Item 4: Chat with an unfamiliar person	44	2·0(1·0, 2·0)	37	2·0(1·0, 3·0)	49	2·0(1·0, 3·0)	0·0(-0·6, 0·6)	0·0(-0·9, 0·9)
	Item 6: Use longer sentences	44	2·0(1·0, 3·0)	37	2·0(1·0, 2·0)	49	2·0(1·0, 3·0)	0·0(-0·5, 0·5)	0·0(-0·8, 0·8)
	Item 11: Write	44	1·0(1·0, 3·0)	37	2·0(1·0, 2·0)	49	2·0(1·0, 2·0)	1·0(-0·2, 2·2)	0·0(-1·2, 1·2)
	Item 15: Their confidence communicating	44	2·0(2·0, 2·5)	37	2·0(2·0, 2·0)	49	2·0(1·0, 3·0)	0·0(-0·1, 0·1)	0·0(-0·1, 0·1)
	Item 16: Family life	44	2·0(2·0, 3·0)	37	2·0(2·0, 3·0)	49	2·0(2·0, 3·0)	0·0(-1·2, 1·2)	0·0(-1·2, 1·2)
	Item 17: Social life	44	2·0(2·0, 3·0)	37	2·0(1·0, 3·0)	49	2·0(1·0, 3·0)	0·0(-0·2, 0·2)	0·0(-0·2, 0·2)
	Item 20: Quality of life	44	3·0(2·0, 3·0)	37	2·0(2·0, 3·0)	49	2·0(2·0, 3·0)	-1·0(-2·1, 0·1)	0·0(-1·2, 1·2)
**12 months**	Item 3: Chat with someone they know well	46	3·0(2·0, 3·0)	34	3·0(2·0, 3·0)	45	3·0(2·0, 4·0)	0·0(-0·4, 0·4)	0·0(-1·0, 1·0)
	Item 4: Chat with an unfamiliar person	46	2·0(2·0, 3·0)	34	2·0(1·0, 3·0)	45	2·0(1·0, 2·0)	0·0(-0·7, 0·7)	0·0(-0·8, 0·8)
	Item 6: Use longer sentences	46	2·0(1·0, 3·0)	34	2·0(1·0, 2·0)	45	2·0(1·0, 3·0)	0·0(-0·8, 0·8)	0·0(-1·1, 1·1)
	Item 11: Write	46	2·0(1·0, 3·0)	34	1·0(1·0, 2·0)	45	1·0(1·0, 2·0)	-1·0(-1·8, -0·2)	0·0(-1·1, 1·1)
	Item 15: Their confidence communicating	46	2·0(1·0, 2·0)	34	2·0(1·0, 2·0)	45	2·0(2·0, 3·0)	0·0(-0·2, 0·2)	0·0(-0·2, 0·2)
	Item 16: Family life	46	3·0(2·0, 3·0)	34	2·0(2·0, 3·0)	45	2·0(2·0, 3·0)	-1·0(-2·2, 0·2)	0·0(-0·8, 0·8)
	Item 17: Social life	46	2·0(2·0, 3·0)	34	2·0(1·0, 3·0)	45	2·0(2·0, 3·0)	0·0(-1·0, 1·0)	0·0(-0·8, 0·8)
	Item 20: Quality of life	46	3·0(2·0, 3·0)	34	2·5(2·0, 3·0)	45	3·0(2·0, 3·0)	0·0(-1·0, 1·0)	0·5(-0·8, 1·8)

Note: † UC as the reference group; ‡ AC as the reference group; IQR: interquartile range; CI, confidence interval; Diff, difference

1.9.18 Safety results

The final secondary objective is to identify any possible adverse events. To address this objective, this section reports the incidences of the AEs, SAEs, and negative effects of the computer therapy using treatment as received principle described in Section 1.1.6.6 including sensitivity analysis results using the strict ITT principle (treatment-as-randomised).

1.9.18.1 The incidences of adverse events

Of the 97 participants randomised to receive the CSLT, computer therapy use was not recorded in 12 participants (12·4%); three of whom died before the 6-month follow-up. Only one participant who was allocated to AC died before the 6-month follow-up and was never send a puzzle book. Therefore, according to treatment-as-received principle, these 13 participants were technically treated as received the UC alone. As a result, the denominators for the treatment-as-received analysis include 101 in the UC, 79 in the AC, and 85 in the CSLT. The incidences of AEs using treatment-as-received principle are summarised in Table 26. The proportion of participants who experienced any AE was 61 (71·8%) in the CSLT, 50 (63·3%) in the AC, and 70 (61·4%) in the UC. On average, the incidences of AEs per participant per person-year of follow-up was 2·18, 1·79, and 1·87 in the CLST, AC, and UC respectively. This indicates a slight increase in all AEs in the CSLT with an IRR of 1·16 (95%CI: 0·83 to 1·62) and 1·22 (95%CI: 0·85 to 1·77) compared to the UC and AC, respectively. However, there was no sufficient evidence to suggest an increased risk of experiencing AEs in the CSLT compared to AC or UC, as supported by CIs that includes a risk ratio of 1. Although the incidences of experiencing any fits or seizures were not common, the risk was more than three times in the CSLT compared to either the UC or AC. The incidences of AEs by category are detailed in Table 26.

For sensitivity analysis, Table 27 summarises the incidences of AEs using the strict ITT principle (treatment-as-randomised). In summary, the interpretation of the results is consistent with the treatment-as-received results presented in Table 26.

Table 26: Incidences of AEs (treatment-as-received)

AE classification	UC (n=114)	AC (n= 79)	CSLT (n= 85)	IRR (95%CI) CSLT vs UC	CSLT vs AC	AC vs UC
**Had experienced at least one AE**	70(61·4%)	50(63·3%)	61(71·8%)			
**Repeated AEs**						
**All AEs**						
Total events/person yrs	200/105·4	136/74·7	185/84·7			
IR/ person year (95%CI)	1·87(1·47, 2·38)	1·79(1·38, 2·31)	2·18(1·72, 2·77)	1·16 (0·83, 1·62)	1·22 (0·85, 1·77)	0·95 (0·67, 1·35)
**Felt more tired than usual**						
Total events/person yrs	125/105·4	77/74·7	114/84·7			
IR/ person year (95%CI)	1·18(0·82, 1·70)	1·01(0·70, 1·45)	1·32(0·95, 1·84)	1·12(0·69, 1·83)	1·32(0·81, 2·14)	0·85(0·51, 1·42)
**Had any fits or seizures**						
Total events/person yrs	18/105·4	13/74·7	47/84·7			
IR/ person year (95%CI)	0·16(0·06, 0·44)	0·17(0·08, 0·37)	0·57(0·29, 1·12)	3·48(1·05, 11·57)	3·41(1·21, 9·62)	1·02(0·29, 3·63)
**Had worsening vision or visual difficulties**						
Total events/person yrs	47/105·4	34/74·7	71/84·7			
IR/ person year (95%CI)	0·42(0·22, 0·80)	0·44(0·25, 0·79)	0·83(0·51, 1·36)	1·95(0·87, 4·37)	1·89(0·89, 4·05)	1·03(0·43, 2·44)
**Had increasing number or increasing severity of headaches**						
Total events/person yrs	46/105·4	25/74·7	52/84·7			
IR/ person year (95%CI)	0·43(0·23, 0·81)	0·31(0·13, 0·78)	0·58(0·34, 1·01)	1·36(0·59, 3·11)	1·84(0·64, 5·30)	0·74(0·24, 2·21)
**Had any accidents (e.g. falls) or injuries**						
Total events/person yrs	90/105·4	51/74·7	48/84·7			
IR/ person year (95%CI)	0·87(0·58, 1·30)	0·66(0·42, 1·04)	0·56(0·35, 0·89)	0·64(0·35, 1·19)	0·85(0·45, 1·61)	0·76(0·42, 1·39)
**Reported any other negative effects or events**						
Total events/person yrs	64/105·4	29/74·7	44/84·7			
IR/ person year (95%CI)	0·60(0·40, 0·92)	0·38(0·21, 0·68)	0·55(0·35, 0·86)	0·91(0·49, 1·68)	1·44(0·69, 3·00)	0·63(0·31, 1·28)

Note: Results from a negative binomial regression model; IR, incidence rate; IRR, incidence rate ratio; CI, confidence interval; yrs, years; † UC as the reference group; ‡ AC as the reference group

Table 27: Incidences of AEs (treatment-as-randomised)

AE classification	UC (n=101)	AC (n= 80)	CSLT (n= 97)	IRR (95%CI) CSLT vs UC†	CSLT vs AC‡	AC vs UC†
**Had experienced at least one AE**	62(61·4%)	50(62·5%)	69(71·1%)			
**Repeated AEs**						
**All AEs**						
Total events/person yrs	186/97·1	136/74·8	199/92·9			
IR/ person year (95%CI)	1·90(1·46, 2·48)	1·78(1·38, 2·30)	2·13(1·71, 2·65)	1·11 (0·80, 1·56)	1·19 (0·83, 1·71)	0·93 (0·65, 1·34)
**Felt more tired than usual**						
Total events/person yrs	122/97·1	77/74·8	117/92·9			
IR/ person year (95%CI)	1·28(0·84, 1·93)	1·00(0·69, 1·47)	1·21(0·85, 1·74)	0·95(0·56, 1·61)	1·21(0·69, 2·14)	0·78(0·45, 1·38)
**Had any fits or seizures**						
Total events/person yrs	15/97·1	13/74·8	50/92·9			
IR/ person year (95%CI)	0·15(0·04, 0·52)	0·17(0·07, 0·41)	0·53(0·24, 1·19)	3·57(1·01, 12·67)	3·22(0·83, 12·42)	1·11(0·27, 4·54)
**Had worsening vision or visual difficulties**						
Total events/person yrs	46/97·1	34/74·8	72/92·9			
IR/ person year (95%CI)	0·45(0·21, 0·97)	0·44(0·23, 0·84)	0·76(0·42, 1·36)	1·69(0·69, 4·13)	1·74(0·67, 4·57)	0·97(0·37, 2·56)
**Had increasing number or increasing severity of headaches**						
Total events/person yrs	44/97·1	25/74·8	54/92·9			
IR/ person year (95%CI)	0·46(0·21, 1·00)	0·31(0·11, 0·91)	0·54(0·27, 1·09)	1·17(0·39, 3·52)	1·71(0·52, 5·62)	0·69(0·21, 2·26)
**Had any accidents (e.g. falls) or injuries**						
Total events/person yrs	86/97·1	51/74·8	52/92·9			
IR/ person year (95%CI)	0·92(0·58, 1·45)	0·66(0·41, 1·06)	0·55(0·35, 0·87)	0·60(0·32, 1·13)	0·83(0·42, 1·66)	0·72(0·37, 1·40)
**Reported any other negative effects or events**						
Total events/person yrs	59/97·1	29/74·8	49/92·9			
IR/ person year (95%CI)	0·61(0·38, 0·99)	0·38(0·20, 0·71)	0·55(0·35, 0·86)	0·90(0·46, 1·77)	1·45(0·68, 3·08)	0·62(0·30, 1·30)

Note: Results from a negative binomial regression model; IR, incidence rate; IRR, incidence rate ratio; CI, confidence interval; yrs, years; † UC as the reference group; ‡ AC as the reference group

1.9.18.2 The incidences of serious adverse events

The incidences of SAEs based on the treatment-as-received principle are summarised in Table 28. Participants who experienced any SAEs were 18 (15·8%) in the UC, 11 (13·9%) in the AC and 9 (10·6%) in the CSLT. The total number of repeated SAEs was 23, 12, and 10 in the UC, AC, and CSLT experienced over a total follow-up of 105·4, 74·7 and 84·7 person-years, respectively. The incidences of SAEs were rare across interventions such that participants would need to be followed up for a longer duration to record a single event per participant on average. For instance, the incidence rate in the CSLT group was 0·11 (95%: 0·04 to 0·19) meaning that on average a participant will need to be followed up for about ten person-years to record 1 SAE. Although the risk of experiencing any SAEs was lower in the CSLT compared to either UC or AC, there was insufficient evidence to suggest differences risk between groups. All SAEs were not or unlikely related to the trial activity and majority resulted in inpatient hospitalisation.

Table 29 presents the incidences of SAEs based on strict ITT principle (treatment as randomised). In summary, although the incidences are now slightly higher in the CSLT compared to the UC or AC, the conclusion is similar to that based on treatment as received principle (Table 28). That is, there is insufficient evidence to suggest differences in incidence rates of SAEs across interventions.

Table 28: Incidences of SAEs (treatment as received)

SAE classification	UC	AC	CSLT		IRR (95% CI)	
	(n=114)	(n= 79)	(n= 85)	CSLT vs UC†	CSLT vs AC‡	AC vs UC†
**Had experienced at least one SAE**	18(15·8%)	11(13·9%)	9(10·6%)			
**Repeated SAEs**						
Total events/person years	23/105·4	12/74·7	10/84·7			
IR/person year (95%CI)	0·23 (0·11, 0·34)	0·16 (0·06, 0·26)	0·11 (0·04, 0·19)	0·51 (0·22, 1·19)	0·72 (0·28, 1·87)	0·70 (0·31, 1·59)
**SAE resulted in inpatient hospitalisation**						
No	4	1	0			
Yes	19	11	10			
**SAE was life-threatening**						
No	14	8	7			
Yes	9	4	3			
**Expected**						
No	21	12	9			
Yes	1	0	1			
Not stated	1	0	0			
**Relationship to trial activity**						
Unlikely	1	2	2			
Unrelated	22	10	8			
**Frequency of SAE**						
Isolated	16	9	7			
Intermittent	2	1	0			
Continuous	3	0	0			
Unknown	2	2	3			
**Intensity of SAE**						
Mild	3	1	1			
Moderate	12	6	5			
Severe	8	4	4			
Missing	0	1	0			
**Outcome of SAE**						
Recovered	10	3	4			
Improved	3	3	1			
Ongoing	5	5	3			
Death	5	1	2			
**Action taken**						
None	19	10	9			
Reduce intervention	1	0	0			
Intervention withdrawal	1	1	1			
Other	2	1	0			

Note: Results from a negative binomial regression model; IR, incidence rate, IRR, incidence rate ratio; CI, confidence interval; † UC as the reference group; ‡ AC as the reference group;

Table 29: Incidences of SAEs (treatment-as-randomised)

SAE classification	UC	AC	CSLT	IRR (95%CI)		
	(n=101)	(n= 80)	(n= 97)	CSLT vs UC†	CSLT vs AC‡	AC vs UC†
**Had experienced at least one SAE**	13(12·9%)	11(13·8%)	14(14·4%)			
**Repeated SAEs**						
Total events/person yrs	15/97·1	12/74·8	18/92·9			
IR/person year (95%CI)	0·16 (0·06, 0·25)	0·16 (0·05, 0·26)	0·19 (0·09, 0·30)	1·24 (0·56, 2·76)	1·23 (0·52, 2·88)	1·01 (0·42, 2·43)
**SAE resulted in inpatient hospitalisation**						
No	3	1	1			
Yes	12	11	17			
**SAE was life-threatening**						
No	8	8	13			
Yes	7	4	5			
**Expected**						
No	13	12	17			
Yes	1	0	1			
Not stated	1	0	0			
**Relationship to trial activity**						
Unlikely	0	2	3			
Unrelated	15	10	15			
**Frequency of SAE**						
Isolated	11	9	12			
Intermittent	2	1	0			
Continuous	1	0	2			
Unknown	1	2	4			
**Intensity of SAE**						
Mild	2	1	2			
Moderate	7	6	10			
Severe	6	4	6			
Missing	0	1	0			
**Outcome of SAE**						
Recovered	5	3	9			
Improved	3	3	1			
Ongoing	3	5	5			
Death	4	1	3			
**Action taken**						
None	13	10	15			
Reduce intervention	0	0	1			
Intervention withdrawal	1	1	1			
Other	1	1	1			

Note: Results from a negative binomial regression model; IR, incidence rate; IRR, incidence rate ratio; CI, confidence interval; † UC as the reference group; ‡ AC as the reference group

1.9.18.3 Negative effects of computer therapy

Table 30 summarises negative effects of computer therapy among those that used the computer. Of the 85 participants who used computer therapy, 23 (27·1%) felt that the computer practice made them overtired and anxious or worried, translating to an average incident rate of one episode per person-year. The incidences of negative effects of computer therapy on causing headaches and affecting eyes were rare. The results based on treatment as received (Table 30) and treatment as randomised (Table 31) principles are very similar.

Table 30: Negative effect of computer therapy (only those who used the computer)

		CSLT (N=85)	
**Has the computer practice:**	**n(%)**	**Total events/person years**	**IR/ person year (95%CI)**
Made you feel overtired?	23(27·1%)	50/42·1	1·18(0·74, 1·90)
Affected your eyes?	11(12·9%)	17/42·1	0·40(0·21, 0·78)
Given you headaches?	5(5·9%)	6/42·1	0·14(0·06, 0·36)
Made you feel anxious/worried?	23(27·1%)	42/42·1	0·99(0·63, 1·56)

Note: Results from a negative binomial regression model; IR: incidence rate; CI: confidence interval.

Table 31: Negative effect of the computer therapy (treatment as randomised)

		CSLT (N=97)	
**Has the computer practice:**	**n(%)**	**Total events/person years**	**IR/ person year (95%CI)**
Made you feel over tired?	26(26·8%)	53/47·1	1·14(0·73, 1·78)
Affected your eyes?	11(11·3%)	17/47·1	0·36(0·18, 0·70)
Given you headaches?	5(5·2%)	6/47·1	0·13(0·05, 0·32)
Made you feel anxious/worried?	26(26·8%)	45/47·1	0·97(0·63, 1·49)

Note: Results from a negative binomial regression model; IR, incidence rate; CI, confidence interval.

1.9.19 Post hoc PP analysis of the co-primary and key secondary outcomes

On the 7th March 2018, the Trial Management Group discussed the final trial results in detail. The group noted that only 14 (17·7%) of the AC met the predefined PP inclusion criteria. Following the discussion, the group agreed that the predefined PP proxy of being sent six puzzle books in six months was over ambitious and inconsistent with expectations of PP for the CSLT. To align the puzzle book PP classification in the AC with the CSLT, the group requested post hoc PP analysis based on at least four puzzle books sent. This will only change the PP results for comparisons involving the AC. We, therefore, modified the PP classifications presented in Section 1.6.3 as follows:

1) practised computer therapy for a minimum total of 26 hrs (CSLT) or were sent at least four puzzle books (AC) within six months of randomisation (*PP1 CSLT26 AC*);
2) practised computer therapy for a minimum total of 10 hrs (CSLT or were sent at least four puzzle books (AC) within six months of randomisation (*PP2 CSLT10 AC4*);
3) practiced computer therapy for a minimum total of 26 hrs (CSLT) or were sent at least four puzzle books (AC) and contacted for at least four times (if they wish) (AC and CSLT) within six months of randomisation (*PP3 CSLT26 AC4_4*);
4) practiced computer therapy for a minimum total of ten hrs (CSLT) or were sent at least four puzzle books (AC) and contacted for at least four times (if they wish) (AC and CSLT) within six months of randomisation (*PP4 CSLT 10AC4_4*).

Based on the new AC PP classification, 48/79 (60·8%) participants were sent at least four puzzle books and contacted at least four times within six months. Table 32 presents PP sensitivity analysis results for the co-primary and secondary endpoints. In addition, the results are graphically displayed using forest plots in Figure 59 to Figure 61. In summary, the sensitivity analysis results are very similar to the ones presented in Section 1.1.9.8.3.

Table 32: Post hoc analysis - impact of adherence on the intervention effect on co-primary and key secondary endpoints

Primary and key secondary outcomes at 6m and PP set	UC		AC		CSLT		CSLT vs UC †		CSLT vs AC ‡		AC vs UC †	
	N	Mean(SD)	n	Mean(SD)	n	Mean(SD)	Adjusted MDC (95%CI)	P-value	Adjusted MDC (95%CI)	P-value	Adjusted MDC (95%C	P-value
**Co-primary outcomes**												
Change in word finding (%) [1]												
PP1 CSLT26 AC4	80	0·8(11·4)	44	2·2(7·7)	42	19·7(15·7)	18·2(14·0, 22·5)	<0·0001	16·0(11·1, 20·9)	<0·0001	2·2(-2·1, 6·5)	0·312
PP2 CSLT10 AC4	80	0·8(11·4)	44	2·2(7·7)	58	19·3(14·9)	17·9(14·1, 21·7)	<0·0001	16·1(11·6, 20·5)	<0·0001	1·9(-2·4, 6·1)	0·384
PP3 CSLT26 AC4_4	80	0·8(11·4)	44	2·2(7·7)	34	21·5(16·5)	19·7(15·0, 24·4)	<0·0001	17·5(12·1, 22·8)	<0·0001	2·2(-2·2, 6·6)	0·318
PP4 CSLT10 AC4_4	80	0·8(11·4)	44	2·2(7·7)	45	19·1(15·5)	18·0(13·8, 22·3)	<0·0001	16·0(11·0, 20·9)	<0·0001	2·1(-2·3, 6·4)	0·353
Change in functional communication [2]												
PP1 CSLT26 AC4	77	0·05(0·59)	43	0·13(0·64)	41	0·03(0·52)	-0·11(-0·33, 0·11)	0·332	-0·11(-0·37, 0·15)	0·391	0·01(-0·22, 0·23)	0·965
PP2 CSLT10 AC4	77	0·05(0·59)	43	0·13(0·64)	56	0·09(0·52)	-0·03(-0·23, 0·17)	0·758	-0·01(-0·25, 0·22)	0·909	-0·02(-0·24, 0·21)	0·881
PP3 CSLT26 AC4_4	77	0·05(0·59)	43	0·13(0·64)	33	0·06(0·55)	-0·09(-0·33, 0·16)	0·488	-0·09(-0·37, 0·20)	0·545	0·00(-0·23, 0·23)	0·998
PP4 CSLT10 AC4_4	77	0·05(0·59)	43	0·13(0·64)	44	0·08(0·53)	-0·04(-0·26, 0·17)	0·701	-0·03(-0·28, 0·23)	0·823	-0·01(-0·24, 0·21)	0·907
PP1 CSLT26 AC4												
**Key secondary outcome**												
Change in COAST (%) [3]												
PP1 CSLT26 AC4	78	3·0(12·8)	44	2·4(12·0)	42	4·7(10·6)	1·7(-2·4, 5·9)	0·411	2·5(-2·3, 7·3)	0·310	-0·8(-5·0, 3·5)	0·727
PP2 CSLT10 AC4	78	3·0(12·8)	44	2·4(12·0)	57	4·2(10·8)	1·6(-2·3, 5·4)	0·420	2·3(-2·1, 6·7)	0·309	-0·7(-4·9, 3·5)	0·731
PP3 CSLT26 AC4_4	78	3·0(12·8)	44	2·4(12·0)	34	4·5(10·7)	2·2(-2·4, 6·8)	0·345	3·0(-2·3, 8·2)	0·271	-0·7(-5·0, 3·6)	0·736
PP4 CSLT10 AC4_4	78	3·0(12·8)	44	2·4(12·0)	44	4·2(11·1)	1·7(-2·6, 6·0)	0·433	2·4(-2·6, 7·3)	0·343	-0·7(-5·0, 3·6)	0·756

Note: PP sets "PP1 CSLT26 AC4", "PP2 CSLT10 AC4", "PP3 CSLT26 AC4_4", and "PP4 CSLT10 AC4_4" are defined in Section 1.9.19 above
Results based on a multiple linear regression model adjusted for baseline measures and fixed stratification factors (centre and severity of word finding)
† UC as the reference group; ‡ AC as the reference group; MDC, mean difference in change; SD, standard deviation; CI, confidence interval
Interpretation: [1] higher scores indicate improved vocabulary of personal importance; [2] higher scores indicate improved functional communication ability in conversation, [3] higher percentage score indicates improved patient perception of communication effectiveness and its impact on their quality of life.

Analysis set	Control [n	Mean(SD)]	CSLT [n	Mean(SD)]	Adjusted MDC (95%CI)	P-value
mITT						
UC vs CSLT	86	1.1(11.2)	83	16.4(15.3)	16.2(12.7, 19.6)	<0.0001
AC vs CSLT	71	2.4(8.8)	83	16.4(15.3)	14.4(10.8, 18.1)	<0.0001
MI						
UC vs CSLT	97	1.0(12.3)	94	16.8(15.5)	16.3(12.8, 19.8)	<0.0001
AC vs CSLT	79	2.3(10.4)	94	16.8(15.5)	14.6(10.9, 18.3)	<0.0001
LI						
UC vs CSLT	88	1.0(11.1)	84	16.4(15.2)	16.2(12.7, 19.6)	<0.0001
AC vs CSLT	71	2.4(8.8)	84	16.4(15.2)	14.3(10.7, 18.0)	<0.0001
PP1 CSLT26 AC4						
UC vs CSLT	80	0.8(11.4)	42	19.7(15.7)	18.2(14.0, 22.5)	<0.0001
AC vs CSLT	44	2.2(7.7)	42	19.7(15.7)	16.0(11.1, 20.9)	<0.0001
PP2 CSLT10 AC4						
UC vs CSLT	80	0.8(11.4)	58	19.3(14.9)	17.9(14.1, 21.7)	<0.0001
AC vs CSLT	44	2.2(7.7)	58	19.3(14.9)	16.1(11.6, 20.5)	<0.0001
PP3 CSLT26 AC4_4						
UC vs CSLT	80	0.8(11.4)	34	21.5(16.5)	19.7(15.0, 24.4)	<0.0001
AC vs CSLT	44	2.2(7.7)	34	21.5(16.5)	17.5(12.1, 22.8)	<0.0001
PP4 CSLT10 AC4_4						
UC vs CSLT	80	0.8(11.4)	45	19.1(15.5)	18.0(13.8, 22.3)	<0.0001
AC vs CSLT	44	2.2(7.7)	45	19.1(15.5)	16.0(11.0, 20.9)	<0.0001

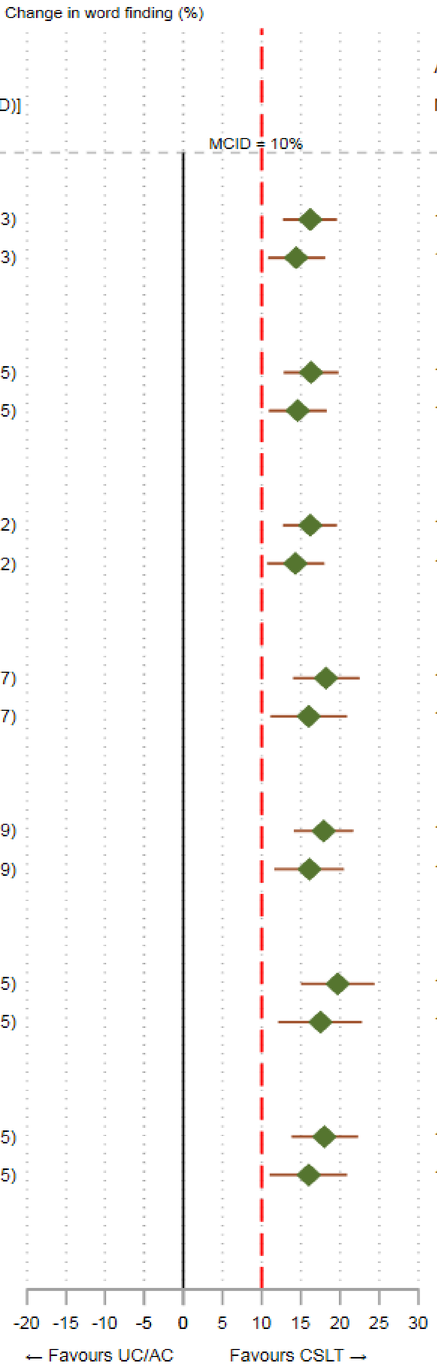

Figure 59: Impact of intervention adherence on word finding at 6 months (sensitivity analysis)

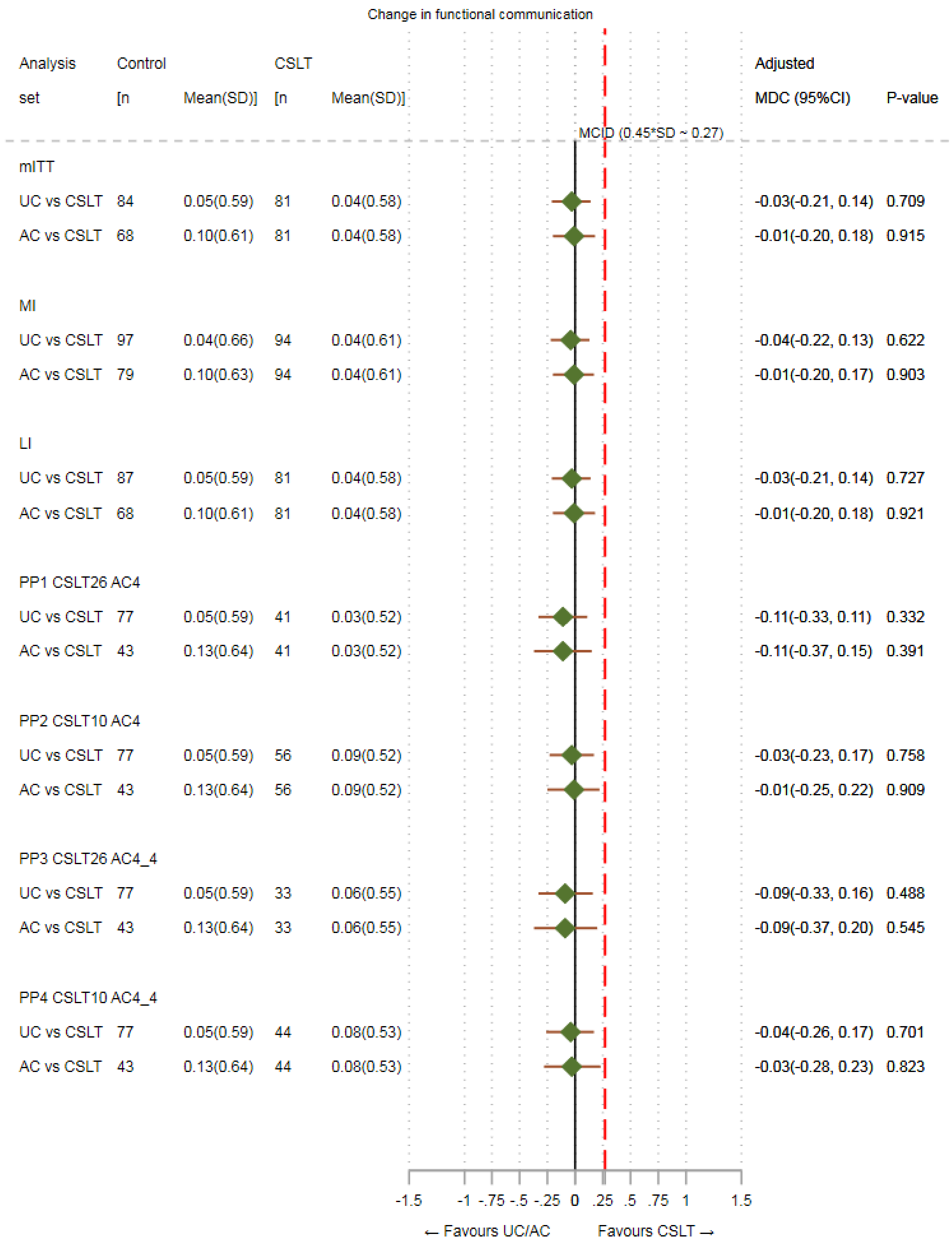

Figure 60: Impact of intervention adherence on functional communication at 6 months (sensitivity analysis)

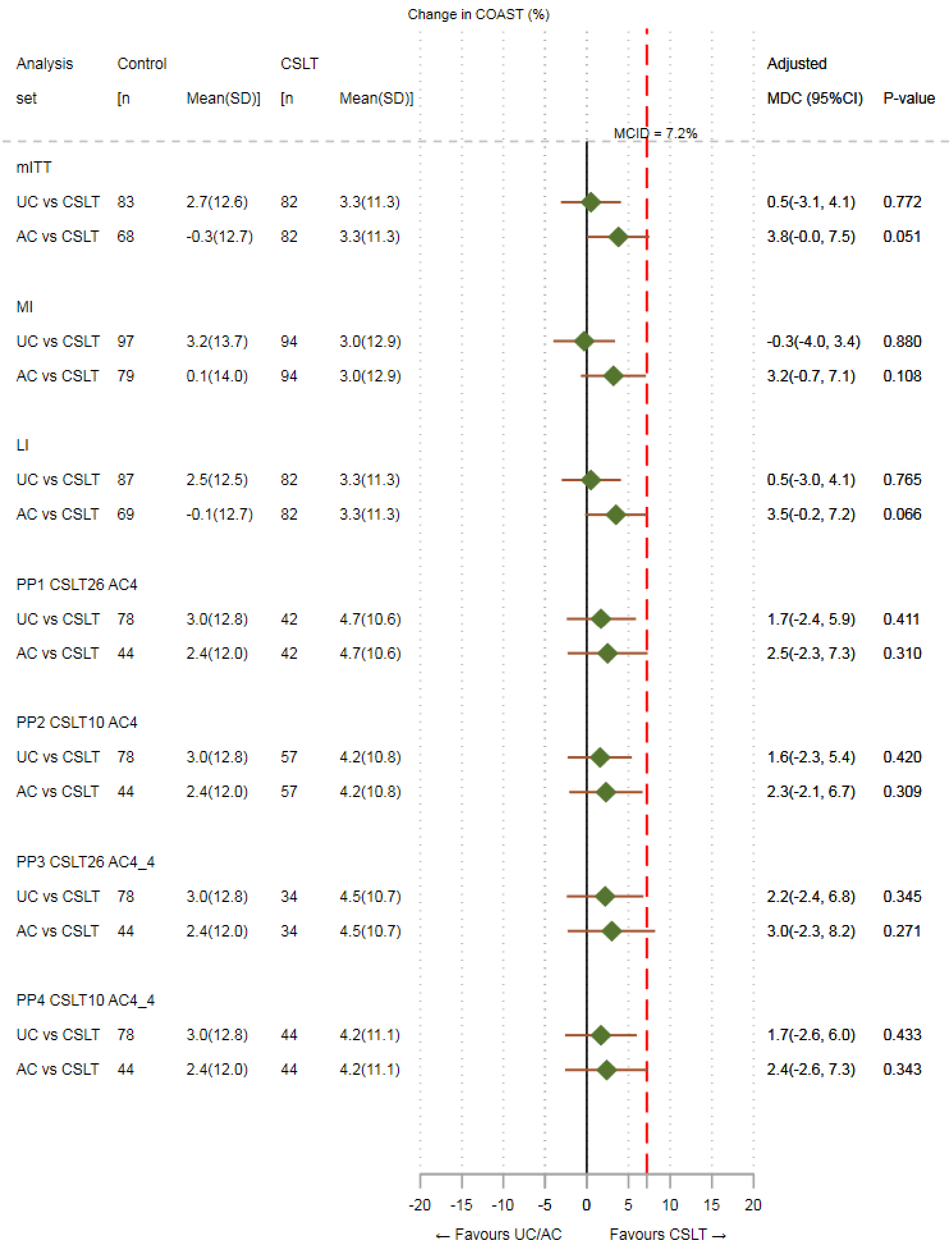

Figure 61: Impact of intervention adherence on COAST at 6 months (sensitivity analysis)

1.10 Discussion

1.10.1 Main findings

We demonstrated overwhelming evidence to support that the CSLT intervention improves the word finding ability of people with aphasia to use the vocabulary of personal importance; 16·2% (95%CI: 12·7 to 19·6; p<0·0001) compared to the UC. The results were strongly supported by a 14·4% (95%CI: 10·8 to 18·1) average improvement in word finding of personally selected words in favour of CSLT compared to the AC. Most

importantly, the short-term effect of the intervention was sustained in the long-term at nine and 12 months after therapy has begun. However, there was insufficient evidence to suggest that on average the CSLT intervention for word finding improves functional communication ability in conversation or changes in perceived social participation in daily activities and quality of life both in the short and long-term.

As for the secondary trial objectives, on average the CSLT did not result in the improved generalisation of treatment to the finding of untreated words The intervention resulted in small short-term improvement in carer's perception of communication effectiveness which could be of potential clinical relevance; 4·6% (95% CI: 0·3% to 9·0%). However, the intervention failed to translate into a meaningful impact on the carer's reported quality of life based on the perceptions of the carers who were available and agreed to take part in the study.

As for safety objectives, the incidences of AEs and SAE were comparably similar across interventions and were generally rare. The most negative effects of the computer therapy recorded by 27% of the CSLT participants were feelings of overtired and anxiety.

1.10.2 Strengths and limitations

This study was an adequately powered RCT addressing multiple key objectives while controlling for chances of making false positive conclusions about the beneficial effects of the intervention. This is a multi-centre study covering SLT sites across the UK so results are generalisable to a wider setting.

We included an active 'attention' comparator in addition to the usual care to facilitate the interpretation of the results. In addition, we investigated the short and long-term effects of the interventions, together with the impact of attrition and adherence to the intervention effects to support the robust interpretation of the results. Although the trial was single-blinded due to the nature of the intervention, outcome assessors were blinded to treatment allocation to minimise the potential to introduce operation bias in outcome assessments. We put in place adequate measures and processes to deal with cases of unblinding of outcome assessors as well as reporting of unblinding cases of outcome assessors for transparency and to enable readers to make informed judgements about the robustness of trial results. The Trial Statistician was blinded to treatment allocation until data lock and analysis was guided by pre-defined approached documented in accessible SAPs that were signed off before data lock. Few post hoc analyses that were performed at the request of the CI following the disclosure of the results from the predefined analysis are highlighted in this report and document for an audit trail.

As a limitation, this trial was single-blinded due to the nature of interventions, used subjective outcomes, and the unblinding cases of outcome assessors were slightly higher in the CSLT and AC compared to UC and most cases occurred prior to 6-month assessment. However, cases are very low relative to the overall number of assessments done and only one occurred at 12 months. We recorded low adherence to the AC intervention with only 18% meeting the pre-defined adherence criteria and 61% meeting a post hoc adherence criterion. We observed moderate adherence to the CSLT intervention; 64% and 46% practiced the computer therapy for at least ten and 26 hours within six months of randomisation and computer therapy use was not recorded in 12% of participants. Therefore, PP results should be interpreted with some caution due to the reduced number of participants included in related analyses. Nevertheless, PP results including those from post hoc revised adherence criteria to AC intervention were very consistent with the main results. Exploring the predictive factors of adherence and applying instrumental variable regression to estimate complier average causal effect of the intervention in an area worth exploring further.

There was a small imbalance in the total number of participants randomised to the AC compared to the UC and CSLT despite the use of stratified block randomisation. There was no subversion of the randomisation system and procedure. We strongly believe this occurred by chance due to a large number of strata relative to the small number of participants randomised within each SLT site. The termination of recruitment after 278 rather than the planned 285 may have aided the imbalance. Nevertheless, the trial had more power than planned for the CSLT versus UC comparison and adequate power for the CSLT versus AC comparison in order to address key research objectives. In addition, the characteristics and demographics of participants were broadly similar across interventions and the main results were consistent regardless of the potential confounders adjusted for in the statistical analysis model.

2 Baseline characteristics of multiple imputation population

Table 33. Baseline characteristics of multiple imputation analysis population (N=270)

Characteristic	UC (n=97)	AC (n=79)	CSLT (n=94)
**Sex**			
Male	60(61·9%)	49(62·0%)	55(58·5%)
Female	37(38·1%)	30(38·0%)	39(41·5%)
**Age at consent (years)**			
Mean(SD)	65·6(13·1)	64·8(13·1)	65·6(12·7)
Median(IQR)	66·6(55·8, 74·7)	66·2(54·6, 74·9)	66·1(55·5, 75·5)
Min, Max	23·1, 91·8	30·4, 88·7	34·1, 89·2
**CAT comprehension severity** [a]			
Within normal limits (WNL)	20(20·6%)	13(16·5%)	17(18·1%)
Mild	50(51·5%)	36(45·6%)	43(45·7%)
Moderate	24(24·7%)	24(30·4%)	29(30·9%)
Severe	3(3·1%)	6(7·6%)	5(5·3%)
**Severity of word finding difficulty** [b]			
Mild	40(41·2%)	38(48·1%)	41(43·6%)
Moderate	33(34·0%)	19(24·1%)	28(29·8%)
Severe	24(24·7%)	22(27·8%)	25(26·6%)
**Type of aphasia**			
Anomic	39(40·2%)	22(27·8%)	35(37·2%)
Non-fluent (e·g· Broca's)	40(41·2%)	29(36·7%)	38(40·4%)
Mixed non-fluent	13(13·4%)	21(26·6%)	15(16·0%)
Fluent (e·g· Wernicke's)	5(5·2%)	7(8·9%)	6(6·4%)
**Type of stroke**			
Infarct	79(81·4%)	64(81·0%)	69(73·4%)
Haemorrhage	14(14·4%)	7(8·9%)	14(14·9%)
Not known	9(9·3%)	8(10·1%)	11(11·7%)
**Time post-stroke (years)**			
Mean(SD)	2·8(2·7)	3·4(4·6)	2·8(2·9)
Median(IQR)	1·9(0·9, 3·8)	1·9(1·0, 4·3)	1·8(0·7, 3·6)
Min, Max	0·3, 15·7	0·4, 36·1	0·4, 12·7
**Word finding ability (%)**[c]	(n=97)	(n=79)	(n=94)
Mean(SD)	42·8(18·1)	41·4(20·7)	43·2(19·0)
Median(IQR)	44·0(30·0, 57·0)	37·5(23·5, 59·0)	43·8(30·0, 57·5)
Min, Max	5·0, 85·0	9·0, 82·0	4·5, 86·0
**Functional conversation (TOMs)**[d]	(n=96)	(n=78)	(n=93)
Mean(SD)	3·1(1·0)	2·7(1·0)	2·9(1·2)
Median(IQR)	3·0(2·5, 4·0)	2·5(2·0, 3·5)	3·0(2·0, 4·0)
Min, Max	0·5, 5·0	1·0, 4·5	0·5, 5·0

COAST (%)[e]	(n=94)	(n=79)	(n=89)
Mean(SD)	59·9(13·1)	60·0(13·8)	58·2(13·6)
Median(IQR)	61·3(52·5, 68·8)	60·0(48·8, 68·8)	57·5(48·8, 68·8)
Min, Max	26·3, 86·3	26·3, 96·3	26·3, 87·5

Note: [a] Derived from CAT comprehension of sentences test scores out of a total of 32 (within normal limits, WNL 27 to 32; mild 18 to 26, moderate 9 to 17, severe 0 to 8); [b] Derived from CAT Object Naming out of 48 (mild 31 to 43, moderate 18 to 30, severe 5 to 15); [c] word finding ability of personally chosen words (%) based on the Personal Vocabulary Naming Test; [d] TOMs rating score ranges from 0 to 5, with higher scores meaning improved functional communication; [e] higher score indicates positive self-perceived communication and impact patient's quality of life; SD, standard deviation; IQR, interquartile range; min, minimum; max, maximum; UC, usual care; AC, attention control; CSLT, computerised speech language therapy.

3 Carer outcomes

The carer perception of communication effectiveness and the impact on the carer quality of life were explored using the self-reported CarerCOAST questionnaire. Small differences were seen in carer's perception of communication effectiveness (4·6%) in favour of CSLT compared to UC (95% CI: 0·3% to 9·0%) at six months, and 5·1% in favour of CSLT compared to AC (95% CI: 0·5% to 9·7%) but the differences were not maintained at nine and 12 months (see Table 23). Improvement in carer's quality of life in the CSLT group compared to UC was 5·3% (95% CI: -1·1% to 11·7%), However when compared to AC the improvement with CSLT was close to zero; 0·3% (95% CI: -6·4% to 6·9%) suggesting any benefits in quality of life for carers were likely to be due to the additional attention received in CSLT and AC.

4 Procedures to maintain blinding of outcome assessors

SLTs providing the intervention were asked not to disclose baseline case report forms, not to openly discuss participants with colleagues in open plan offices, and to remind their participants not to discuss their activities on the trial with any other SLTs they may come into contact with. When outcome assessors contacted participants and conducted their assessments they were advised to remind participants that their activity on the trial was 'a secret'. It was possible that during a conversation with the participant or carer, outcome assessors could be unblinded. If this occurred on the telephone, before the assessment took place, then the assessment was carried out by a different blinded outcome assessor. In the event of unblinding during an assessment, the next assessment was carried out by a different blinded assessor. All sites had a minimum of two trained SLT outcome assessors that were blinded to the allocation to allow for unblinding issues. If the treatment arm allocation was disclosed during an assessment, then the outcome assessor would complete an unblinding form recording what they believed the participant's intervention allocation to be with descriptive summaries of circumstances surrounding the unblinding to enable reporting of the relative success of blinding procedures.

5 Short report on randomisation allocation

5.1 Background

The Big Cactus treatment allocation was delivered using stratified block randomisation. The schedule comprised randomly ordered blocks of size three and six, stratified by site and severity of word retrieval. These strata were pre-specified at the proposal development phase to assist balance of characteristics across arms as they were expected to be associated with different outcomes. Severity was classified as mild, moderate or severe on the basis of the Comprehensive Aphasia Test (CAT) and 20 sites were anticipated, although 21 sites eventually recruited into the trial.

A sample size of 285 was planned, split equally with 95 per arm. In total 278 participants were included: 97 were randomised to computerised therapy, 101 to usual care and 80 to attention control, giving a marked imbalance of 21 between the largest and smallest of the treatment arms.

The possible reasons for this are

- subversion of the randomisation system (inadequate concealment)
- erroneous generation of the randomisation list
- chance

The first of these can be discounted. The randomisation was operationalised using a centralised, web-based system which required the participant's details to be entered before the allocation was released. The system gave allocation sequentially; there was no option for users to obtain codes in a different order to the randomisation schedule. Three randomisation numbers were withdrawn in total; two were from the centres and were the result of the site PI erroneously entering test data onto the live system; a third was discontinued after the PI had entered their details twice (a "double click").

The second is unlikely. The randomisation system uses the same random number generator that is implemented in Stata and R among other packages (https://en.wikipedia.org/wiki/Mersenne_Twister). The system itself has been validated and our discussions with the system developers have reassured us this is implemented correctly, and although we plan to investigate the random number generator in the light of these findings we do not believe this to be at fault.

Below we address why we believe chance has caused the discrepancy.

5.2 Details of the randomisation

The breakdown of the allocation by site and by severity is in Table 34 and Table 35; a more detailed breakdown is in Table 36. This resulted in 63 possible combinations of site and severity of which 62 contained one or more participants, an average of 4·5 patients per combination (range 1 to 13). Within any combination of site and CAT the imbalance was never greater than two and within any completed block the imbalance was zero; for this reason, the blocking itself has worked. Almost all of the combinations contained incompletely filled blocks however, therefore making some imbalance likely. When summed up across all combinations of site and CAT these differences have become notable.

Table 34: Allocation by site

Site	CSLT	UC	AC	Total	UC minus AC
Ayr	4	3	2	9	1
Belfast	3	5	3	11	2
Cambridgeshire	5	5	3	13	2
Cwm Taf	5	3	1	9	2
Derbyshire	5	6	5	16	1
Dorset	5	4	3	12	1
Glasgow	8	8	6	22	2
Hull	5	6	4	15	2
Newcastle	4	6	5	15	1
Norfolk	2	3	5	10	-2
North Lincs	2	4	5	11	-1
North Bedford	4	4	3	11	1
Northampton	6	5	4	15	1
Northern	3	6	4	13	2
Nottinghamshire	7	6	6	19	0
Plymouth	4	3	1	8	2
Sheffield	6	5	4	15	1
Somerset	5	5	6	16	-1
South Bedford	5	6	5	16	1
Sunderland	5	3	2	10	1
Swansea	4	5	3	12	2
Total	97	101	80	278	21

Table 35: Allocation by severity

Stratum	CSLT	UC	AC	Total	UC minus AC
Mild (31-43)	42	41	36	119	5
Moderate (18-30)	30	33	23	86	10
Severe (5-17)	25	27	21	73	6
Total	97	101	80	278	21

Note: CSLT, computerised speech language therapy; UC, usual care; AC, attention control

Table 36: Detailed allocation by site and strata

Site	Severity (CAT)	CSLT	UC	AC	Total	*Imbalance**
Ayr	Mild (31-43)	2	1	1	4	*1*
	Moderate (18-30)	2	1	0	3	*2*
	Severe (5-17)	0	1	1	2	*1*
Belfast	Mild (31-43)	2	2	3	7	*1*
	Moderate (18-30)	0	2	0	2	*2*
	Severe (5-17)	1	1	0	2	*1*
Cambridgeshire	Mild (31-43)	1	0	1	2	*1*
	Moderate (18-30)	2	3	2	7	*1*
	Severe (5-17)	2	2	0	4	*2*
Cwm Taf	Mild (31-43)	3	2	1	6	*2*
	Moderate (18-30)	2	1	0	3	*2*
	Severe (5-17)	0	0	0	0	-
Derbyshire	Mild (31-43)	3	2	2	7	*1*
	Moderate (18-30)	1	2	1	4	*1*
	Severe (5-17)	1	2	2	5	*1*

Dorset	Mild (31-43)	2	1	2	5	*1*
	Moderate (18-30)	2	1	0	3	*2*
	Severe (5-17)	1	2	1	4	*1*
Glasgow	Mild (31-43)	3	3	1	7	*2*
	Moderate (18-30)	2	3	3	8	*1*
	Severe (5-17)	3	2	2	7	*1*
Hull	Mild (31-43)	2	3	2	7	*1*
	Moderate (18-30)	1	1	0	2	*1*
	Severe (5-17)	2	2	2	6	*0*
Newcastle	Mild (31-43)	2	2	1	5	*1*
	Moderate (18-30)	1	2	2	5	*1*
	Severe (5-17)	1	2	2	5	*1*
Norfolk	Mild (31-43)	0	1	2	3	*2*
	Moderate (18-30)	1	1	1	3	*0*
	Severe (5-17)	1	1	2	4	*1*
North Lincs	Mild (31-43)	1	1	2	4	*1*
	Moderate (18-30)	1	2	2	5	*1*
	Severe (5-17)	0	1	1	2	*1*
North SEPT	Mild (31-43)	0	1	1	2	*1*
	Moderate (18-30)	2	2	2	6	*0*
	Severe (5-17)	2	1	0	3	*2*
Northampton	Mild (31-43)	3	3	2	8	*1*
	Moderate (18-30)	1	1	1	3	*0*
	Severe (5-17)	2	1	1	4	*1*
Northern	Mild (31-43)	1	2	1	4	*1*
	Moderate (18-30)	1	2	2	5	*1*
	Severe (5-17)	1	2	1	4	*1*
Nottinghamshire	Mild (31-43)	3	3	3	9	*0*
	Moderate (18-30)	3	3	2	8	*1*
	Severe (5-17)	1	0	1	2	*1*
Plymouth	Mild (31-43)	2	1	1	4	*1*
	Moderate (18-30)	1	1	0	2	*1*
	Severe (5-17)	1	1	0	2	*1*
Sheffield	Mild (31-43)	3	2	1	6	*2*
	Moderate (18-30)	1	1	2	4	*1*
	Severe (5-17)	2	2	1	5	*1*
Somerset	Mild (31-43)	2	3	3	8	*1*
	Moderate (18-30)	1	1	1	3	*0*
	Severe (5-17)	2	1	2	5	*1*
South Beds	Mild (31-43)	4	5	4	13	*1*
	Moderate (18-30)	1	0	1	2	*1*
	Severe (5-17)	0	1	0	1	*1*
Sunderland	Mild (31-43)	2	1	1	4	*1*
	Moderate (18-30)	2	1	1	4	*1*
	Severe (5-17)	1	1	0	2	*1*
Swansea	Mild (31-43)	1	2	1	4	*1*

	Moderate (18-30)	2	2	0	4	*2*
	Severe (5-17)	1	1	2	4	*1*
Total		97	101	80	278	*21*

Note: * defined as the difference between the largest and smallest of the three groups within any combination of strata; CSLT, computerised speech language therapy; UC, usual care; AC, attention control

Big Cactus used randomly ordered blocks of size three and six, but it is easiest to illustrate this with a block of size three. In these, there are six possible combinations by which the three consecutive participants can be allocated a treatment arm (see Table 37). Wherever a block of size three was used on Big Cactus, these were indeed allocated one to each arm; the imbalance arose because too many blocks were left incomplete (here this would mean fewer than three participants), and those that were left incomplete tended to have attention control towards the end (i.e. block options 1 and 3).

Table 37: Possible allocations in a block of size three

Participant	Option 1	Option 2	Option 3	Option 4	Option 5	Option 6
First participant	CSLT	CSLT	UC	UC	AC	AC
Second participant	UC	AC	CSLT	AC	UC	CSLT
Third participant	AC	UC	AC	CSLT	CSLT	UC

Note: CSLT, computerised speech language therapy; UC, usual care; AC, attention control

We do nevertheless acknowledge that the attention control arm in particular has notably fewer participants than would be expected by the usual play of chance. Although we have no reason to doubt the authenticity of the randomisation generation algorithm, it is theoretically possible that this is at fault. Our belief is that the imbalance has occurred because the blocks one and three occurred – by chance –more among the less common combinations of CAT and site. We also note that the balance of the three arms was assessed as part of the ongoing trial reports and only became apparent in the closing months of the trial.

5.3 Implications of this for power in each arm

For the primary comparison of computerised therapy against usual care there is no reduction in power; indeed, the higher number in each of these arms led to a small increase for this comparison. For the comparison of attention control to usual care for conversation, under the same assumptions as the sample size calculation, we would have 60 and 73 evaluable patients. This would mean we would have 84% power. For the secondary endpoint of COAST we would now have 75% power.

5.4 Should we have used minimisation?

It is a matter of scientific opinion, and should be noted that the two authors of this document have differing views on this subject! Minimisation could have produced better balance overall but has been criticised by some for its predictability, particularly where the recruiting centre is included among the minimisation characteristics, although this is a matter of ongoing debate.[21-23] It was felt important to stratify by centre due to the availability of laptops at each centre as well as the potential for differential responses (case-mix) across centres, but minimisation would – at least to an extent – not guarantee balance without adding a deterministic element which, in turn, increases predictability or “sequence guessing”. Although imbalance had implications for the statistical power, we can be confident that the sequence here has not been guessed: no-one will have predicted the unusual sequence that resulted from this algorithm.

6 Description of the pragmatic design according to the PRECIS (Pragmatic-explanatory continuum indicator summary) domains

Table 38: Description of how pragmatic vs. explanatory the Big CACTUS trial was using the PRECIS-2 domains and scoring scale[24]

PRECIS-2 domain and descriptor	**Description of Big CACTUS trial**
Eligibility<br><br>To what extent are the participants in the trial similar to those who would receive this intervention if it was part of usual care?	**Equally pragmatic and explanatory**<br><br>The inclusion criteria relating to the participant's ability to engage with word finding therapy using computer software would be equally applicable in clinical practice (e.g. English language, sufficient vision and cognitive ability). Whereas the inclusion criteria relating to preventing contamination between groups and including only those more likely to respond to treatment are trial specific.
Recruitment<br><br>How much extra effort is made to recruit participants over and above what that would be used in the usual care setting to engage with patients?	**Rather explanatory**<br><br>Invitation letters were sent to potential participants, some of whom were still receiving usual care and some of whom had been discharged. Other participants were recruited through support groups.
Setting<br><br>How different is the setting of the trial and the usual care setting?	**Rather pragmatic**<br><br>Computer therapy was provided in the participants own home via SLT services at 20 sites across the UK.
Organisation<br><br>How different are the resources, provider expertise and the organisation of care delivery in the intervention arm of the trial and those available in usual care?	**Rather pragmatic**<br><br>The computer therapy is commercially available and already used by some SLT departments in the NHS. Therapy was delivered by SLTs who would usually treat this population. Additional support was provided by SLT assistants already working for the SLT department or local volunteers with an existing working relationship with the SLT department. Training for using the computer software was provided, but was comparable to that provided by the software designers.

Note: Table completed by one of the study team for the intervention arm only

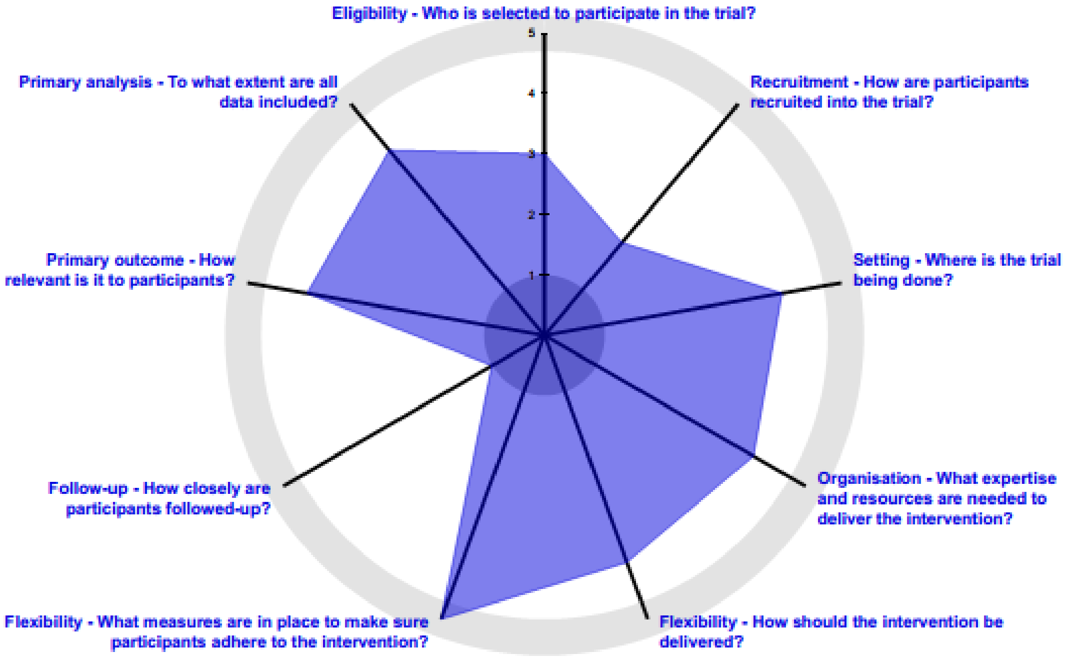

Figure 62: Illustrating how pragmatic vs. explanatory the Big CACTUS trial was using the PRECIS-2 domains and scoring scale.[24]

Note: Figure completed by one of the study team for the intervention arm only

7 Fidelity to CSLT, AC and UC interventions

7.1 Fidelity to the CSLT intervention

Fidelity measurement and results have been summarised in the table 39 below.

Table 39: Overall fidelity to the CSLT intervention

Measure of fidelity	Description of what was assessed	What was achieved
**Adherence to computer practice:**		
Total practice time	Electronic key file from StepByStep© software recorded date and time of practice (per protocol defined 10 hours as minimum for adherence to practice and 26 hours as a minimum for high adherence)	Mean = 28 h (SD 25.6 h)<br>Median = 21 h (IQR 4.9 h - 49.7 h)<br>45 (46%) participants practised for at least 26 hours<br>63 (64%) participants practised for at least 10 hours
Coverage	Number of participants in receipt of a computer with the StepByStep© software on it	97 (100%) participants received the computer software
**Quality of delivery:**		
Training of therapists	Central trial teams' training attendance record	25 (100%) therapists received training
Training of therapy assistants and volunteers by therapists at site	Therapists' documentation of training provision	83 (86%) participants received support from a therapy assistant or volunteer who had received training
Therapists' knowledge of intervention	Score out of 15 on therapist quiz	Median score of 10, rising to 12 over time
Tailoring of computer therapy according to language assessment results	Speech and language therapist with StepByStep approach expertise assessed rationale for tailoring (one participant per site, sample of 21 in total)	14 (66%) comprehensively understood<br>5 (24%) some understanding<br>2 (10%) no understanding
Provision of a therapy assistant or volunteer to provide support	Therapy assistant or volunteers' documentation of support provided (it was possible to decline support)	86 (89%) participants received support, 1 (1%) participant declined support and 10 (10%) had no documentation
Amount of support provided by therapy assistants or volunteers	Therapists' documentation of support provided (per protocol defined as a minimum of 1 h per month for a minimum of 4 months = 4 h)	Median 4.3 h (IQR 2.8 h – 5.6 h)
Content of support provided by therapy assistants or volunteers	Encouragement to use computer	85 (88%) participants received encouragement (median = 1.4 h [IQR 0.8 h – 2.3 h] per participant)
	Practising words in conversation/useful situations	73 (75%) participants received support to practise using words in conversation (median = 45 minutes [IQR 22 - 77.5 minutes] per participant)

Note: All means and medians relating to time were calculated across the six month intervention period

This is the subject of an ongoing PhD thesis which is exploring fidelity to CSLT in Big CACTUS in more depth.

7.2 Fidelity to the AC and UC interventions (results)

61% of AC participants received at least four puzzle books and four phone calls. Similar average amounts (in hours) of usual care were received by the intervention groups across the 6-month intervention period (CSLT, 3·2; UC, 3·8; AC, 3·2) suggesting no influence of different trial interventions on the usual care provided.

8 References

1. Bowen A, Hesketh A, Patchick E, Young A, Davies L, Vail A, *et al.* Clinical effectiveness, cost-effectiveness and service users' perceptions of early, well-resourced communication therapy following a stroke: a randomised controlled trial (the ACT NoW Study). 2012; 10.3310/hta16260. https://doi.org/10.3310/hta16260
2. Palmer R, Enderby P, Cooper C, Latimer N, Julious S, Paterson G, *et al.* Computer therapy compared with usual care for people with long-standing aphasia poststroke: a pilot randomized controlled trial. *Stroke* 2012;**43**:1904-11. https://doi.org/10.1161/STROKEAHA.112.650671
3. Julious S. Letter to the Editors. *Biometrics* 2004;**60**:284-. https://doi.org/doi:10.1111/j.0006-341X.2004.171_1.x
4. Julious SA, Owen RJ. Sample size calculations for clinical studies allowing for uncertainty about the variance. *Pharm Stat* 2006;**5**:29-37. https://doi.org/10.1002/pst.197
5. Breitenstein C, Grewe T, Flöel A, Ziegler W, Springer L, Martus P, *et al.* Intensive speech and language therapy in patients with chronic aphasia after stroke: a randomised, open-label, blinded-endpoint, controlled trial in a health-care setting. *The Lancet* 2017;**389**:1528-38. https://doi.org/10.1016/S0140-6736(17)30067-3
6. Richard C, Katz B, Hallowell C, Code E, Armstrong P, Roberts C, *et al.* A multinational comparison of aphasia management practices. *Int J Lang Commun Disord* 2000;**35**:303-14. https://doi.org/10.1080/136828200247205
7. Van Buuren S. Multiple imputation of discrete and continuous data by fully conditional specification. *Stat Methods Med Res* 2007;**16**:219-42. https://doi.org/10.1177/0962280206074463
8. Van Buuren S, Oudshoorn CGM. *Multivariate imputation by chained equations: MICE V1. 0 user's manual*: Leiden: TNO; 2000.
9. White IR, Royston P, Wood AM. Multiple imputation using chained equations: Issues and guidance for practice. *Stat Med* 2011;**30**:377-99. https://doi.org/doi:10.1002/sim.4067
10. Colantuoni E, Scharfstein DO, Wang C, Hashem MD, Leroux A, Needham DM, *et al.* Statistical methods to compare functional outcomes in randomized controlled trials with high mortality. *BMJ* 2018;**360**. https://doi.org/10.1136/bmj.j5748
11. Long A, Hesketh A, Paszek G, Booth M, Bowen A. Development of a reliable self-report outcome measure for pragmatic trials of communication therapy following stroke: the Communication Outcome after Stroke (COAST) scale. *Clin Rehabil* 2008;**22**:1083-94. https://doi.org/10.1177/0269215508090091
12. Bowen A, Hesketh A, Long A, Patchick E. *Scoring instructions for COAST and CaCOAST*. 2009. URL: http://www.click2go.umip.com/i/coa/coast.html (accessed August 8, 2016).
13. Long A, Hesketh A, Bowen A. Communication outcome after stroke: a new measure of the carer's perspective. *Clin Rehabil* 2009;**23**:846-56. https://doi.org/10.1177/0269215509336055
14. Van Reenen M, Janssen B. *EQ-5D-5L User Guide: Basic information on how to use the EQ-5D-5L instrument*. 2015. URL: https://euroqol.org/wp-content/uploads/2016/09/EQ-5D-5L_UserGuide_2015.pdf (accessed February 15, 2017).
15. Devlin NJ, Shah KK, Feng Y, Mulhern B, van Hout B. Valuing health-related quality of life: An EQ-5D-5L value set for England. *Health Econ* 2018;**27**:7-22. https://doi.org/10.1002/hec.3564
16. Group ICoHEEW. Statistical principles for clinical trials: ICH harmonized tripartite guideline. *Stat Med* 1999;**18**:1905-42.
17. Schulz KF, Altman DG, Moher D. CONSORT 2010 statement: updated guidelines for reporting parallel group randomised trials. *BMC Med* 2010;**8**:18. https://doi.org/10.1186/1741-7015-8-18
18. Ioannidis JP, Evans SJ, Gøtzsche PC, O'neill RT, Altman DG, Schulz K, *et al.* Better reporting of harms in randomized trials: an extension of the CONSORT statement. *Ann Intern Med* 2004;**141**:781-8. https://doi.org/10.7326/0003-4819-141-10-200411160-00009
19. Efron B. Bootstrap methods: another look at the jackknife. In: *Breakthroughs in statistics*: Springer; 1992:569-93. https://doi.org/10.1007/978-1-4612-4380-9_41
20. Hochberg Y, Tamhane A. In: Hochberg Y, Tamhane AC, editors. *Multiple Comparison Procedures* Hoboken, NJ, USA: John Wiley & Sons Inc; 1987. https://doi.org/doi:10.1002/9780470316672.ch6
21. Treasure T, MacRae KD. Minimisation: the platinum standard for trials?: Randomisation doesn't guarantee similarity of groups; minimisation does *BMJ : British Medical Journal* 1998;**317**:362-3.
22. Hills R, Gray R, Wheatley K. High probability of guessing next treatment allocation with minimisation by clinician. Controlled Clinical Trials, abstract no. 220, p. 70S-S.
23. Brown S, Thorpe H, Hawkins K, Brown J. Minimization—reducing predictability for multi-centre trials whilst retaining balance within centre. *Stat Med* 2005;**24**:3715-27. https://doi.org/doi:10.1002/sim.2391
24. Loudon K, Treweek S, Sullivan F, Donnan P, Thorpe KE, Zwarenstein M. The PRECIS-2 tool: designing trials that are fit for purpose. BMJ. 2015;350:h2147.
